# The HARMONIC trial: study protocol for a randomised controlled feasibility trial of Shaping Healthy Minds—a modular transdiagnostic intervention for mood, stressor-related and anxiety disorders in adults

**DOI:** 10.1136/bmjopen-2018-024546

**Published:** 2018-08-05

**Authors:** Melissa Black, Caitlin Hitchcock, Anna Bevan, Cliodhna O Leary, James Clarke, Rachel Elliott, Peter Watson, Louise LaFortune, Sarah Rae, Simon Gilbody, Willem Kuyken, David Johnston, Jill M Newby, Tim Dalgleish

**Affiliations:** 1 Medical Research Council Cognition and Brain Sciences Unit, University of Cambridge, Cambridge, UK; 2 Cambridgeshire and Peterborough NHS Foundation Trust, National Health Service, Fulbourn, UK; 3 Cambridge Institute of Public Health, University of Cambridge, Cambridge, UK; 4 Department of Health Sciences, University of York, York, UK; 5 Department of Psychiatry, University of Oxford, Oxford, UK; 6 School of Psychology, University of New South Wales, Sydney, New South Wales, Australia

**Keywords:** transdiagnostic, depression, anxiety, posttraumatic stress disorder, common mental health problems

## Abstract

**Introduction:**

Anxiety, mood and trauma-related disorders are common, affecting up to 20% of adults. Many of these individuals will experience symptoms of more than one disorder as diagnostically defined. However, most psychological treatments focus on individual disorders and are less effective for those who experience comorbid disorders. The Healthy and Resilient Mind Programme: Building Blocks for Mental Wellbeing (HARMONIC) trial introduces a novel transdiagnostic intervention (*Shaping Healthy Minds (SHM)*), which synthesises several evidence-based treatment techniques to address the gap in effective interventions for people with complex and comorbid difficulties. This early phase trial aims to estimate the efficacy and feasibility of the transdiagnostic intervention in preparation for a later-phase randomised controlled trial, and to explore mechanisms of change.

**Methods/analysis:**

We outline a patient-level two-arm randomised controlled trial (HARMONIC) that compares *SHM* to treatment-as-usual for individuals aged >18 years (n=50) with comorbid mood, anxiety, obsessive-compulsive or trauma/stressor disorders diagnoses, recruited from outpatient psychological services within the UK National Health Service (NHS). The co-primary outcomes will be 3-month follow-up scores on self-report measures of depressive symptoms, anxiety symptoms, and disability and functional impairment. Secondary outcomes include changes in symptoms linked to individual disorders. We will assess the feasibility and acceptability of *SHM*, the utility of proposed outcome measures, and refine the treatment manuals in preparation for a later-phase trial.

**Ethics and dissemination:**

This trial protocol has been approved by the Health Research Authority of the NHS of the UK (East of England, Reference: 16/EE/0095). We anticipate that trial findings will inform future revisions of clinical guidelines for numerous forms of mood, anxiety and stressor-related disorders. Findings will be disseminated broadly via peer-reviewed empirical journal articles, conference presentations, clinical workshops and a trial website.

**Trial registration:**

NCT03143634; Pre-results.

**Strengths and limitations of this study**

- The first study to investigate the feasibility and procedural uncertainties of a flexibly delivered modular transdiagnostic treatment protocol—*Shaping Healthy Minds (SHM)—*in adults with unipolar mood, anxiety and stressor-related disorders.
- This trial will provide a point estimate of efficacy of the *SHM* protocol, relative to treatment-as-usual (TAU), in preparation for a later-stage trial, and explore putative mediators and moderators of treatment outcome.
- Comparison of *SHM* against TAU currently provided by the National Health Service will provide a rigorous evaluation of treatment potential.
- Administering self-report questionnaires that are specific to each service user’s secondary diagnoses may limit the ability to draw group-based conclusions.

Mood, stressor-related, obsessive-compulsive and anxiety disorders—the so-called common mental health problems (CMHP; National Institute for Health and Care Excellence (NICE), 2011)—are one of the largest causes of disability in the world, with 16%–20% of adults affected at any given time.^1 2^ Maximising our ability to treat CMHP in cost-effective, efficient and effective ways that can be widely disseminated is a priority.^2^ At present, there is a range of complex psychological treatments with demonstrated efficacy in the treatment of CMHP, and in preventing recurrence. Consequently, NICE recommends psychological treatment at various points in the care pathway to all those suffering from such problems, although there are not specific recommendations for individuals experiencing more than one problem.^3^ Between 40% and 80% of patients experiencing a CMHP also experience an additional comorbid CMHP.^4 5^ Even our best available psychological treatments only achieve clinical recovery for 40%–70% of patients, depending on their primary CMHP, with people suffering complex comorbid conditions faring significantly worse.^6^ For the majority of patients who receive treatment, there remains a significant risk of future relapse.^7 8^ A key challenge therefore is how we can build on and extend beyond the current psychological treatments for CMHP to increase efficacy, and sustained recovery, particularly for those with comorbid, recurrent and complex presentations.^9^

Over the past decade, there has been a major shift in the conceptualisation of CMHP, away from a single-diagnosis approach in favour of a transdiagnostic model.^10 11^ There is strong empirical and theoretical support for development of transdiagnostic treatment approaches, as many of the cognitive, emotional, behavioural and interpersonal factors which drive symptomatology are consistent across disorders.^12 13^ A transdiagnostic approach thereby has the potential to improve the efficacy and efficiency of treatment for people with anxiety, stress and depression.

There are potential limitations to the commonly used single-disorder-focused treatment approach. First, with the exception of a few existing programmes,^14 15^ most evidence-based treatment protocols are single-disorder-focused programmes (eg, depression,^16^ generalised anxiety,^17 18^ social anxiety^19^ and post-traumatic stress disorder^20^). Comorbid conditions and disorders are either ignored, or minimally treated within these treatment packages. This leaves a mismatch between the available evidence base and the clinical reality which clinicians face: the majority of people with any given CMHP have at least one or more comorbid disorders to their primary diagnosis.^4^ Second, in the attempt to manualise treatments, most packages are inflexible ‘one-size-fits-all’ approaches, leaving patients with a wide range of problems and presentations receiving the same treatment package, regardless of their symptoms, goals and concerns.^14^ Third, in practice, many clinicians already deliver evidence-based psychological treatments in a flexible manner in order to address individual concerns and goals. Manualised treatments need to better reflect the realities of service user experiences and treatment delivery. This approach merits improvement so that delivered treatments are more efficient, effective and personalised to individuals’ concerns.

Existing psychological treatments for CMHP share more similarities than differences.^21^ Despite differences in the theoretical foundation underlying available psychological treatments, and the terms used to describe maintaining factors and treatment targets, there are many common elements. For instance, psychoeducation, graded exposure, mindfulness techniques and behavioural activation form a key component of a variety of effective treatments such as trauma-focused cognitive behavioural therapy (CBT), CBT, acceptance and commitment therapy (ACT), dialectical behaviour therapy (DBT), exposure therapy and mindfulness-based cognitive therapy (MBCT). The widespread availability of so many treatment options has the potential to elicit considerable decision-making difficulties for the treating clinician. In both formulation and treatment planning, challenging decisions occur when selecting the order in which to treat multiple difficulties, in evaluating the most appropriate treatment approach, and working out which treatment option will be acceptable and effective for the client.

A recent equivalence randomised controlled trial (RCT) demonstrated that a transdiagnostic protocol (The *Unified Protocol for Transdiagnostic Treatment of Emotional Disorders)* 22 and single-disorder protocols produced statistically equivalent reduction in severity of principal anxiety disorder diagnosis, but that there was less attrition in the transdiagnostic group.^23^ Promising results have also been found for other transdiagnostic treatment protocols, including Norton’s Transdiagnostic Group Cognitive Behavioural Therapy for anxiety,^15^ Gros’s Transdiagnostic Behaviour Therapy for affective disorders^24^ and Schmidt’s False Safety Behaviour Elimination Therapy for anxiety disorders.^25^ In addition, our systematic review and meta-analysis supported the overall efficacy of transdiagnostic treatments.^10^ The review called for more high-quality studies to resolve uncertainties surrounding the heterogeneity of treatment effects and to determine the best treatment approaches and designs. We aim to address these issues through evaluating a novel intervention which combines a number of evidence-based treatment strategies. In using a modular approach, this trial will contribute to identification and evaluation of effective treatment components and delivery method. The modular approach to treatment design incorporates self-contained functional units (therapy modules) that can operate independently and be delivered flexibly, and refer to other modules if needed.^22^ A complex, modular, tailored transdiagnostic intervention that targets common underlying processes maximises goodness of fit and has a direct focus on process rather than symptoms. The approach is thereby suitable for complexity and comorbidity as well as subsyndromal and prodromal symptoms.

The transdiagnostic intervention we have developed—Shaping Healthy Minds (*SHM*)*—*targets the processes and symptoms that are common to CMHPs and offers a number of advances in transdiagnostic treatment by incorporating the best available techniques from existing manual-based treatments into the one treatment package. A key aim of the programme is to encompass the treatment techniques that skilled psychologists and mental health clinicians already implement in standard practice for depressed, stressed and anxious patients with complex presentations.^26^ The intervention also builds on the Unified Protocol for Transdiagnostic Treatment for Emotional Disorders described by Barlow *et al*,^14^ by working towards a prescriptive approach for the delivery of treatment modules based on the formulation of the client’s presenting difficulties.^9^ Specifically, the treatment expands beyond interventions grounded in a sole treatment paradigm (eg, CBT) towards a theory-driven approach that uses efficacious techniques translated from basic science alongside components drawn from a wide range of evidence-based psychological treatments (eg, mindfulness-based interventions, ACT, behavioural activation and DBT).

The treatment protocol for *SHM* also changes the way that standardised manual-based treatments are delivered. Rather than using integral interventions (where all patients receive the same relatively fixed, complete protocol), the transdiagnostic intervention is a modular intervention, whereby the assessment of core problematic areas of emotional, cognitive, interpersonal and behavioural processes informs the selection and sequence of treatment modules targeted at specific problem areas for patients.^22^ This modular approach allows for standardised, yet flexible treatment that is personalised to the individual concerns, problems and goals for the patient. Finally, it expands beyond interventions that typically focus on alleviating negative symptomatology (eg, negative thoughts, excessive negative emotions) and incorporates interventions designed to increase positive emotions, capture strengths and enhance resilience for sustained recovery.

We aim to examine the feasibility of *SHM* in reducing symptoms of depression, anxiety, distress, disability and functional impairment through an early stage RCT, in line with recommendations for the development of complex interventions.^27^ In particular, we will gather data on the extent to which *SHM* performs comparably to treatment-as-usual (TAU) for a given service user’s primary diagnosed problem^10 28^ as well as for other significant additional, secondary and/or comorbid difficulties. In addition, this trial will provide a preliminary evaluation of whether a modular transdiagnostic treatment approach may be effective at reducing the distress and impairment associated with CMHP.^10^ The trial will also provide an indication of the feasibility and acceptability of the transdiagnostic intervention to service users and clinicians by recruiting through postprimary care UK National Health Service (NHS) psychology services where complex comorbidity represents the modal clinical presentation. In addition, the trial will provide initial estimates of cost-effectiveness in terms of service use and potential quality-adjusted life years (QALYs) added. We therefore present the protocol for a feasibility trial with co-primary outcomes, examining the effect of *SHM* on primary and comorbid diagnoses. The feasibility trial will provide a plausible range of point estimates of the efficacy of *SHM* on standardised continuous symptom measures for primary and secondary diagnoses to inform this key question, refine the treatment manual and contribute to the design of future scaled-up trials.^27^

## Methods and analysis

This trial protocol is written in compliance with the Standard Protocol Items: Recommendations for Interventional Trials guidelines.

### Study design

The design is a parallel-arm RCT comparing *SHM* to TAU. Participants will be assessed three times*—*at baseline, at post-treatment and at 3 months follow-up. These three time points involve face-to-face assessments including the full battery of primary and additional outcomes and process measures, described below.

### Participants and recruitment

The proposed feasibility study will seek to recruit 50 people aged 18 years and above with a primary diagnosis of a unipolar mood, anxiety, obsessive-compulsive, or trauma-related and stressor-related disorder (CMHPs) with at least one additional comorbid diagnosis according to the Diagnostic and Statistical Manual of Mental Disorders, Fifth Edition (DSM-5).^29^ Participants will be randomly allocated to one of two groups: (1) *SHM* or (2) TAU. Diagnosis of CMHPs will be determined by trained research staff using the Structured Clinical Interview for DSM-5 (SCID-5).^30^ To be eligible, participants will also need to score >10 on either the Patient Health Questionnaire (PHQ-9) or the Generalised Anxiety Disorder Questionnaire (GAD-7; see study measures below). Exclusion criteria are current/past psychosis or bipolar disorder, current diagnosis of alcohol or substance use disorder (all assessed via SCID-5^30^), organic brain damage, complex trauma history or recurrent self-injury requiring specialist services, or current suicidality that warrants immediate clinical attention and constitutes a current risk of harm to the individual (all assessed via participant report and the clinical care team). Participants may be engaged with the multidisciplinary clinical care team, but those randomised to *SHM* will not be receiving other psychological services while participating in the trial. All other services (eg, medication review with psychiatrist or general practitioner, occupational therapy, social support services) may be continued, and there are no medication exclusions.

Participants will be recruited through local NHS clinical psychology services, including the high-intensity team of the Cambridge Psychological Wellbeing Service, and secondary care services with expertise in treatment of more complex and comorbid affective disorders. The recruitment pathways will involve suitable service users on a waitlist to receive treatment being identified by a member of the clinical service (including an assistant psychologist focusing primarily on recruitment into clinical research studies) who will provide them with a letter outlining the study. Service users will then be able to contact the research team to opt into the study. Initial eligibility will be screened over the telephone, and suitable participants will be invited to complete the SCID, either at the clinical service or the research unit. At the beginning of this session, all participants will provide written informed consent (see supplementary materials for a sample Participant Information Sheet and Consent Form, online supplementary file 1). No potential participants will be contacted by a member of the research team until they have given consent for such contact to a member of the clinical care team.

10.1136/bmjopen-2018-024546.supp1 Supplementary data

### Participant allocation

**Figure 1:**
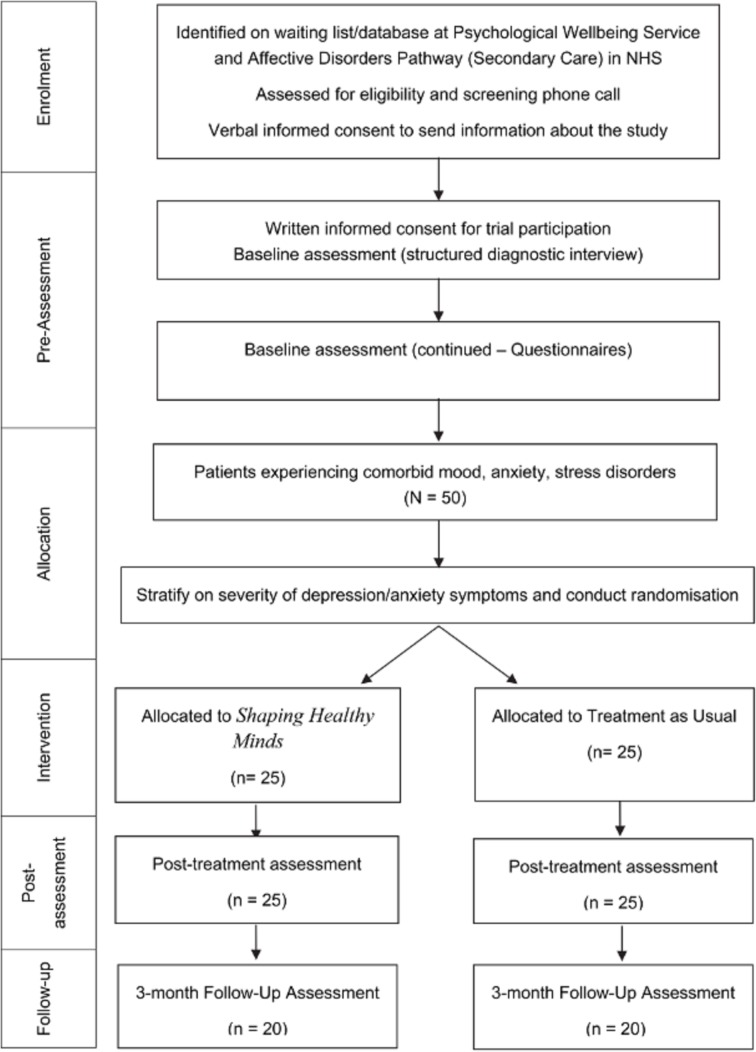
Participant flow diagram for the HARMONIC trial with anticipated participant numbers at each stage. NHS, National Health Service.

Following both baseline assessment sessions, eligible participants will be stratified according to depression (PHQ-9) and anxiety (GAD-7) severity scores and randomised to either *SHM* (n=25) or TAU (n=25). This will be achieved using computer-generated, quasi-random numbers and will be conducted by the trial statistician (PW), blind to study objectives. Once generated, this information is passed to the project coordinator responsible for delivering the intervention. Once a participant begins treatment, he or she is free to discontinue participation at any time, in which case she/he will be referred back to the appropriate NHS clinical care team. Figure 1 summarises participant flow through the trial.

### Interventions

*SHM* is a modular intervention, comprising 10 independent modules that will last up to 20 sessions. The content of the modules is drawn from a number of evidence-based psychological therapies, including CBT,^31^ ACT,^32^ DBT,^33^ MBCT^34^ and behavioural activation.^35 36^ The programme aims to bring together the core and unique therapeutic techniques from the best available disorder-focused treatment packages into the one transdiagnostic treatment package (eg, behavioural experiments^37^ and graded exposure^38^ from CBT, value exercises and mindfulness strategies from ACT,^39^ activity scheduling from BA,^35^ emotion regulation strategies from DBT,^33^ and present moment awareness exercises from MBCT).^40^ The elements of *SHM* were drawn from recent meta-analyses supporting the effectiveness of these treatment strategies^36 41–43^, and the manuals were written and reviewed by experienced clinical psychologists (TD, JMN, AB, CH and MB). In addition, experts in particular fields (eg, WK for mindfulness and case formulation) were consulted on the content of specific modules.

The modular approach is standardised, yet can be flexibly delivered according to an individual’s concerns, problems and goals.^22^ The treatment focuses both on alleviating negative symptoms and enhancing positive well-being, by teaching skills and techniques and enhancing positive emotions, harness and build on strengths, and maximise resilience over the longer term. Choice, order and length of modules (ie, number of sessions over which they are completed) are tailored to the transdiagnostic difficulties of the individual using collaborative case formulation,^44^ although there are three core modules that everyone receives (outlined below). Treatment consists of weekly face-to-face 1 hour sessions with the trial therapists. Sessions will involve collaboratively setting an agenda for the session based on the participants’ ratings for their top three problems and top three strengths, the goals set for therapy, and the module in focus. Participants will complete homework exercises to consolidate and practice the skills learnt during the specific modules and will be strongly encouraged to continue this practice following the end of one module and move to the next.

The 10 SHM modules (core modules listed in bold) are: (1) Getting acquainted with SHM. (2) Understanding emotions. (3) Managing and tolerating emotions. (4) Behavioural activation. (5) Tackling avoidance. (6) Tackling unhelpful thoughts. (7) Tackling unhelpful habits. (8) Overcoming repetitive thinking. (9) Managing upsetting memories and images. (10) Relapse prevention and future orientation. Additional information about the content of the modules can be found in the supplementary materials (online supplementary table 1).

10.1136/bmjopen-2018-024546.supp3 Supplementary data

### Treatment-as-usual

For TAU, clinical psychologists and high-intensity CBT therapists in teams specialising in CMHPs will be asked to provide the course of psychological therapy that they deem appropriate, in addition to referral to other health/social services and medication management. Psychological treatment in the specialist teams delivering TAU will standardly consist of disorder-focused CBT, Eye Movement Desensitisation Reprocessing or behavioural activation. The delivered treatment will be documented to ensure systematic understanding of the duration, frequency and type of treatment administered.

### Treatment integrity

Therapists with experience in treating adult CMHPs will deliver the transdiagnostic intervention. Treatment fidelity and clinician adherence for the *SHM* group will be established using continued monitoring of completion of module components and through independent rating of specific treatment strategies by the supervising clinical psychologist. After every session, clinicians will complete a bespoke Treatment Fidelity Checklist which is a session-by-session self-report measure of compliance with the *SHM* approach, and these will be evaluated during the weekly clinical supervision with the trial clinical supervisor. In addition, a randomly selected 25% of the audio-taped treatment sessions will be rated for adherence to the manuals by an experienced clinician, independent of the trial. Homework completion will be monitored by trial therapists.

This bespoke measure will be supplemented with the Cognitive Therapy Scale-Revised (CTS-R)—a standardised measure of competence within cognitive therapy, consisting of adherence to and skilful application of cognitive therapy methods and the therapeutic alliance.^45^ The CTS-R has 13 items that are completed by an independent rater, assessing agenda setting, feedback, collaboration, pacing and efficient use of time, interpersonal effectiveness, charisma/flair, facilitation of emotional expression, guided discovery, conceptualisation, identifying key cognitions, application of cognitive change methods, application of behavioural techniques, use of homework.

## Measures

### Co-primary outcomes

The co-primary outcome measures are self-reported symptoms of depression and anxiety, indexed by PHQ-9,^46^ and GAD-7,^47^ as well as levels of disability and functional impairment, indexed by the Work and Social Adjustment Scale.^48^

### Secondary outcomes

Given the transdiagnostic focus of the study, self-reported symptoms on specific disorders that clients meet criteria for at trial baseline will be indexed by the IAPT Phobia Scales (social phobia, agoraphobia, specific phobia),^49^ the Social Phobia Inventory,^50^ the Penn State Worry Questionnaire (generalised anxiety),^51^ the Obsessive-Compulsive Inventory,^52^ the Revised Impact of Event Scale (post-traumatic stress),^53^ the Agoraphobia-Mobility Inventory,^54^ the Fear Questionnaire (specific phobias),^55^ the Panic Disorder Severity Scale-self report version,^56^ the Health Anxiety Inventory-short version^57^ and the Sheehan Disability Scale.^58^ Participants will only complete a selection of these measures depending on their concerns and associated diagnoses. In addition to these disorder-specific measures, the Inventory of Depression and Anxiety Symptoms will capture both disorder-specific and transdiagnostic symptom dimensionality within a single measure.^59^ Selection of these measures will be determined following completion of the structured clinical interview at the beginning of assessment.

### Process measures

We will also include a number of process-related measures which will be administered at baseline, postintervention and at 3-month follow-up to begin to explore mechanisms of change and the feasibility of conducting embedded process outcome research within this type of trial (see table 1). To explore the value of the individual modules administered within the transdiagnostic intervention, we will also administer module-relevant measures (eg, rumination, distress tolerance) before and after completion of the module. Finally, participants’ expectancy of treatment outcomes and measures of engagement and compliance will be administered preintervention and postintervention to inform the further development of the protocol.

**Table 1:** Additional outcome and process measures to assess changes in potential mechanisms of psychological distress and in response to specific transdiagnostic intervention modules

Measure	Focus area
The Treatment Credibility/Expectancy Questionnaire (CEQ)^64^	Expectancy about treatment outcome, as well as the credibility of the treatment
Cognitive Emotion Regulation Questionnaire (CERQ)^65^	Ability to contextualise negative events within a wider frame of reference
The Experiences Questionnaire (EQ)^66^	Ability to disengage from troublesome mental content and take a more accepting stance towards it, as well as the tendency to engage in rumination
Differential Emotions Scale (DES^67^)	Intensity with which they experience different emotions on a typical day to obtain summary scores for positive emotions, negative emotions and denied emotions (the number of emotions *not* endorsed by the participant)
Levels of Personality Functioning Scale (LPFS)^68^	Personality functioning based on the Diagnostic and Statistical Manual of Mental Disorders, Fifth Edition (DSM-5) alternative model of personality disorders. It has four subscales: identity, self-direction, empathy and intimacy
Ruminative Responses Scale of the Response Styles Questionnaire (RRS)^69^	Rumination (Module 7—Overcoming repetitive thinking)
Distress Tolerance Scale (DTS)^70^	Ability to tolerate distress (Module 3—Managing and tolerating emotions)
Difficulties with Emotion Regulation Scale (DERS)^71^	Ability to label, perceive and regulate emotions (Modules 2—Understanding emotions and 3—Managing and tolerating emotions)
Dysfunctional Attitudes Scale (DAS) short form (versions 1 and 2)^72^	Negative beliefs, thoughts and assumptions (Module 6—Tackling unhelpful thoughts)
Kentucky Inventory of Mindfulness Skills (KIMS)^73^	Mindful awareness (Module 2—Understanding emotions)
Anxiety Sensitivity Index (ASI)^74^	Fear of physical anxiety sensations (Module 3—Managing and tolerating emotions and Module 5—Tackling avoidance)
Post-traumatic Cognitions Inventory–short version (PTCI)^75^	Trauma-related beliefs and maladaptive appraisals of intrusive symptoms (Module 9—Managing upsetting memories and images)
Skills of Cognitive Therapy^76^	Implementation of cognitive therapy skills (Module 6—Tackling unhelpful thoughts)
The Multidimensional Experiential Avoidance Questionnaire^77^	Avoidance of internal experiences including thoughts, feelings, physical sensations (Module 5—Tackling avoidance)

### Health economics measures

Data collection for the health economic evaluation will take a patient-level perspective,^60 61^ recording the cost per session of treatment and productivity losses resulting from time off work as a consequence of their mental health difficulties. Data will be collected using the Healthlines Resource Use Questionnaire,^60 61^ which is a measure of the participants' use of healthcare services (including NHS, help at home), occupational productivity (ie, time off work) and cost of transdiagnostic treatment delivery (eg, direct and indirect time spent in service delivery). The Medical Outcomes Study Questionnaire Short Form 36 Health Survey (SF-36),^62^ a generic quality of life questionnaire, will measure overall health and well-being, daily functioning and general life satisfaction across multiple domains. These measures allow calculation of the additional number of quality of life years the treatment will yield. These data will allow preliminary estimates of the potential cost utility of the transdiagnostic intervention and also of the feasibility of acquiring these data within the trial protocol.

## Methodological aspects

### Power analysis and sample size

Although a standard power calculation based on detecting treatment effects is the conventional approach to determining sample sizes for trials, the main aim of the current trial is to elucidate feasibility for a larger later-stage evaluation. We therefore sought at this stage only to provide a point estimate of the effect of *SHM* to inform a power calculation for this putative fully powered later-phase evaluation. Our previous experience with such early phase trial platforms indicates that 50 patients will provide sufficient numbers and diagnostic diversity to evaluate feasibility, acceptability and procedural uncertainty for *SHM* and a plausible test of recruitment protocols. This will give 40 patients (20 per arm) at 3-month post-treatment follow-up, assuming 20% attrition. This will provide a reasonable range of point estimates of effect on our set of candidate outcome measures sufficient to guide later phase trial work.

### Data collection and confidentiality

Outcome data for all participants who are randomised will be collected via face-to-face interviews and written questionnaires at baseline, post-treatment and 3-month follow-up. To maintain confidentiality, all participants will be given a trial number so that personally identifying information is not linked to assessment or trial information. All data (including personally identifiable information) will be stored on secure UK NHS databases, secure University of Cambridge servers and within locked filing cabinets under the management of the trial coordinator. Access will be limited to the immediate clinical research team.

### Blinding

Outcome assessments will be conducted by independent raters who have no therapeutic relationship with the patients and are blind to treatment condition. Double blinding of patients and therapists is not possible due to the nature of the trial (ie, a psychological intervention). Unblinding will not be necessary because participants and therapists are not blinded to intervention allocation.

### Statistical analysis plan

Initial analyses of the outcomes will be conducted by the trial statistician, blind to trial condition, following Consolidated Standards of Reporting Trials (CONSORT) standards (see online supplementary figure file 2). There are no planned interim analyses. Initial analyses will be conducted on an intention-to-treat basis, with subsequent analyses being per protocol. Mixed-model analyses of variance will be used to compare groups on outcomes at the three assessment points—baseline, postintervention and 3-month follow-up. Baseline levels on relevant measures will be included as covariates, as appropriate. Both intent-to-treat and per-protocol exploratory analyses will be conducted with our range of outcome measures following CONSORT standards. Multiple imputation will be used to account for missing data. Intent-to-treat analysis will also be used for those lost to attrition. Exploratory moderation and mediation analyses to examine process variables will be conducted using the MacArthur approach.^63^ For the health economic data, costs associated with service use will be calculated by attaching a unit cost to each instance of use, and data will be combined with QALYs^62^ derived from the SF-36 to arrive at a preliminary estimate of the cost utility of the transdiagnostic intervention.

10.1136/bmjopen-2018-024546.supp2 Supplementary data

### Monitoring and data management

The trial will take place at NHS sites and a research unit in the East of England. A Trial Management Group (TMG), will meet one to two times a year to: manage the protocol; monitor recruitment in relation to targets; deal with any adverse events; and coordinate the different stages of the project. The TMG consists of research clinical psychologists and assistant psychologists, a psychiatrist, a health economist, clinical psychology researchers, the trial statistician, a nurse practitioner and a service user representative. Day-to-day project management will be the responsibility of a smaller trial team, meeting fortnightly to deal with administrative issues, troubleshooting and recruitment flow. Clinical supervision will take place fortnightly. As this is a phase I/II trial, a data management committee was deemed unnecessary, and as such the trial team are responsible for monitoring and data management. Data will be monitored for completeness and consistency using spot checks and plausibility checks carried out by the trial statistician. The trial lead, trial coordinator and statistician will have full access to the final trial data set. The study data will be reported in line with the current CONSORT recommendations.

### Patient and public involvement

Most broadly, the driving force behind the development of *SHM* has been feedback from many hundreds of service users in clinical settings, specifically related to the suitability of treatments for complex and comorbid CMHPs. A Lived Experience Group comprising service users and carers hosted by the Cambridge Centre for Affective Disorders discussed the details of the current study at its meeting on 12 November 2013. This group provided useful feedback to the research team in terms of the materials for service users (eg, consent forms), the clinical setting for the intervention, and optimal forms of Patient and Public Involvement (PPI) involvement (specifically, the use of service user researchers to conduct qualitative interviews with service users). Further, we received service user input on the content of the draft treatment modules, and a part of this feasibility trial will be receiving feedback from participants on their experience of participating in the trial and using the manuals. We will send all participants a report describing the findings and their implications. We will also make participants aware of the study website. There will be a number of roles following completion of recruitment including the refinement and revision of the treatment manual along with involvement in the academic output preparation.

## Ethics and dissemination

### Ethical approval and protocol amendments

The study will be conducted within appropriate UK MRC, NHS and professional ethical guidelines, ensuring that Good Clinical Practice procedures are adhered to at all times. Protocol amendments will be circulated to the ethics committee, and trial team, and published in the online registration of the trial, and in the trial paper.

### Safety aspects

Adverse events are managed in line with UK Medical Research Council (MRC) protocols and, in the unlikely case of an adverse event, will be documented appropriately. Precautions have been taken to reduce the likelihood of adverse events occurring; for example, patients who are acutely suicidal or at high risk of harm do not meet study inclusion criteria. The interventions are delivered by therapists experienced in the management of risk and in the treatment of psychological disorders. In the case of any adverse events as a result of the intervention that would interfere with participation, participation in the trial will be discontinued. Regular team meetings will be conducted to monitor any difficulties patients may be having and ways of best dealing with these difficulties. Serious adverse events will be reported to the ethics committee. The trial is underwritten by the University of Cambridge in the case that any individual suffers harm or requires post-trial care. Any adverse events will be reported in the trial paper.

### Dissemination policy

There are no publication restrictions and findings will be disseminated broadly to participants, healthcare professionals, the public and other relevant groups. Academic outputs will take the form of peer-reviewed empirical journal articles, commentary pieces and conference presentations. Clinical outputs will be prioritised by the research team in order to maximise the impact of the findings with practitioners and commissioners. Outputs will comprise clinical conferences, workshops, service user groups and a study website that will make the intervention materials and related measures generally available. We will send all participants a report describing the findings and their implications. We will also make participants aware of the study website. We anticipate that trial findings will inform future revisions of clinical guidelines for numerous forms of mood, anxiety and stress disorders, and the development of guidelines for comorbid conditions. Anonymised data from the trial will be made publicly available on an open access database.

## Discussion

A significant proportion of the cost of CMHPs is generated by adults suffering from complex and comorbid depression, stress and anxiety, where treatment non-response, cross-sector service use across health, social care and housing, and loss of productivity are greatest. Providing effective interventions for these mental health problems therefore has the potential to reduce both long-term treatment costs as well as prevent large productivity losses. At present, most psychological interventions focus on specific diagnoses and many treatment manuals take a ‘one-size-fits-all’ approach. Current evidence-based interventions only achieve clinical recovery for 40%–70% of patients, with people suffering complex comorbid conditions faring significantly worse. This randomised controlled feasibility trial aims to pave the way for a scaled-up efficacy trial of a new transdiagnostic modular treatment for all CMHPs—*SHM—*that enables the flexible delivery of evidence-based techniques. This treatment approach may improve the effectiveness and dissemination of evidence-based interventions for the many individuals for whom diagnosis-specific treatments leave significant difficulties unaddressed. The results from this trial will provide a range of estimates of effect sizes that can be used to power a later-stage trial of treatment efficacy, to refine the treatment protocol and to inform future evaluation of the mechanisms underlying any treatment effects. If effective, *SHM* has the potential to improve outcome for those with complex presentations, through offering a cost-effective treatment option to reduce chronic, transdiagnostic psychological difficulties.

### Trial status

This trial was registered at clinicaltrials.gov on 4 May 2017 (NCT03143634). This article was submitted on 31 May 2018. To date, 15 participants have met eligibility criteria for the study and have been randomised to a condition. The trial opened on 31 July 2017, and data collection aims to be completed by September 2019.

## Supplementary Material

Reviewer comments

Author's manuscript
