# Supplementary material for: The HARMONIC trial: study protocol for a randomised controlled feasibility trial of Shaping Healthy Minds—a modular transdiagnostic intervention for mood, stressor-related and anxiety disorders in adults
Source: BMJ Open. 2018 Aug 5;8(8):e024546. doi: 10.1136/bmjopen-2018-024546 (PMC6078277; doi:10.1136/bmjopen-2018-024546)
Supplement: Supplementary data [file bmjopen-2018-024546supp001.pdf]

Participant Information Sheet
Study title: *Shaping Healthy Minds*

We invite you to participate in a study investigating a new modular treatment for mental health difficulties. Please read this information sheet if you wish to hear about the study in more detail. Your participation is *entirely voluntary*. Your treatment through the NHS will not be affected if you decide not to participate in this research.

This treatment – *Shaping Healthy Minds* (SHM) – treats symptoms that are common across depression and multiple anxiety disorders. The treatment consists of components of the best available evidence-based psychological treatments (e.g., mindfulness-based interventions, Acceptance and Commitment Therapy, Cognitive Behavioural Therapy, Behavioural Activation, and Dialectical Behaviour Therapy). Through using components of different types of therapy, the SHM aims to not only treat the principle problem that each individual is experiencing (e.g., depression or anxiety) but to target other psychological symptoms the individual is experiencing at the same time. In this way, someone who is experiencing both depression and anxiety will receive treatment for both of these issues, in contrast to the usual approach of treating one problem first, using only one type of therapy.

Purpose of the study
The purpose of this study is test whether the SHM is an effective treatment for people experiencing multiple psychological difficulties. We are interested in whether SHM will reduce symptoms of multiple mental health issues in those who are suffering from both depression and one or more anxiety disorders.

What's involved?
If you decide to take part, you would be asked to attend a 'screening' session to determine whether you are eligible to participate in this study. It will take 60-90 minutes and involves interviews and questionnaires focusing on emotions and mental health.

If you are eligible to participate in the trial you will then be randomly allocated to one of two groups:

(i) 15-20 sessions of the *Shaping Healthy Minds* (SHM) programme,
(ii) Treatment you would usually receive from the NHS (Treatment-as-Usual)

You will <u>not</u> be able to choose which of these groups you are allocated to, as the allocation is random and decided by a computer.

In the SHM programme, you will complete individual sessions with a clinical psychologist. The exact therapy components that you complete will be selected in consultation with you and based on the particular symptoms you are experiencing. That is, the treatment will be shaped for you. You will also receive the regular NHS care that you normally receive except for any psychological therapy.

In Treatment-as-Usual, you will receive all of the regular NHS services you would normally receive including any psychological therapy.

In addition to receiving treatment sessions, you will be asked to attend five assessment sessions where you will be asked to complete some questionnaires, some computer-based tasks that ask about your memories and emotions, including a task that involves pleasant and unpleasant images. In addition, there is an **optional** neuroimaging task, involving two functional magnetic resonance imaging scans (before and after treatment).

You can choose not to complete the imaging sessions and still participate in the study.

Prior to completing the treatment sessions (SHM or Treatment-as-Usual) which form the main part of the study, you will be asked to complete one assessment session which involves interviews and questionnaires, and one assessment session with computer tasks and the optional imaging session. You would then repeat these two assessment sessions after completing the treatment, followed by one final assessment session 3 months following the completion of treatment. Each assessment session will take approximately one and a half hours, which includes a break if needed. With your consent, some sessions will be audio-recorded to allow checks on the quality of the treatment you are receiving.

Why have I been invited to take part?
All individuals currently receiving NHS services for depression or anxiety in the Cambridge area are being invited to participate in this study.

Do you have to take part?
No, it is up to you to decide. We will describe the study and go through this information sheet, which we will then give to you. If you do want to join in we'll ask you to sign a consent form, a copy of which you can keep along with this information sheet. You are free to withdraw from the study at any point *without giving us a reason*. You will not be treated any differently by any NHS service if you choose not to participate in this study or if you decide to withdraw.

Will I be reimbursed?
You will be reimbursed for the assessment sessions at a rate of £6 per hour for your time. It is anticipated that the one screening session and four assessment sessions will not exceed a total of ten hours, for which you would receive a minimum of £45, plus travel costs. If you choose to do the neuroimaging sessions as well, you will receive £20 per imaging session, so you would receive a total of £85. You will not be reimbursed for the therapy sessions as you will be receiving therapy free of charge by qualified clinical psychologists.

Are there any risks or benefits associated with taking part?
All of the tasks, interviews, and questionnaires we will ask you to complete have been used safely in previous research. As with any research involving emotional material, there is a chance that you will experience some upset when you discuss personal memories and difficulties. In our experience this is usually very mild and short-lived with no lasting ill effects. After participation you will receive a complete and thorough explanation of the study and you will be encouraged to express your feelings about your experience, if you wish. Additionally, you will be able to contact a member of the research team (a qualified clinical psychologist) after any session should you feel you are experiencing distress as a result of taking part in the study.

***What are the possible benefits of taking part*?**
We hope that the findings from this research will lead to improvement in treatment options for people experiencing multiple mental health disorders. We also hope that completing the SHM, if you are randomly allocated to that treatment, will have a beneficial impact on your psychological difficulties. We cannot guarantee that this will be the case for everybody who takes part, but you will have an opportunity to experience a range of therapeutic strategies and techniques.

What if there is a problem?
For any complaint about the way you have been dealt with during the study or any possible harm you may have suffered you can contact the Patient Advice and Liaison Service on 01223 726 774, http://www.cpft.nhs.uk/about-us/pals.htm

Further Information & Contact Details:
If you would like any further information about this project please contact the research coordinator **Dr Melissa Black** at the MRC Cognition and Brain Sciences Unit (Tel: **01223 273 739**; Email: **melissa.black@mrc-cbu.cam.ac.uk**)

Thank you for reading this information sheet

Participant ID Number:

Participant Consent Form (Version 2.2, April 2018)

Title of the project: Shaping Healthy Minds

Name of project coordinator: Dr Melissa Black

Please initial box:

Statement	Initials
1. I confirm that I have read and understood the Information Sheet for the above study. I have had the opportunity to consider the information, ask questions and have had these answered satisfactorily.	
2. I understand that my participation is voluntary and that I am free to withdraw at any time without giving any reason and without my medical care or legal rights being affected.	
3. I understand that data (including audio recordings) collected from me during the study may be looked at by individuals from the research team where it is relevant to my taking part in this research. I give permission for these individuals to have access to these data.	
4. I understand that my GP will be informed of my participation in this study if s/he is not already aware of it.	
5. I agree to take part in the above study.	
6. I agree to a researcher contacting me after the end of this study about possible future research (optional).	

………………………………………… ………….. …………………………..

Name of Participant Date Signature

………………………………………… ………….. ………………… ……….

Name of Person taking consent Date Signature

(When the form is completed, 1 copy is for the participant and 1 is for the study files)
