# Supplementary material for: The HARMONIC trial: study protocol for a randomised controlled feasibility trial of Shaping Healthy Minds—a modular transdiagnostic intervention for mood, stressor-related and anxiety disorders in adults
Source: BMJ Open. 2018 Aug 5;8(8):e024546. doi: 10.1136/bmjopen-2018-024546 (PMC6078277; doi:10.1136/bmjopen-2018-024546)
Supplement: Supplementary data [file bmjopen-2018-024546supp003.pdf]

Supplementary Table 1.

*Key treatment components of Shaping Healthy Minds Core and Optional Modules.*

| # | Module Title | Sections of Module | Duration (sessions) |
|---|---|---|---|
| 1 | Getting Acquainted with *Shaping Healthy Minds* *Core Module* | • Education about depression and anxiety, and about emotions (Including 5-part model of emotion episodes)<br>• Orientation to treatment<br>• Defining Top 3 Problems and Top 3 Strengths<br>• Setting treatment goals and making plans<br>• Enhancing motivation | 1-3 |
| 2 | Understanding emotions *Core Module* | • Education about emotions<br>• Orientation to the emotion model<br>• Self-monitoring of emotion episodes<br>• Introduction to Mindful Awareness of Emotions | 1-2 |
| 3 | Managing and Tolerating Emotions *Optional Module* | • Education about emotion management toolbox<br>• Relaxation<br>• Exercise<br>• Social Support<br>• Distraction<br>• Self-soothing<br>• Accepting and Tolerating Feelings | 1-3 |
| 4 | Behavioural Activation *Optional Module* | • Education about low activity cycle<br>• Activity Monitoring<br>• Activity Scheduling and establishing positive routines<br>• Savouring the good things | 1-2 |
| 5 | Tackling Avoidance *Optional Module* | • Education about avoidance<br>• In vivo exposure<br>• Interoceptive exposure<br>• Emotion exposures | 1-3 |
| 6 | Tackling unhelpful thoughts *Optional Module* | • Education about thoughts<br>• Thought monitoring<br>• Thought questioning and cognitive flexibility<br>• Behavioural experiments<br>• Accepting and tolerating thoughts | 1-3 |

| | | | |
|---|---|---|---|
| 7 | Tackling unhelpful habits<br>*Optional Module* | • Education about habits<br>• Self-monitoring of habits<br>• Step-by-step habit change process | 1-3 |
| 8 | Overcoming Repetitive Thinking<br>*Optional Module* | • Education about rumination and worry<br>• Self-monitoring of rumination and worry<br>• Getting unstuck toolbox<br>• Shifting thinking styles and perspective, directing attention | 1-4 |
| 9 | Managing upsetting memories and images<br>*Optional Module* | • Education about memories and intrusive images<br>• Monitoring of memories<br>• Imaginal exposure<br>• Rescripting memories<br>• Reliving + rescripting memories | 2-4 |
| 10 | Preventing relapse and building a positive future<br>*Core Module* | • Education about lapses and relapses<br>• Evaluating treatment progress and consolidating learning<br>• Creating a therapy blueprint (relapse prevention plan)<br>• Setting goals and making future plans | 1 |
