# Supplementary material for: The HARMONIC trial: study protocol for a randomised controlled feasibility trial of Shaping Healthy Minds—a modular transdiagnostic intervention for mood, stressor-related and anxiety disorders in adults
Source: BMJ Open. 2018 Aug 5;8(8):e024546. doi: 10.1136/bmjopen-2018-024546 (PMC6078277; doi:10.1136/bmjopen-2018-024546)
Supplement: Supplementary data [file bmjopen-2018-024546supp002.pdf]

HARMONIC Trial (Healthy and Resilient Mind Programme: Building Blocks for Mental Wellbeing)

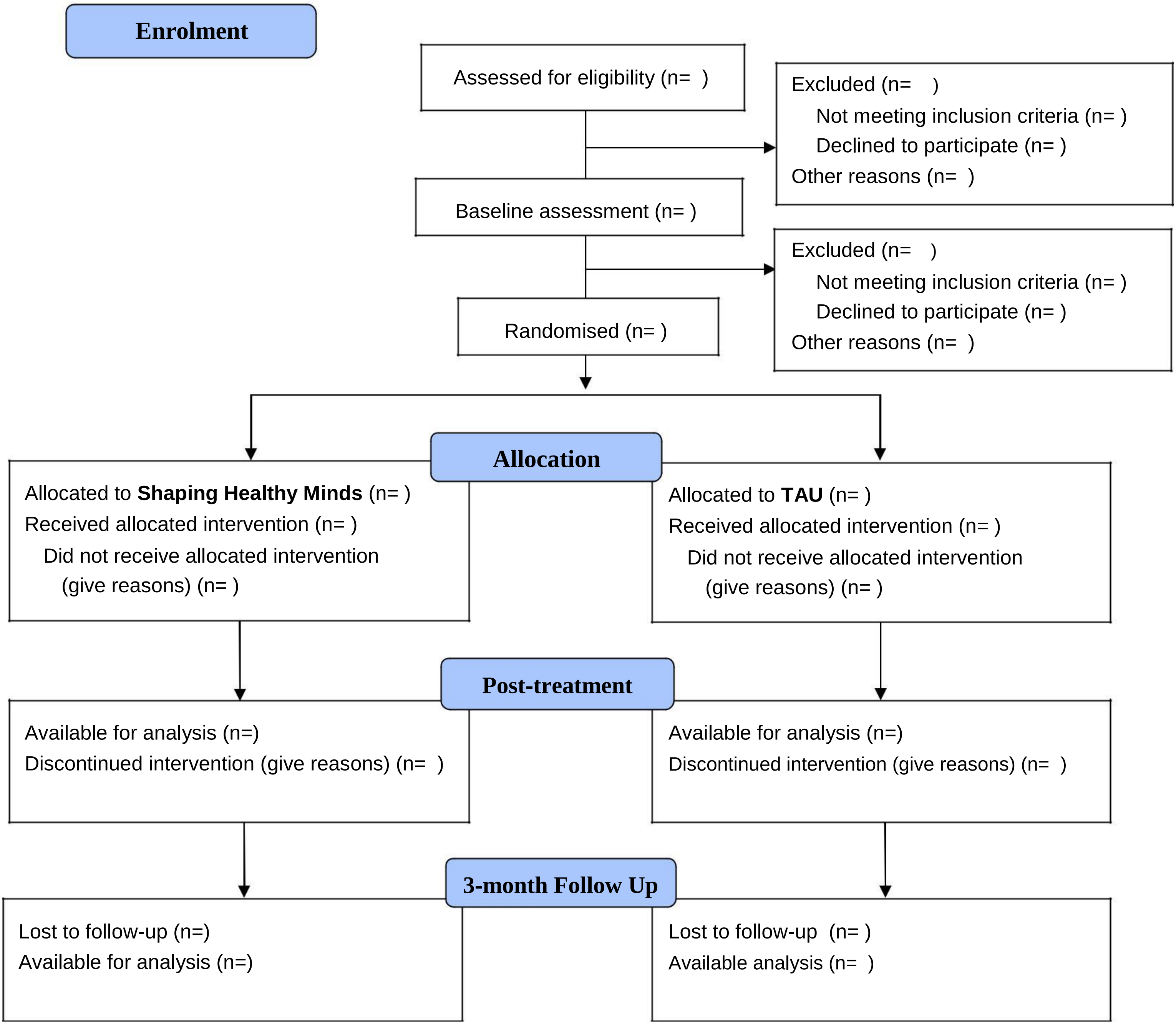

Figure 1. CONsolidated Standards of Reporting Trials (CONSORT) diagram
