# Supplementary material for: The HARMONIC trial: study protocol for a randomised controlled feasibility trial of Shaping Healthy Minds—a modular transdiagnostic intervention for mood, stressor-related and anxiety disorders in adults
Source: BMJ Open. 2018 Aug 5;8(8):e024546. doi: 10.1136/bmjopen-2018-024546 (PMC6078277; doi:10.1136/bmjopen-2018-024546)
Supplement: Author's manuscript [file bmjopen-2018-024546.draft_revisions.pdf]

BMJ Open

BMJ Open is committed to open peer review. As part of this commitment we make the peer review history of every article we publish publicly available.

When an article is published we post the peer reviewers' comments and the authors' responses online. We also post the versions of the paper that were used during peer review. These are the versions that the peer review comments apply to.

The versions of the paper that follow are the versions that were submitted during the peer review process. They are not the versions of record or the final published versions. They should not be cited or distributed as the published version of this manuscript.

BMJ Open is an open access journal and the full, final, typeset and author-corrected version of record of the manuscript is available on our site with no access controls, subscription charges or pay-per-view fees (http://bmjopen.bmj.com).

If you have any questions on BMJ Open's open peer review process please email info.bmjopen@bmj.com

BMJ Open

The HARMONIC trial: Study protocol for a randomised controlled feasibility trial of Shaping Healthy Minds – a modular transdiagnostic intervention for mood, stressor-related and anxiety disorders in adults

Journal:	*BMJ Open*
Manuscript ID	bmjopen-2018-024546
Article Type:	Protocol
Date Submitted by the Author:	31-May-2018

Complete List of Authors:	Black, Melissa; MRC Cognition and Brain Sciences Unit, University of Cambridge; Cambridgeshire and Peterborough NHS Foundation Trust<br>Hitchcock, Caitlin; University of Cambridge, MRC Cognition and Brain Sciences Unit; Cambridgeshire and Peterborough NHS Foundation Trust<br>Bevan, Anna; MRC Cognition and Brain Sciences Unit, University of Cambridge<br>O Leary, Cliodhna; University of Cambridge, MRC Cognition and Brain Sciences Unit; Cambridgeshire and Peterborough NHS Foundation Trust<br>Clarke, James; Cambridgeshire and Peterborough NHS Foundation Trust<br>Elliott, Rachel; MRC Cognition and Brain Sciences Unit, University of Cambridge; Cambridgeshire and Peterborough NHS Foundation Trust<br>Watson, Peter; MRC Cognition and Brain Sciences Unit<br>LaFortune, Louise; University of Cambridge, Institute of Public Health<br>Rae, Sarah; Cambridgeshire and Peterborough NHS Foundation Trust<br>Gilbody, Simon; The University of York, Department of Health Sciences<br>Kuyken, Willem; University of Oxford, Department of Psychiatry<br>Johnston, David; MRC Cognition and Brain Sciences Unit, University of Cambridge; Cambridgeshire and Peterborough NHS Foundation Trust<br>Newby, Jill M.; UNSW, Clinical Research Unit for Anxiety and Depression<br>Dalgleish, Tim; Medical Research Council Cognition and Brain Sciences Unit; Cambridgeshire and Peterborough NHS Foundation Trust

Keywords:	Transdiagnostic, Depression, Anxiety, Posttraumatic Stress Disorder, Common Mental Health Problems

SCHOLARONE™
Manuscripts

The HARMONIC trial: Study protocol for a randomised controlled feasibility trial of *Shaping Healthy Minds* – a modular transdiagnostic intervention for mood, stress and anxiety disorders in adults

Melissa Black[1,2]
Caitlin Hitchcock[1,2]
Anna Bevan[1]
Cliodhna O Leary[1,2]
James Clarke[2]
Rachel Elliott[1,2]
Peter Watson[1]
Louise Lafortune[5]
Sarah Rae[2]
Simon Gilbody[6]
Willem Kuyken[4]
David Johnston[1,2]
Jill Newby[3]
Tim Dalgleish[1,2]

1. Medical Research Council Cognition and Brain Sciences Unit, University of Cambridge, Cambridge UK
2. Cambridgeshire and Peterborough NHS Foundation Trust
3. School of Psychology, UNSW Sydney
4. Department of Psychiatry, University of Oxford
5. Cambridge Institute of Public Health, University of Cambridge
6. Department of Health Sciences, University of York

Corresponding Author: Tim Dalgleish (tim.dalgleish@mrc-cbu.cam.ac.uk)

Trial Registration: NCT03143634 (ClinicalTrials.gov)
Protocol Version: First, 31/05/2018
Funding: National Institute of Health Research, Research for Patient Benefit (RfPB: PB-PG-0214-33072)

Word count: 4482 words

For peer review

Abstract

Introduction: Anxiety, mood and trauma-related disorders are common, affecting up to 20% of adults. Many of these individuals will experience symptoms of more than one disorder as diagnostically defined. However, most psychological treatments focus on individual disorders and are less effective for those who experience comorbid disorders. The HARMONIC trial introduces a novel transdiagnostic intervention (*Shaping Healthy Minds*), which synthesises several evidence-based treatment techniques to address the gap in effective interventions for people with complex and comorbid difficulties. This early-phase trial aims to estimate the efficacy and feasibility of the transdiagnostic intervention in preparation for a later-phase randomised controlled trial, and to explore mechanisms of change.

Methods/Analysis: We outline a patient-level two-arm randomised controlled trial (HARMONIC) that compares *Shaping Healthy Minds* to treatment-as-usual (TAU) for individuals aged >18 years (N=50) with co-morbid mood, anxiety, obsessive-compulsive or trauma/stressor disorder diagnoses, recruited from outpatient psychological services within the UK National Health Service. The co-primary outcomes will be 3-month follow-up scores on self-report measures of depressive symptoms, anxiety symptoms, and disability and functional impairment. Secondary outcomes include changes in symptoms linked to individual disorders. We will assess the feasibility and acceptability of *Shaping Healthy Minds*, the utility of proposed outcome measures, and refine the treatment manuals in preparation for a later-phase trial.

Ethics and dissemination: This trial protocol has been approved by the Health Research Authority of the National Health Service of the United Kingdom (East of England, Reference: 16/EE/0095). We anticipate that trial findings will inform future revisions of clinical guidelines for numerous forms of mood, anxiety, and stressor-related disorders. Findings will be disseminated broadly via peer-reviewed empirical journal articles, conference presentations, clinical workshops, and a trial website.

For peer review only

Key Words: Transdiagnostic, Depression, Anxiety, Posttraumatic Stress Disorder, Common Mental Health Problems

Trial registration: Clinicaltrials.gov identifier: NCT03143634. This trial protocol is written in compliance with the Standard Protocol Items: Recommendations for Interventional Trials (SPIRIT) guidelines.

For peer review only

Strengths and limitations of this study

- The first study to investigate the potential efficacy of a flexibly-delivered modular transdiagnostic treatment protocol in adults with unipolar mood, anxiety, and trauma-related disorders.
- Investigating the feasibility and procedural uncertainties associated with the transdiagnostic intervention – *Shaping Healthy Minds* – in preparation for a scaled-up clinical trial.
- Evaluation of *Shaping Healthy Minds* against treatment-as-usual (TAU) currently provided by the NHS will determine the potential value of the new protocol.
- This trial aims to provide a point estimate of efficacy of the *Shaping Healthy Minds* protocol, relative to TAU, in preparation for a later-stage trial, and to explore putative mediators and moderators of treatment outcome.

The HARMONIC trial: Study protocol for a randomised controlled feasibility trial of *Shaping Healthy Minds* – a modular transdiagnostic intervention for mood, stressor-related and anxiety disorders in adults

Mood, stressor-related, obsessive-compulsive and anxiety disorders – so called common mental health problems (CMHP; NICE, 2011) – are one of the largest causes of disability in the world, with 16 – 20% of adults affected at any given time [1, 2]. Maximising our ability to treat CMHP in cost effective, efficient, and effective ways that can be widely disseminated is a priority [2]. At present, there is a range of complex psychological treatments with demonstrated efficacy in the treatment of CMHP, and in preventing recurrence. Consequently, the National Institute of Health and Care Excellence (NICE) recommends psychological treatment at various points in the care pathway to all those suffering from such problems, although there are not specific recommendations for individuals experiencing more than one problem [3]. Between 40 – 80% of patients experiencing a CMHP also experience an additional comorbid CMHP [4, 5]. Even our best available psychological treatments only achieve clinical recovery for 40 – 70% of patients, depending on their primary CMHP, with people suffering complex co-morbid conditions faring significantly worse [6]. For the majority of patients who receive treatment, there remains a significant risk of future relapse [7, 8]. A key challenge therefore is how we can build on and extend beyond the current psychological treatments for CMHP to increase efficacy, and sustained recovery, particularly for those with co-morbid, recurrent, and complex presentations [9].

Over the past decade, there has been a major shift in the conceptualisation of CMHP, away from a single-diagnosis approach in favour of a transdiagnostic model [10, 11]. There is strong empirical and theoretical support for development of transdiagnostic treatment approaches, as many of the cognitive, emotional, behavioural, and interpersonal factors which drive symptomology are consistent across disorders [12, 13]. A transdiagnostic approach

For peer review only

thereby has the potential to improve the efficacy and efficiency of treatment for people with anxiety, stress and depression.

There are potential limitations to the commonly-utilised single-disorder-focused treatment approach. First, with the exception of a few existing programmes [14, 15], most evidence-based treatment protocols are single-disorder-focused programs (e.g., depression [16], generalised anxiety [17, 18], social anxiety [19], and post-traumatic stress disorder [20]). Comorbid conditions and disorders are either ignored, or minimally treated within these treatment packages. This leaves a mismatch between the available evidence base and the clinical reality which clinicians face: the majority of people with any given CMHP have at least one or more comorbid disorders to their primary diagnosis [4]. Second, in the attempt to manualise treatments, most packages are inflexible 'one-size-fits-all' approaches, leaving patients with a wide range of problems and presentations receiving the same treatment package, regardless of their symptoms, goals, and concerns [14]. Third, in practice, many clinicians already deliver evidence-based psychological treatments in a flexible manner in order to address individual concerns and goals. Manualised treatments need to better reflect the realities of service-user experiences and treatment delivery. This approach merits improvement so that delivered treatments are more efficient, effective, and personalised to individuals' concerns.

Existing psychological treatments for CMHP share more similarities than differences [21]. Despite differences in the theoretical foundation underlying available psychological treatments, and the terms used to describe maintaining factors and treatment targets, there are many common elements. For instance, psychoeducation, graded exposure, mindfulness techniques, and behavioural activation form a key component of a variety of effective treatments such as trauma-focused cognitive behavioural therapy, cognitive behavioural therapy (CBT), acceptance and commitment therapy (ACT), Dialectical Behaviour Therapy

For peer review only

(DBT), exposure therapy and mindfulness-based cognitive therapy (MBCT). The widespread availability of so many treatment options has the potential to elicit considerable decision-making difficulties for the treating clinician. In both formulation and treatment planning, challenging decisions occur when selecting the order in which to treat multiple difficulties, in evaluating the most appropriate treatment approach, and working out which treatment option will be acceptable and effective for the client.

A recent equivalence RCT demonstrated that a transdiagnostic protocol (The *Unified Protocol for Transdiagnostic Treatment of Emotional Disorders* [22]) and single-disorder protocols produced statistically equivalent reduction in severity of principal anxiety disorder diagnosis, but that there was less attrition in the transdiagnostic group [23]. Promising results have also been found for other transdiagnostic treatment protocols, including Norton's Transdiagnostic Group Cognitive Behavioral Therapy for Anxiety [15], Gros's Transdiagnostic Behavior Therapy for affective disorders [24], and Schmidt's False Safety Behaviour Elimination Therapy for Anxiety Disorders [25]. In addition, our systematic review and meta-analysis supported the overall efficacy of transdiagnostic treatments [10]. The review called for more high-quality studies to resolve uncertainties surrounding the heterogeneity of treatment effects and to determine the best treatment approaches and designs. We aim to address these issues through evaluating a novel intervention which combines a number of evidence-based treatment strategies. In utilising a modular approach, this trial will contribute to identification and evaluation of effective treatment components and delivery method. The modular approach to treatment design incorporates self-contained functional units (therapy modules) that can operate independently and be delivered flexibly, but also refer to other modules if needed [22]. A complex, modular, tailored transdiagnostic intervention that targets common underlying processes maximises goodness of fit and has a

For peer review only

direct focus on process rather than symptoms. The approach is thereby suitable for complexity and comorbidity as well as sub-syndromal and prodromal symptoms.

The transdiagnostic intervention we have developed – *Shaping Healthy Minds* – targets the processes and symptoms that are common to CMHPs and offers a number of advances in transdiagnostic treatment by incorporating the best available techniques from existing manual-based treatments into the one treatment package. A key aim of the programme is to encompass the treatment techniques that skilled psychologists and mental health clinicians already implement in standard practice for depressed, stressed and anxious patients with complex presentations [26]. The intervention also builds on the Unified Protocol for Transdiagnostic Treatment for Emotional Disorders described by Barlow et al. [14], by working towards a prescriptive approach for the delivery of treatment modules based on the formulation of the client's presenting difficulties [9]. Specifically, the treatment expands beyond interventions grounded in a sole treatment paradigm (e.g., CBT) towards a theory-driven approach that utilises efficacious techniques translated from basic science alongside components drawn from a wide range of evidence-based psychological treatments (e.g., Mindfulness-based interventions, ACT, Behavioural Activation, and DBT).

The treatment protocol for *Shaping Healthy Minds* also changes the way that standardised manual-based treatments are delivered. Rather than using integral interventions (where all patients receive the same relatively fixed, complete protocol), the transdiagnostic intervention is a modular intervention, whereby the assessment of core problematic areas of emotional, cognitive, interpersonal, and behavioural processes informs the selection and sequence of treatment modules targeted at specific problem areas for patients [22]. This modular approach allows for standardised, yet flexible treatment that is personalised to the individual concerns, problems, and goals for the patient. Finally, it expands beyond interventions that typically focus on alleviating negative symptomatology (e.g., negative

For peer review only

thoughts, excessive negative emotions) and incorporates interventions designed to increase positive emotions, capture strengths, and enhance resilience for sustained recovery.

We aim to examine the feasibility of *Shaping Healthy Minds* in reducing symptoms of depression, anxiety, distress, disability and functional impairment through an early-stage randomised clinical trial, in line with recommendations for the development of complex interventions [27]. In particular, we will gather data on the extent to which *Shaping Healthy Minds* performs comparably to TAU for a given service-user's primary diagnosed problem [10, 28] as well as for other significant additional, secondary, and/or comorbid difficulties. In addition, this trial will provide a preliminary evaluation of whether a modular transdiagnostic treatment approach may be effective at reducing the distress and impairment associated with CMHP [10]. The trial will also provide an indication of the feasibility and acceptability, of the transdiagnostic intervention to service-users and clinicians by recruiting through post-primary care UK NHS psychology services where complex comorbidity represents the modal clinical presentation. In addition, the trial will provide initial estimates of cost-effectiveness in terms of service-use and potential quality-adjusted life years added. We therefore present the protocol for a feasibility trial with co-primary outcomes, examining the effect of *Shaping Healthy Minds* on primary and comorbid diagnoses. The feasibility trial will provide a plausible range of point estimates of the efficacy of *Shaping Healthy Minds* on standardised continuous symptom measures for primary and secondary diagnoses to inform this key question, refine the treatment manual and contribute to the design of future scaled-up trials [27].

Methods and Analysis

Study Design

The design is a parallel arm RCT comparing *Shaping Healthy Minds* to TAU. Participants will be assessed three times - at baseline, at post-treatment, and at 3-month

For peer review only

follow-up. These three time points involve face-to-face assessments including the full battery of primary and additional outcomes and process measures, described below.

Participants and recruitment

The proposed feasibility study will seek to recruit 50 people aged 18 and above with a primary diagnosis of a unipolar mood, anxiety, obsessive-compulsive, or trauma- and stressor-related disorder (CMHPs) with at least one additional comorbid diagnosis according to the Diagnostic and Statistical Manual of Mental Disorders – Fifth Edition (DSM-5 [29]). Participants will be randomly allocated to one of two groups: (1) *Shaping Healthy Minds*, or (2) TAU. Diagnosis of CMHPs will be determined by trained research staff using the Structured Clinical Interview for the DSM-5 (SCID-5; [30]). To be eligible participants will also need to score > 10 on either the Patient Health Questionnaire (PHQ-9) or the Generalised Anxiety Disorder Questionnaire (GAD-7; see Study Measures below). Exclusion criteria are current/past psychosis or bipolar disorder, current diagnosis of alcohol or substance use disorder (all assessed via the Structured Clinical Interview for the DSM-5; SCID-5; [30]), organic brain damage, complex trauma history or recurrent self-injury requiring specialist services, or current suicidality that warrants immediate clinical attention and constitutes a current risk of harm to the individual (all assessed via participant report and the clinical care team). Participants may be engaged with the multi-disciplinary clinical care team, but those randomised to *Shaping Healthy Minds* will not be receiving other psychological services whilst participating in the trial. All other services (e.g., medication review with psychiatrist or general practitioner, occupational therapy, social support services) may be continued, and there are no medication exclusions.

Participants will be recruited through local NHS clinical psychology services, including the high intensity team of the Cambridge Psychological Wellbeing Service, and secondary care services with expertise in treatment of more complex and comorbid affective

For peer review only

disorders. The recruitment pathways will involve suitable service-users on a waitlist to receive treatment being identified by a member of the clinical service (including an Assistant Psychologist focusing primarily on recruitment into clinical research studies) who will provide them with a letter outlining the study. Service-users will then be able to contact the research team to opt into the study. Initial eligibility will be screened over the telephone, and suitable participants will be invited to complete the SCID, either at the clinical service or the research unit. At the beginning of this session, all participants will provide written informed consent (see supplementary materials for a sample Participant Information Sheet and Consent Form). No potential participants will be contacted by a member of the research team until they have given consent for such contact to a member of the clinical care team.

Participant allocation

Following both baseline assessment sessions, eligible participants will be stratified according to depression (PHQ-9) and anxiety (GAD-7) severity scores and randomised to either *Shaping Healthy Minds* (n = 25) or TAU (n = 25). This will be achieved using computer generated, quasi-random numbers and will be conducted by the trial statistician (PW), blind to study objectives. Once generated, this information is passed to the project coordinator responsible for delivering the intervention. Once a participant begins treatment, he or she is free to discontinue participation at any time, in which case s/he will be referred back to the appropriate NHS clinical care team. Figure 1 summarises participant flow through the trial.

Interventions

Shaping Healthy Minds (SHM) is a modular intervention, comprising 10 independent modules that will last up to 20 sessions. The content of the modules is drawn from a number of evidence-based psychological therapies, including CBT [31], ACT [32], DBT [33], MBCT [34], and behavioural activation [35, 36]. The programme aims to bring together the core and

For peer review only

unique therapeutic techniques from the best available disorder-focused treatment packages into the one transdiagnostic treatment package (e.g., behavioural experiments [37] and graded exposure [38] from CBT, values exercises and mindfulness strategies from ACT [39], activity scheduling from BA [35], emotion regulation strategies from DBT [33], and present-moment awareness exercises from MBCT [40]). The elements of *Shaping Healthy Minds* were drawn from recent meta-analyses supporting the effectiveness of these treatment strategies (e.g., [36, 41-43]), and the manuals were written and reviewed by experienced clinical psychologists (TD, JN, AB, CH, and MB). In addition, experts in particular fields (e.g., WK for mindfulness and case formulation) were consulted on the content of specific modules. Further, we received service-user input on the content of the draft modules, and a part of this feasibility trial will be receiving feedback from participants on their experience of using the manuals.

The modular approach is standardised, yet can be flexibly delivered according to an individual's concerns, problems, and goals [22]. The treatment focuses both on alleviating negative symptoms and enhancing positive wellbeing, by teaching skills and techniques and enhancing positive emotions, harness and build on strengths, and maximise resilience over the longer-term. Choice, order, and length of modules (i.e., number of session over which they are completed) is tailored to the transdiagnostic difficulties of the individual using collaborative case formulation [44], although there are three core modules that everyone receives (outlined below). Treatment consists of weekly face-to-face one-hour sessions with the trial therapists. Sessions will involve collaboratively setting an agenda for the session based on the participants' ratings for their top 3 problems and top 3 strengths, the goals set for therapy, and the module in focus. Participants will complete homework exercises to consolidate and practice the skills learned during the specific modules and will be strongly encouraged to continue this practice following the end of one module and move to the next.

For peer review only

The ten SHM modules (core modules listed in bold) are: (1) **Getting Acquainted with SHM**, (2) **Understanding Emotions**, (3) Managing and Tolerating Emotions, (4) Behavioural Activation, (5) Tackling Avoidance, (6) Tackling Unhelpful Thoughts, (7) Tackling Unhelpful Habits, (8) Overcoming Repetitive Thinking, (9) Managing Upsetting Memories and Images, and (10) **Relapse Prevention and Future Orientation**. Additional information about the content of the modules can be found in the supplementary materials.

Treatment-as-usual (TAU). For TAU, clinical psychologists and high-intensity CBT Therapists in teams specialising in CMHPs will be asked to provide the course of psychological therapy that they deem appropriate, in addition to referral to other health/social services and medication management. Psychological treatment in the specialist teams delivering TAU will standardly consist of disorder-focused Cognitive Behavioural Therapy, Eye Movement Desensitisation Reprocessing, or Behavioural Activation. The delivered treatment will be documented to ensure systematic understanding of the duration, frequency, and type of treatment administered.

Treatment Integrity. Therapists with experience in treating adult CMHPs will deliver the transdiagnostic intervention. Treatment fidelity and clinician adherence for the *Shaping Healthy Minds* group will be established using continued monitoring of completion of module components and through independent rating of specific treatment strategies by the supervising clinical psychologist. After every session, clinicians will complete a bespoke Treatment Fidelity Checklist which is a session-by-session self-report measure of compliance with the *Shaping Healthy Minds* approach, and these will be evaluated during weekly clinical supervision with the trial clinical supervisor. In addition, a randomly-selected 25% of the audio-taped treatment sessions will be rated for adherence to the manuals by an experienced clinician, independent of the trial. Homework completion will be monitored by trial therapists.

For peer review only

This bespoke measure will be supplemented with The Cognitive Therapy Scale – Revised (CTS-R) – a standardised measure of competence within cognitive therapy, consisting of adherence to and skilful application of cognitive therapy methods and the therapeutic alliance [45]. The CTS-R has 13-items that are completed by an independent rater, assessing agenda setting, feedback, collaboration, pacing and efficient use of time, interpersonal effectiveness, charisma/flair, facilitation of emotional expression, guided discovery, conceptualisation, identifying key cognitions, application of cognitive change methods, application of behavioural techniques, use of homework.

Measures

Co-primary outcomes. The co-primary outcome measures are self-reported symptoms of depression and anxiety, indexed by the PHQ-9 [46], and GAD-7 [47], as well as levels of disability and functional impairment, indexed by the Work and Social Adjustment Scale (WSAS [48]).

Secondary outcomes. Given the transdiagnostic focus of the study, self-reported symptoms on specific disorders that client's meet criteria for at trial baseline will be indexed by the IAPT Phobia Scales (social phobia, agoraphobia, specific phobia, [49]), the Social Phobia Inventory (SPIN, [50]), the Penn State Worry Questionnaire (generalised anxiety, PSWQ, [51]), the Obsessive-Compulsive Inventory (OCI, [52]), the Revised Impact of Event Scale (posttraumatic stress, IES-R, [53]), the Agoraphobia-Mobility Inventory (MI, [54]), the Fear Questionnaire (specific phobias, FQ, [55]), the Panic Disorder Severity Scale- self report version (PDSS-SR, [56]), the Health Anxiety Inventory – short version (SHAI, [57]), and the Sheehan Disability Scale (SDS, [58]). Participants will only complete a selection of these measures depending on their concerns and associated diagnoses. In addition to these disorder-specific measures, the Inventory of Depression and Anxiety Symptoms (IDAS-II) will capture both disorder-specific and transdiagnostic symptom dimensionality within a single measure

For peer review only

[59]. Selection of these measures will be determined following completion of the structured clinical interview at the beginning of assessment.

Process Measures. We will also include a number of process-related measures which will be administered at baseline, at post-intervention, and at 3-month follow-up to begin to explore mechanisms of change and the feasibility of conducting embedded process outcome research within this type of trial (see Table 1). To explore the value of the individual modules administered within the transdiagnostic intervention, we will also administer module-relevant measures (e.g., rumination, distress tolerance) before and after completion of the module. Finally, participants' expectancy of treatment outcomes and measures of engagement and compliance will be administered at pre and post-intervention to inform the further development of the protocol.

Health economics measures. Data collection for the health economic evaluation will take a patient-level perspective [74, 75], recording the cost-per-session of treatment and productivity losses resulting from time off work as a consequence of their mental health difficulties. Data will be collected using the Healthlines Resource Use Questionnaire [74, 75], which is a measure of the participants use of health care services (including NHS, help at home), occupational productivity (i.e., time off work) and cost of transdiagnostic treatment delivery (e.g., direct and indirect time spent in service delivery). The Medical Outcomes Study Questionnaire Short Form 36 Health Survey (SF-36) [76], a generic quality of life questionnaire, will measure overall health and wellbeing, daily functioning, and general life satisfaction across multiple domains. These measures allow calculation of the additional number of quality of life years the treatment will yield. These data will allow preliminary estimates of the potential cost-utility of the transdiagnostic intervention and also of the feasibility of acquiring these data within the trial protocol.

For peer review only

Methodological Aspects

Power analysis and sample size. Although a standard power calculation based on detecting treatment effects is the conventional approach to determining sample sizes for trials, the main aim of the current trial is to elucidate feasibility for a larger later-stage evaluation. We therefore sought at this stage only to provide a point-estimate of the effect of *Shaping Healthy Minds* to inform a power calculation for this putative fully powered later phase evaluation. Our previous experience with such early phase trial platforms indicates that 50 patients will provide sufficient numbers and diagnostic diversity to evaluate feasibility, acceptability, and procedural uncertainty for *Shaping Healthy Minds* and a plausible test of recruitment protocols. This will give 40 patients (20 per arm) at 3-month posttreatment follow up, assuming 20% attrition. This will provide a reasonable range of point estimates of effect on our set of candidate outcome measures sufficient to guide later phase trial work.

Data collection and confidentiality. Outcome data for all participants who are randomised will be collected via face-to-face interviews and written questionnaires at baseline, post-treatment, and 3-month follow-up. To maintain confidentiality all participants will be given a trial number so that personally identifying information is not linked to assessment or trial information. All data (including personally-identifiable information) will be stored on secure UK NHS databases, secure University of Cambridge servers and within locked filing cabinets under the management of the trial coordinator. Access will be limited to the immediate clinical research team.

Blinding. Outcome assessments will be conducted by independent raters who have no therapeutic relationship with the patients and are blind to treatment condition. Double blinding of patients and therapists is not possible due to the nature of the trial (i.e., a psychological intervention). Unblinding will not be necessary because participants and therapists are not blinded to intervention allocation.

For peer review only

Statistical analysis plan. Initial analyses of the outcomes will be conducted by the trial statistician, blind to trial condition, following CONSORT standards (see Figure 2, supplementary materials). There are no planned interim analyses. Initial analyses will be conducted on an intention-to-treat basis, with subsequent analyses being per protocol. Mixed model analyses of variance (ANOVAs) will be used to compare groups on outcomes at the three assessment points – baseline, post-intervention, and 3-month follow-up. Baseline levels on relevant measures will be included as covariates, as appropriate. Both intent-to-treat and per-protocol exploratory analyses will be conducted with our range of outcome measures following CONSORT standards. Multiple imputation will be used to account for missing data. Intent-to-treat analysis will also be used for those lost to attrition. Exploratory moderation and mediation analyses to examine process variables will be conducted using the MacArthur approach [77]. For the health economic data, costs associated with service use will be calculated by attaching a unit cost to each instance of use, and data will be combined with quality-adjusted life years (QALYs, [72]) derived from the SF-36 to arrive at a preliminary estimate of the cost-utility of the transdiagnostic intervention.

Monitoring and data management. The trial will take place at NHS sites and a research unit in the East of England. A Trial Management Group (TMG), will meet 1-2 times a year to: manage the protocol; monitor recruitment in relation to targets; deal with any adverse events; and co-ordinate the different stages of the project. The TMG consists of research clinical psychologists and assistant psychologists, a psychiatrist, a health economist, clinical psychology researchers, the trial statistician, a nurse practitioner, and a service user representative. Day-to-day project management will be the responsibility of a smaller trial team, meeting fortnightly to deal with administrative issues, troubleshooting, and recruitment flow. Clinical supervision will take place fortnightly. As this is a Phase I/II trial, a data-management committee was deemed unnecessary, and as such the trial team are responsible

For peer review only

for monitoring and data management. Data will be monitored for completeness and consistency using spot checks and plausibility checks carried out by the trial statistician. The trial lead, trial coordinator, and statistician will have full access to the final trial dataset. The study data will be reported in line with the current CONSORT recommendations.

Safety aspects. Adverse events are managed in line with UK MRC protocols and, in the unlikely case of an adverse event, will be documented appropriately. Precautions have been taken to reduce the likelihood of adverse events occurring; for example, patients who are acutely suicidal or at high risk of harm do not meet study inclusion criteria. The interventions are delivered by therapists experienced in the management of risk and in the treatment of psychological disorders. In the case of any adverse events as a result of the intervention that would interfere with participation, participation in the trial will be discontinued. Regular team meetings will be conducted to monitor any difficulties patients may be having and ways of best dealing with these difficulties. Serious adverse events will be reported to the Ethics Committee. The trial is underwritten by the University of Cambridge in the case that any individual suffers harm or requires post-trial care. Any adverse events will be reported in the trial paper.

Ethical approval and protocol amendments. This project has received ethical approval from the Health Research Authority of the UK National Health Service (East of England; REC reference: 16/EE/0095). The study will be conducted within appropriate UK MRC, National Health Service and professional ethical guidelines, ensuring that Good Clinical Practice procedures are adhered to at all times. Protocol amendments will be circulated to the ethics committee, and trial team, and published in the online registration of the trial, and in the trial paper.

Dissemination policy. There are no publication restrictions and findings will be disseminated broadly to participants, healthcare professionals, the public, and other relevant

For peer review only

groups. Academic outputs will take the form of peer-reviewed empirical journal articles, commentary pieces, and conference presentations. Clinical outputs will be prioritised by the research team in order to maximise the impact of the findings with practitioners and commissioners. Outputs will comprise clinical conferences, workshops, service user groups, and a study website that will make the intervention materials and related measures generally available. We will send all participants a report describing the findings and their implications. We will also make participants aware of the study website. We anticipate that trial findings will inform future revisions of clinical guidelines for numerous forms of mood, anxiety, and stress disorders, and the development of guidelines for comorbid conditions. Anonymised data from the trial will be made publicly available on an open-access database.

Discussion

A significant proportion of the cost of CMHPs is generated by adults suffering from complex and comorbid depression, stress and anxiety, where treatment non-response, cross-sector service-use across health, social care, and housing, and loss of productivity are greatest. Providing effective interventions for these mental health problems therefore has the potential to reduce both long-term treatment costs as well as prevent large productivity losses. At present, most psychological interventions focus on specific diagnoses and many treatment manuals take a 'one-size-fits-all' approach. Current evidence-based interventions only achieve clinical recovery for 40-70% of patients, with people suffering complex co-morbid conditions faring significantly worse. This randomised controlled feasibility trial aims to pave the way for a scaled-up efficacy trial of a new transdiagnostic modular treatment for all CMHPs – *Shaping Healthy Minds* – that enables the flexible delivery of evidence-based techniques. This treatment approach may improve the effectiveness and dissemination of evidence-based intervention for the many individuals for whom diagnosis-specific treatments leave significant difficulties unaddressed. The results from this trial will provide a range of

For peer review only

estimates of effect sizes that can be used to power a later stage trial of treatment efficacy, to refine the treatment protocol, and to inform future evaluation of the mechanisms underlying any treatment effects. If effective, *Shaping Healthy Minds* has the potential to improve outcome for those with complex presentations, through offering a cost-effective treatment option to reduce chronic, transdiagnostic psychological difficulties.

Trial Status: This trial was registered at clinicaltrials.gov on 4 May 2017 (NCT03143634). This article was submitted on 31 May 2018. To date, 15 participants have met eligibility criteria for the study and have been randomised to a condition. The trial opened on 31 July 2017, and data collection aims to be completed by September 2019.

For peer review only

List of Abbreviations

DSM – Diagnostic and Statistical manual

IAPT – Improving Access to Psychological Therapies

HARMONIC – The Healthy and Resilient Mind Programme: Building Block for Mental Wellbeing

MRC – Medical Research Council

NHS – National Health Service

PTSD – Posttraumatic Stress Disorder

RCT – Randomised Controlled Trial

SCID-5 – Structured Clinical Interview for DSM-5 disorders

TAU – Treatment-as-usual

CMHP – Common Mental Health Problems

NICE – National Institute for Health and Care Excellence

CBT – Cognitive Behaviour Therapy

ACT – Acceptance and Commitment Therapy

DBT – Dialectical Behaviour Therapy

MBCT – Mindfulness Based Cognitive Therapy

For peer review only

Authors' contributions

MB will manage the trial, deliver the intervention, co-develop the treatment manuals and helped to draft the manuscript; CH will assist with trial management and helped to draft the manuscript; AB advises on the delivery of the interventions and provided guidance on the protocol; JC provides practical and NHS service support for the trial; COL, RE and DJ provides support for the trial management; PW is the trial statistician responsible for the randomisation, advise on analysis strategy, and analysis of the data; LL provides support for the health economics component; SR provides service-user support for the trial; SG and WK advised on the treatment manual; JN co-developed and adapted the treatment manuals; TD designed the study, co-developed and adapted the treatment manuals, and helped to draft the manuscript. All authors read and approved the final manuscript.

Authors' information

Melissa Black:	melissa.black@mrc-cbu.cam.ac.uk
Caitlin Hitchcock:	caitlin.hitchcock@mrc-cbu.cam.ac.uk
Anna Bevan:	anna.bevan@mrc-cbu.cam.ac.uk
Cliodhna O Leary:	cliodhna.oleary@cpft.nhs.uk
James Clarke:	james.clarke@cpft.nhs.uk
Rachel Elliott:	rachel.elliott@cpft.nhs.uk
Peter Watson:	peter.watson@mrc-cbu.cam.ac.uk
Louise Lafortune:	ll394@medschl.cam.ac.uk
Sarah Rae:	sarah.rae@rockyroad.org.uk
Simon Gilbody:	simon.gilbody@york.ac.uk
Willem Kuyken:	willem.kuyken@psych.ox.ac.uk
David Johnston:	david.johnston@mrc-cbu.cam.ac.uk
Jill Newby:	j.newby@unsw.edu.au
Tim Dalgleish:	tim.dalgleish@mrc-cbu.cam.ac.uk

For peer review only

Funding

This paper presents independent research funded by the National Institute of Health Research (NIHR) under its Research for Patient Benefit (RfPB) Programme (Grant Reference Number PB-PG-0214-33072) grant awarded to Tim Dalgleish, Peter Watson, Jill Newby, Leonora Brosan, Rajini Ramana, Louise Lafortune, Sarah Rae, Simon Gilbody, Willem Kuyken, and Caitlin Hitchcock. Jill Newby is supported by a NHMRC/MRFF Fellowship (1145382). Please note that the funding body does not have authority over the running of the trial – all decisions rest with the trial team. The views expressed are those of the author(s) and not necessarily those of the NHS, the NIHR or the Department of Health and Social Care.

Competing Interests

The authors have no competing interests.

Patient consent

Obtained.

Ethics approval

NHS National Research Ethics Committee (East of England, Reference: 16/EE/0095)

Sponsor Information

University of Cambridge School of Clinical Medicine

Carolyn Read (Research Governance Officer)

Cad50@medschl.cam.ac.uk

Box 111 Cambridge Biomedical Campus

Cambridge, CB2 0SP

United Kingdom

For peer review only

References

1. Kessler, R.C., et al., The epidemiology of major depressive disorder: results from the National comorbidity survey replication (NCS-R). Journal of the American Medical Association, 2003. 289: p. 3095e3105.
2. Medical Research Council, Review of mental health research: Report of the strategic review group. 2010, Medical Research Council: London.
3. Pilling, S., et al., Identification and care pathways for common mental health disorders: summary of NICE guidance. BMJ, 2011. 342: p. d2868.
4. Brown, T.A., et al., Current and lifetime comorbidity of the DSM-IV anxiety and mood disorders in a large clinical sample. Journal of Abnormal Psychology, 2001. 110(4): p. 585-599.
5. Kessler, R.C., et al., Lifetime prevalence and age-of-onset distributions of DSM-IV disorders in the national comorbidity survey replication. . Archives of General Psychiatry, 2005. 62(6): p. 593-602.
6. Moses, E.B. and D.H. Barlow, A new unified treatment approach for emotional disorders based on emotion science. Current Directions and Psychological Science, 2006. 15(3): p. 146-150.
7. Bockting, C.L., et al., A lifetime approach to major depressive disorder: The contributions of psychological interventions in preventing relapse and recurrence. Clinical Psychology Review, 2015. 41: p. 16-26.
8. Loerinc, A.G., et al., Response rates for CBT for anxiety disorders: Need for standardized criteria. Clinical Psychology Review, 2015. 42: p. 72-82.
9. Ellard, K.K., et al., Unified Protocol for the Transdiagnostic Treatment of Emotional Disorders: Protocol Development and Initial Outcome Data. Cognitive and Behavioral Practice, 2010. 17: p. 88-101.
10. Newby, J.M., et al., Systematic review and meta-analysis of transdiagnostic psychological treatments for anxiety and depressive disorders in adulthood. Clin Psychol Rev, 2015. 40: p. 91-110.
11. Craske, M.G., Transdiagnostic Treatment for Anxiety and Depression. Depression and Anxiety, 2012. 29: p. 749-753.
12. Brown, T.A. and D.H. Barlow, A proposal for a dimensional classification system based on the shared features of the DSM-IV anxiety and mood disorders: implications for assessment and treatment. Psychol Assess, 2009. 21(3): p. 256-71.
13. Harvey, A., G., et al., eds. Cognitive behavioural processes across psychological disorders: A transdiagnostic approach to research and treatment. 2004, Oxford University Press: Oxford.
14. Barlow, D.H., L.B. Allen, and M.L. Choate, Toward a Unified Treatment for Emotional Disorders. Behavior Therapy, 2004. 35: p. 205-230.
15. Norton, P.J., S.A. Hayes, and D.A. Hope, Effects of a transdiagnostic group treatment for anxiety on secondary depression. Depress Anxiety, 2004. 20(4): p. 198-202.
16. Richards, D.A., et al., Cost and Outcome of Behavioural Activation versus Cognitive Behavioural Therapy for Depression (COBRA): a randomised, controlled, non-inferiority trial. The Lancet, 2016. 388(10047): p. 871-880.

17. Borkovec, T.D. and E. Costello, Efficacy of applied relaxation and cognitive-behavioral therapy in the treatment of Generalised Anxiety Disorder. Journal of Consulting and Clinical Psychology, 1993. 61: p. 611-619.
18. Dugas, M.J., et al., Group Cognitive–Behavioral Therapy for Generalized Anxiety Disorder: Treatment Outcome and Long-Term Follow-Up. Journal of Consulting and Clinical Psychology, 2003. 71: p. 821-825.
19. Clark, D.M., et al., Cognitive therapy versus exposure and applied relaxation in social phobia: A randomized controlled trial. J Consult Clin Psychol, 2006. 74(3): p. 568-78.
20. Bryant, R.A., et al., Imaginal exposure alone and imaginal exposure with cognitive restructuring in treatment of posttraumatic stress disorder. Journal of Consulting and Clinical Psychology, 2003. 71(4): p. 706-712.
21. Chorpita, B.F. and E.L. Daleiden, Mapping evidence-based treatments for children and adolescents: application of the distillation and matching model to 615 treatments from 322 randomized trials. Journal of Consulting and Clinical Psychology, 2009. 77(3): p. 566-79.
22. Chorpita, B.F., E.L. Daleiden, and J.R. Weisz, Modularity in the design and application of therapeutic interventions. Applied and Preventive Psychology, 2005. 11(3): p. 141-156.
23. Barlow, D.H., et al., The Unified Protocol for Transdiagnostic Treatment of Emotional Disorders Compared With Diagnosis-Specific Protocols for Anxiety Disorders: A Randomized Clinical Trial. JAMA Psychiatry, 2017.
24. Gros. D.F. Development and initial evaluation of Transdiagnostic Behavior Therapy (TBT) for veterans with affective disorders. Psychiatry Research, 2014, 220, 275-282.
25. Riccardi, C.J., Korte, K.J. and Schmidt, N.B. False Safety Behavior Elimination Therapy: A randomized study of a brief individual transdiagnostic treatment for anxiety disorders. Journal of Anxiety Disorders, 46, 35-45.
26. Wilson, G.T., Manual-based treatments: The clinical application of research findings. Behaviour Research and Therapy, 1996. 34(4): p. 295-314.
27. Craig, P., et al., Developing and evaluating complex interventions: The new Medical Research Council guidance. British Medical Journal, 2008. 337.
28. Reinholt, N. and J. Krogh, Efficacy of transdiagnostic cognitive behaviour therapy for anxiety disorders: a systematic review and meta-analysis of published outcome studies. Cognitive Behaviour Therapy 2014. 43: p. 171-184.
29. American Psychiatric Association. Diagnostic and Statistical Manual of Mental Disorders. Fifth ed. 2013, Arlington, VA: American Psychiatric Publishing.
30. First, M.B., et al., Structured Clinical Interview for DSM-5 - research Version (SCID-5 for DSM-5, Research Version, SCID5-RV, Version 1.0.0). 2015, Arlington, VA: American Psychiatric Association.
31. Beck, J., Cognitive Behavior Therapy: Basics and Beyond (Second Edition). 2011, New York: Guilford Press.
32. Hayes, S.C., K.D. Strosahl, and K.G. Wilson, Acceptance and commitment therapy: An experiential approach to behavior change. . 1999, New York: Guilford Press.
33. Linehan, M.M., Cognitive-Behavioral Treatment of Borderline Personality Disorder. 1993, New York: Guilford Press.
34. Segal, Z.V., J.M.G. Williams, and J.D. Teasdale, Mindfulness-based Cognitive Therapy for Depression: A new approach to preventing relapse. 2002, New York: Guilford Press.

35. Martell, C.R., S. Dimidjian, and H.-D. Ruth, Behavioral Acitvation for Depression: A Clinician's Guide. 2010, New York: Guilford Press.
36. Ekers, D., et al., Behavioural Activation for Depression; An update of meta-analysis of effectiveness and subgroup analysis. PLoS ONE, 2014. 6(6).
37. Bennett-Levy, J., et al., Oxford Guide to Behavioural Experiments in Cognitive Therapy. 2004, Oxford: Oxford University Press.
38. Norton, P.J., Group Cognitive-Behavioural Therapy of Anxiety. 2012, New York: Guilford Press.
39. Harris, R., ACT Made Simple: A quick start guide to ACT basics and beyond. 2009, Oakland, CA: New Harbinger Publications, Inc.
40. Kuyken, W., et al., How does mindfulness-based cognitive therapy work? Behaviour Research and Therapy, 2010. 48(11): p. 1105-1112.
41. Khoury, B., et al., Mindfulness-based therapy: A comprehensive meta-analysis. Clinical Psychology Review, 2013. 33(6): p. 763-771.
42. Dimidjian, S., et al., Considering Meta-Analysis, Meaning, and Metaphor: A Systematic Review and Critical Examination of "Third Wave" Cognitive and Behavioral Therapies. Behavior Therapy, 2016. 47: p. 886-905.
43. Cuijpers, P., et al., How effective are cognitive behavior therapies for major depressionand anxiety disorders? A meta-analytic update of the evidence. World Psychiatry, 2016. 15: p. 245-258.
44. Kuyken, W., C.A. Padesky, and R. Dudley, Collaborative Case Conceptualisation: Working Effectively with Clients in Cognitive-Behavioral Therapy. 2009, New York, NY. : The Guilford Press.
45. Blackburn, I.-M., et al., The Revised Cognitive Therapy Scale (Cts-R): Psychometric Properties. Behavioural and Cognitive Psychotherapy, 2001. 29(04).
46. Kroenke, K., R.L. Spitzer, and J.B.W. Williams, The PHQ-9: Validity of a Brief Depression Severity Measure. Journal of General Internal Medicine, 2001. 16(9): p. 6060-613.
47. Spitzer, R.L., et al., A brief measure for assessing generalized anxiety disorder: The GAD-7. Archives of Internal Medicine, 2006. 166: p. 1092-1097.
48. Mundt, J.C., et al., The Work and Social Adjustment Scale: a simple measure of impairment in functioning. British Journal of Psychiatry, 2002. 180(5): p. 461-464.
49. IAPT, D.o.H., The IAPT data handbook version 2.0.1. 2011: Published to DH Website, in electronic PDF format only. .
50. Connor, K.M., et al., Psychometric properties of the Social Phobia Inventory (SPIN): New self-rating scale. British Journal of Psychiatry, 2000. 176: p. 379-386.
51. Meyer, T.J., et al., Development and validation of the Penn State Worry Questionnaire. Behaviour Research and Therapy 1990. 28(6): p. 487-495.
52. Foa, E.B., et al., The validation of a new obsessive-compulsive disorder scale: The Obsessive-Compulsive Inventory. Psychological Assessment, 1998. 10(3): p. 206-214.
53. Weiss, D.S., The Impact of Event Scale: Revised, in Cross-cultural assessment of psychological trauma and PTSD, J.P. Wilson and C.S. Tang, Editors. 2007, Springer: New York. p. 219-238.
54. Chambless, D.L., et al., The Mobility Inventory for Agoraphobia. Behaviour Research and Therapy, 1985. 23(1): p. 35-44.

For peer review only - http://bmjopen.bmj.com/site/about/guidelines.xhtml

55. Marks, I.M. and A.M. Mathews, Brief standard self-rating for phobic patients. Behavior Research and Therapy, 1979. 17: p. 263-267.
56. Shear, M.K., et al., Multicenter collaborative panic disorder severity scale. American Journal of Psychiatry, 1997. 154(11): p. 1571-1575.
57. Salkovskis, P.M., et al., The Health Anxiety Inventory: development and validation of scales for the measurement of health anxiety and hypochondriasis. Psychological Medicine, 2002. 32(5): p. 843-853.
58. Sheehan, D.V., The Anxiety Disease. 1983, New York: Charles Scribner and Sons.
59. Watson, D., et al., Development and Validation of New Anxiety and Bipolar Symptom Scales for an expanded version of the IDAS (the IDAS-II). Assessment, 2012. 19(4): p. 399-420.
60. Devilly, G.J. and T.D. Borkovec, Psychometric properties of the credibility/expectancy questionnaire. Journal of Behavior Therapy and Experimental Psychiatry, 2000. 31(2): p. 73-86.
61. Garnefski, N., V. Kraaij, and P. Spinhoven, Negative life events, cognitive emotion regulation and emotional problems. Personality and Individual Differences, 2001. 30: p. 1311-1327.
62. Fresco, D.M., et al., Initial psychometric properties of the Experiences Questionnaire: Validation of a self-report measure of decentering. Behavior Therapy, 2007. 38: p. 234-246.
63. Izard, C.E., et al., Stability of emotion experiences and their relations to traits of personality. Journal of Personality and Social Psychology, 1993. 64(5): p. 847-860.
64. Morey, L.C., Development and initial evaluation of a self-report form of the DSM-5 Level of Personality Functioning Scale. Psychological Assessment, 2017. 29(10): p. 1302-1308.
65. Treynor, W., R. Gonzalez, and S. Nolen-Hoeksema, Rumination Reconsidered: A Psychometric Analysis. Cognitive Therapy and Research 2003. 27(3): p. 247-259.
66. Simons, J.S. and R.M. Gaher, The Distress Tolerance Scale: Development and Validation of a Self-Report Measure. Motivation and Emotion, 2005. 29(2): p. 83-102.
67. Gratz, K.L. and L. Roemer, Multidimensional Assessment of Emotion Regulation and Dysregulation: Development, Factor Structure, and Initial Validation of the Difficulties in Emotion Regulation Scale. Journal of Psychopathology and Behavioral Assessment, 2004. 26(1): p. 41-54.
68. Weissman, A.N., The Dysfunctional Attitude Scale: A Validation Study. 1979, University of Pennsylvania.
69. Baer, R.A., G.T. Smith, and K.B. Allen, Assessment of mindfulness by self-report: the Kentucky inventory of mindfulness skills. Assessment, 2004. 11(3): p. 191-206.
70. Reiss, S., et al., Anxiety sensitivity, anxiety frequency and the prediction of fearfulness. Behaviour Research and Therapy, 1986. 24(1): p. 1-8.
71. Foa, E.B., et al., The Posttraumatic Cognitions Inventory (PTCI): Development and validation. Psychological Assessment, 1999. 11(3): p. 303-314.
72. Jarrett, R.B., et al., Skills of Cognitive Therapy (SoCT): A New Measure of Patients' Comprehension and Use. Psychological Assessment, 2011. 23(3): p. 578-586.
73. Gámez, W., et al., Development of a measure of experiential avoidance: the Multidimensional Experiential Avoidance Questionnaire. Psychological Assessment, 2011. 23(3): p. 692-713.

74. Dixon, P., et al., Cost-effectiveness of telehealth for patients with depression: evidence from the Healthlines randomised controlled trial. British Journal of Psychiatry Open, 2016. 1: p. 262-269.
75. Salisbury, C., et al., Effectiveness of an integrated telehealth service for patients with depression: a pragmatic randomised controlled trial of a complex intervention. Lancet Psychiatry, 2016. 3: p. 515-525.
76. Ware, J.E. and C.D. Sherbourne, The MOS 36-item short-form health survey (SF-36). I. Conceptual framework and item selection. Medical Care, 1992. 30(6): p. 473-483.
77. Kraemer, H.C., et al., How and Why Criteria Defining Moderators and Mediators Differ Between the Baron & Kenny and MacArthur Approaches. Health Psychology, 2008. 27: p. S101-S108.

For peer review only

Table 1.

Additional Outcome and Process measures to assess changes in potential mechanisms of psychological distress and in response to specific transdiagnostic intervention modules.

Measure	Focus area
The Treatment Credibility / Expectancy Questionnaire (CEQ) [60]	Expectancy about treatment outcome, as well as the credibility of the treatment.
Cognitive Emotion Regulation Questionnaire (CERQ) [61]	Ability to contextualize negative events within a wider frame of reference.
The Experiences Questionnaire (EQ) [62]	Ability to disengage from troublesome mental content and take a more accepting stance towards it, as well as the tendency to engage in rumination.
Differential Emotions Scale (DES [63])	Intensity with which they experience different emotions on a typical day to obtain summary scores for positive emotions, negative emotions, and denied emotions (the number of emotions *not* endorsed by the participant).
Levels of Personality Functioning Scale (LPFS) [64]	Personality functioning based on the DSM-5 Alternative Model of Personality Disorders. It has four subscales: Identity, Self-Direction, Empathy, and Intimacy.
Ruminative Responses Scale of the Response Styles Questionnaire (RRS) [65]	Rumination (Module 7 – Overcoming Repetitive Thinking)
Distress Tolerance Scale (DTS)[66]	Ability to tolerate distress (Module 3 – Managing and Tolerating Emotions)
Difficulties with Emotion Regulation Scale (DERS) [67]	Ability to label, perceive, and regulate emotions (Modules 2 – Understanding Emotions and 3 – Managing and Tolerating Emotions)
Dysfunctional Attitudes Scale (DAS) short form (version 1 and 2) [68]	Negative beliefs, thoughts and assumptions (Module 6 – Tackling Unhelpful Thoughts)

For peer review only

Kentucky Inventory of Mindfulness Skills (KIMS) [69]	Mindful awareness (Module 2 – Understanding Emotions)
Anxiety Sensitivity Index (ASI) [70]	Fear of physical anxiety sensations (Module 3 – Managing and Tolerating Emotions and Module 5 – Tackling Avoidance)
Posttraumatic cognitions inventory – short version (PTCI) [71]	Trauma-related beliefs and maladaptive appraisals of intrusive symptoms (Module 9 – Managing Upsetting Memories and Images)
Skills of Cognitive Therapy (SoCT) [72]	Implementation of cognitive therapy skills (Module 6 – Tackling Unhelpful Thoughts)
The Multidimensional Experiential Avoidance Questionnaire [73]	Avoidance of internal experiences including thoughts, feelings, physical sensations (Module 5 – Tackling Avoidance)

For peer review only

HARMONIC Trial

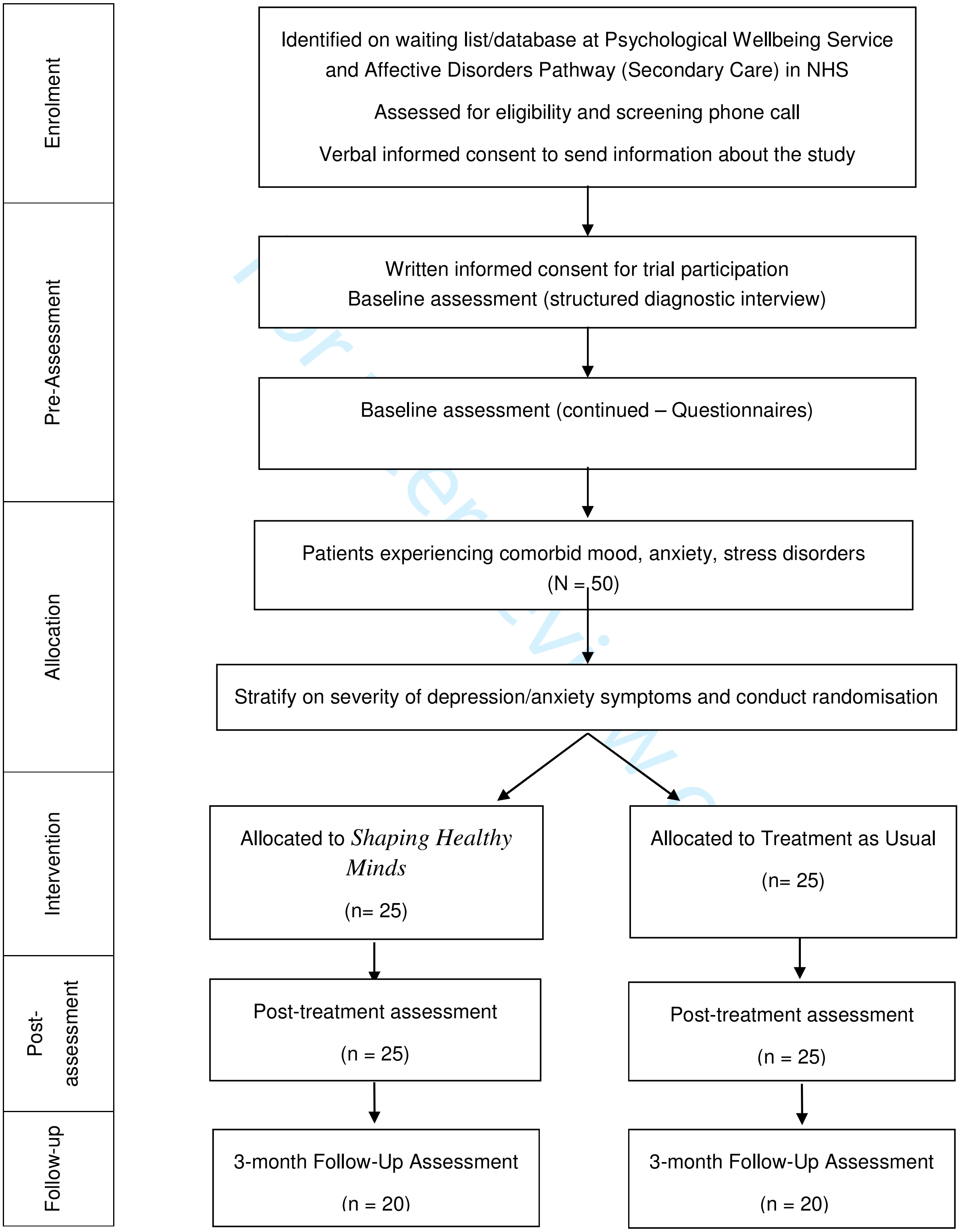

Figure 1. Participant flow diagram with anticipated participant numbers at each stage

HARMONIC Trial (Healthy and Resilient Mind Programme: Building Blocks for Mental Wellbeing)

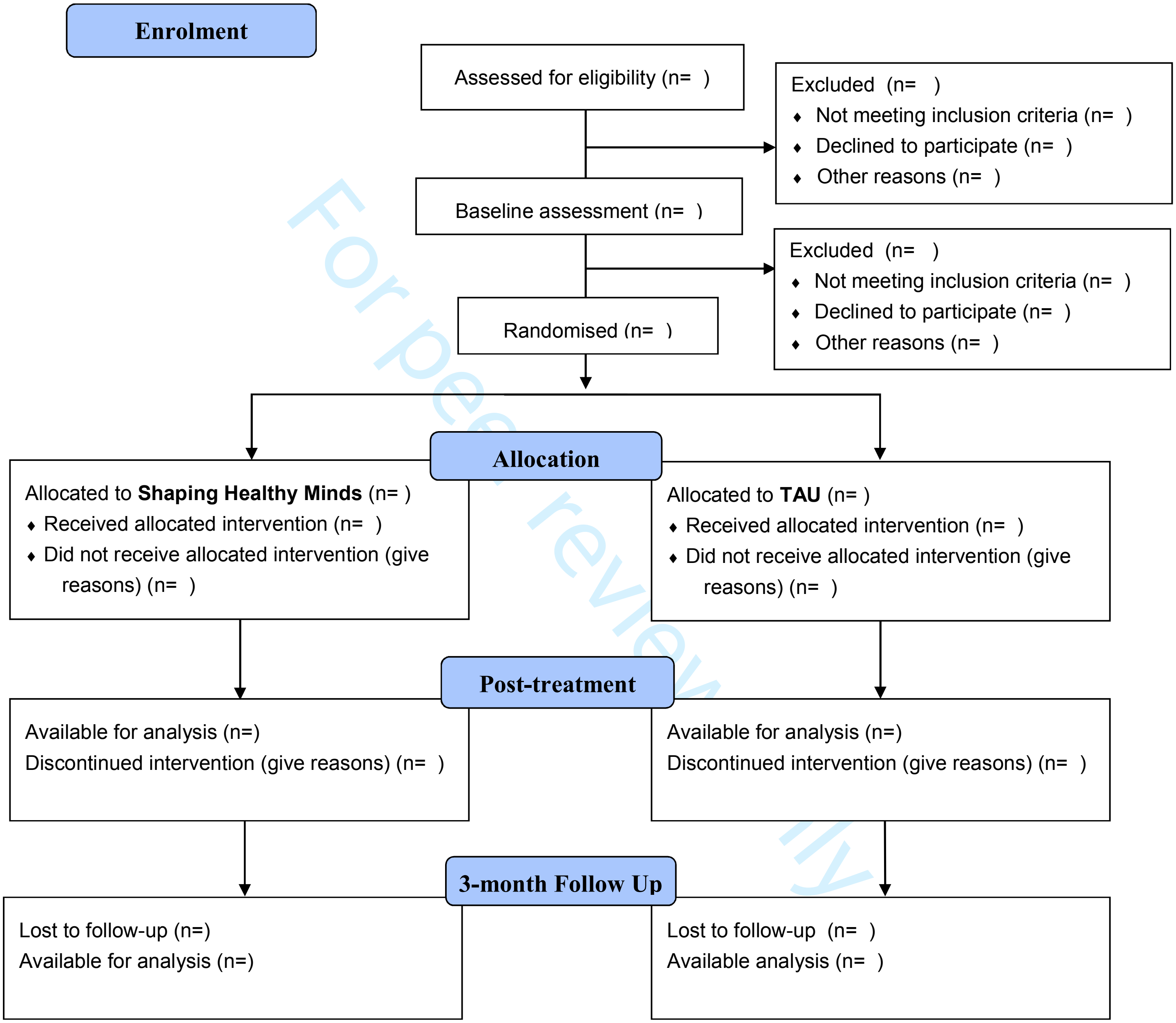

Figure 2. CONsolidated Standards of Reporting Trials (CONSORT) diagram

For peer review only

Table 1.

Key treatment components of Shaping Healthy Minds Core and Optional Modules.

#	Module Title	Sections of Module	Duration (sessions)
1	Getting Acquainted with *Shaping Healthy Minds* *Core Module*	• Education about depression and anxiety, and about emotions (Including 5-part model of emotion episodes)<br>• Orientation to treatment<br>• Defining Top 3 Problems and Top 3 Strengths<br>• Setting treatment goals and making plans<br>• Enhancing motivation	1-3
2	Understanding emotions *Core Module*	• Education about emotions<br>• Orientation to the emotion model<br>• Self-monitoring of emotion episodes<br>• Introduction to Mindful Awareness of Emotions	1-2
3	Managing and Tolerating Emotions *Optional Module*	• Education about emotion management toolbox<br>• Relaxation<br>• Exercise<br>• Social Support<br>• Distraction<br>• Self-soothing<br>• Accepting and Tolerating Feelings	1-3
4	Behavioural Activation *Optional Module*	• Education about low activity cycle<br>• Activity Monitoring<br>• Activity Scheduling and establishing positive routines<br>• Savouring the good things	1-2
5	Tackling Avoidance *Optional Module*	• Education about avoidance<br>• In vivo exposure<br>• Interoceptive exposure<br>• Emotion exposures	1-3
6	Tackling unhelpful thoughts *Optional Module*	• Education about thoughts<br>• Thought monitoring<br>• Thought questioning and cognitive flexibility<br>• Behavioural experiments<br>• Accepting and tolerating thoughts	1-3
7	Tackling unhelpful habits	• Education about habits<br>• Self-monitoring of habits	1-3

For peer review only

	Optional Module	• Step-by-step habit change process	
8	Overcoming Repetitive Thinking *Optional Module*	• Education about rumination and worry<br>• Self-monitoring of rumination and worry<br>• Getting unstuck toolbox<br>• Shifting thinking styles and perspective, directing attention	1-4
9	Managing upsetting memories and images *Optional Module*	• Education about memories and intrusive images<br>• Monitoring of memories<br>• Imaginal exposure<br>• Rescripting memories<br>• Reliving + rescripting memories	2-4
10	Preventing relapse and building a positive future *Core Module*	• Education about lapses and relapses<br>• Evaluating treatment progress and consolidating learning<br>• Creating a therapy blueprint (relapse prevention plan)<br>• Setting goals and making future plans	1

For peer review only

CAMBRIDGE CLINICAL RESEARCH CENTRE FOR AFFECTIVE DISORDERS

Cambridgeshire and Peterborough NHS Foundation Trust

MRC Cognition and Brain Sciences Unit

Participant Information Sheet
Study title: *Shaping Healthy Minds*

We invite you to participate in a study investigating a new modular treatment for mental health difficulties. Please read this information sheet if you wish to hear about the study in more detail. Your participation is *entirely voluntary*. Your treatment through the NHS will not be affected if you decide not to participate in this research.

This treatment – *Shaping Healthy Minds* (SHM) – treats symptoms that are common across depression and multiple anxiety disorders. The treatment consists of components of the best available evidence-based psychological treatments (e.g., mindfulness-based interventions, Acceptance and Commitment Therapy, Cognitive Behavioural Therapy, Behavioural Activation, and Dialectical Behaviour Therapy). Through using components of different types of therapy, the SHM aims to not only treat the principle problem that each individual is experiencing (e.g., depression or anxiety) but to target other psychological symptoms the individual is experiencing at the same time. In this way, someone who is experiencing both depression and anxiety will receive treatment for both of these issues, in contrast to the usual approach of treating one problem first, using only one type of therapy.

Purpose of the study
The purpose of this study is test whether the SHM is an effective treatment for people experiencing multiple psychological difficulties. We are interested in whether SHM will reduce symptoms of multiple mental health issues in those who are suffering from both depression and one or more anxiety disorders.

What's involved?
If you decide to take part, you would be asked to attend a 'screening' session to determine whether you are eligible to participate in this study. It will take 60-90 minutes and involves interviews and questionnaires focusing on emotions and mental health.

If you are eligible to participate in the trial you will then be randomly allocated to one of two groups:

(i) 15-20 sessions of the *Shaping Healthy Minds* (SHM) programme,
(ii) Treatment you would usually receive from the NHS (Treatment-as-Usual)

You will <u>not</u> be able to choose which of these groups you are allocated to, as the allocation is random and decided by a computer.

In the SHM programme, you will complete individual sessions with a clinical psychologist. The exact therapy components that you complete will be selected in consultation with you and based on the particular symptoms you are experiencing. That is, the treatment will be shaped for you. You will also receive the regular NHS care that you normally receive except for any psychological therapy.

In Treatment-as-Usual, you will receive all of the regular NHS services you would normally receive including any psychological therapy.

In addition to receiving treatment sessions, you will be asked to attend five assessment sessions where you will be asked to complete some questionnaires, some computer-based tasks that ask about your memories and emotions, including a task that involves pleasant and unpleasant images. In addition, there is an **optional** neuroimaging task, involving two functional magnetic resonance imaging scans (before and after treatment).

For peer review only - http://bmjopen.bmj.com/site/about/guidelines.xhtml

You can choose not to complete the imaging sessions and still participate in the study.

Prior to completing the treatment sessions (SHM or Treatment-as-Usual) which form the main part of the study, you will be asked to complete one assessment session which involves interviews and questionnaires, and one assessment session with computer tasks and the optional imaging session. You would then repeat these two assessment sessions after completing the treatment, followed by one final assessment session 3 months following the completion of treatment. Each assessment session will take approximately one and a half hours, which includes a break if needed. With your consent, some sessions will be audio-recorded to allow checks on the quality of the treatment you are receiving.

Why have I been invited to take part?
All individuals currently receiving NHS services for depression or anxiety in the Cambridge area are being invited to participate in this study.

Do you have to take part?
No, it is up to you to decide. We will describe the study and go through this information sheet, which we will then give to you. If you do want to join in we'll ask you to sign a consent form, a copy of which you can keep along with this information sheet. You are free to withdraw from the study at any point *without giving us a reason*. You will not be treated any differently by any NHS service if you choose not to participate in this study or if you decide to withdraw.

Will I be reimbursed?
You will be reimbursed for the assessment sessions at a rate of £6 per hour for your time. It is anticipated that the one screening session and four assessment sessions will not exceed a total of ten hours, for which you would receive a minimum of £45, plus travel costs. If you choose to do the neuroimaging sessions as well, you will receive £20 per imaging session, so you would receive a total of £85. You will not be reimbursed for the therapy sessions as you will be receiving therapy free of charge by qualified clinical psychologists.

Are there any risks or benefits associated with taking part?
All of the tasks, interviews, and questionnaires we will ask you to complete have been used safely in previous research. As with any research involving emotional material, there is a chance that you will experience some upset when you discuss personal memories and difficulties. In our experience this is usually very mild and short-lived with no lasting ill effects. After participation you will receive a complete and thorough explanation of the study and you will be encouraged to express your feelings about your experience, if you wish. Additionally, you will be able to contact a member of the research team (a qualified clinical psychologist) after any session should you feel you are experiencing distress as a result of taking part in the study.

***What are the possible benefits of taking part*?**
We hope that the findings from this research will lead to improvement in treatment options for people experiencing multiple mental health disorders. We also hope that completing the SHM, if you are randomly allocated to that treatment, will have a beneficial impact on your psychological difficulties. We cannot guarantee that this will be the case for everybody who takes part, but you will have an opportunity to experience a range of therapeutic strategies and techniques.

What if there is a problem?
For any complaint about the way you have been dealt with during the study or any possible harm you may have suffered you can contact the Patient Advice and Liaison Service on 01223 726 774, http://www.cpft.nhs.uk/about-us/pals.htm

Further Information & Contact Details:
If you would like any further information about this project please contact the research coordinator **Dr Melissa Black** at the MRC Cognition and Brain Sciences Unit (Tel: **01223 273 739**; Email: **melissa.black@mrc-cbu.cam.ac.uk**)

Thank you for reading this information sheet

For peer review only - http://bmjopen.bmj.com/site/about/guidelines.xhtml

CAMBRIDGE CLINICAL RESEARCH CENTRE FOR AFFECTIVE DISORDERS

Cambridgeshire and Peterborough NHS Foundation Trust

MRC Cognition and Brain Sciences Unit

Participant ID Number:

Participant Consent Form (Version 2.2, April 2018)

Title of the project: Shaping Healthy Minds

Name of project coordinator: Dr Melissa Black

Please initial box:

1. I confirm that I have read and understood the Information Sheet for the above study. I have had the opportunity to consider the information, ask questions and have had these answered satisfactorily. ☐

2. I understand that my participation is voluntary and that I am free to withdraw at any time without giving any reason and without my medical care or legal rights being affected. ☐

3. I understand that data (including audio recordings) collected from me during the study may be looked at by individuals from the research team where it is relevant to my taking part in this research. I give permission for these individuals to have access to these data. ☐

4. I understand that my GP will be informed of my participation in this study if s/he is not already aware of it. ☐

5. I agree to take part in the above study. ☐

6. I agree to a researcher contacting me after the end of this study about possible future research (optional). ☐

………………………………………… ………….. …………………………..

Name of Participant Date Signature

………………………………………… ………….. ………………… ……….

Name of Person taking consent Date Signature

(When the form is completed, 1 copy is for the participant and 1 is for the study files)

For peer review only - http://bmjopen.bmj.com/site/about/guidelines.xhtml

NIH U.S. National Library of Medicine

ClinicalTrials.gov

Find Studies
About Studies
Submit Studies
Resources
About Site

Trial record **1 of 23** for: mood anxiety disorders | United Kingdom

Previous Study | Return to List | Next Study

The Modular Protocol for Mental Health (MPMH) (MPMH)

The safety and scientific validity of this study is the responsibility of the study sponsor and investigators. Listing a study does not mean it has been evaluated by the U.S. Federal Government. Read our disclaimer for details.

ClinicalTrials.gov Identifier: NCT03143634

Recruitment Status: Enrolling by invitation
First Posted: May 8, 2017
Last Update Posted: August 11, 2017

Sponsor:
Medical Research Council Cognition and Brain Sciences Unit

Collaborator:
Cambridgeshire and Peterborough NHS Foundation Trust

Information provided by (Responsible Party):
Medical Research Council Cognition and Brain Sciences Unit

Study Details | **Tabular View** | No Results Posted | Disclaimer | How to Read a Study Record

Tracking Information

**First Submitted Date** ICMJE	April 27, 2017

**First Posted Date** ICMJE	May 8, 2017
**Last Update Posted Date**	August 11, 2017
**Actual Study Start Date** ICMJE	July 18, 2017
**Estimated Primary Completion Date**	March 31, 2019 (Final data collection date for primary outcome measure)
**Current Primary Outcome Measures** ICMJE **(submitted: May 4, 2017)**	• Change in Depression symptoms [Time Frame: Baseline (within two weeks of first treatment session), within one week of the final treatment session, three-months following final treatment session]<br>Patient Health Questionnaire - 9-item version (PHQ-9; Kroenke & Spitzer, 2002)<br>• Change in Anxiety symptoms [Time Frame: Baseline (within two weeks of first treatment session), within one week of the final treatment session, three-months following final treatment session]<br>General Anxiety Disorder Questionnaire - 7-item version (GAD-7; Spitzer, Kroenke, Williams, & Lowe, 2006)<br>• Change in Level of disability and functional impairment [Time Frame: Baseline (within two weeks of first treatment session), within one week of the final treatment session, three-months following final treatment session]<br>Work and Social Adjustment Scale (WSAS; Mundt, J.C. et al., 2002)
**Original Primary Outcome Measures** ICMJE	*Same as current*
**Change History**	Complete list of historical versions of study NCT03143634 on ClinicalTrials.gov Archive Site
**Current Secondary Outcome Measures** ICMJE **(submitted: May 4, 2017)**	• Change in symptoms of social phobia, agoraphobia and specific phobia [Time Frame: Baseline (within two weeks of first treatment session), within one week of the final treatment session, three-months following final treatment session]<br>IAPT Phobia Scales (Department of Health/IAPT, 2011). This measure will only be completed by participants with symptoms of social phobia, agoraphobia or specific phobia.<br>• Change in symptoms of social anxiety [Time Frame: Baseline (within two weeks of first treatment session), within one week of the final treatment session, three-months following final treatment session]<br>The Social Phobia Inventory (SPIN; Connor, Davidson, Churchill, Sherwood, Foa, & Weisler, 2000). This measure will only be completed by participants with symptoms of social anxiety.<br>• Change in symptoms of generalised anxiety [Time Frame: Baseline (within two weeks of first treatment session), within one week of the final treatment session, three-months following final treatment session]

The Penn State Worry Questionnaire (PSWQ; Meyer, Miller, Metzger, & Borkovec, 1990). This measure will only be completed by participants with symptoms of generalised anxiety disorder.

- Change in symptoms of obsessive-compulsive disorder [Time Frame: Baseline (within two weeks of first treatment session), within one week of the final treatment session, three-months following final treatment session]

  The Obsessive-Compulsive Inventory (OCI; Foa, Kozak, Salkovskis, Coles, & Amir, 1998). This measure will only be completed by participants with symptoms of obsessive-compulsive disorder.

- Change in symptoms of post-traumatic stress disorder [Time Frame: Baseline (within two weeks of first treatment session), within one week of the final treatment session, three-months following final treatment session]

  The Revised Impact of Event Scale (IES-R; Weiss, 2007). This measure will only be completed by participants with symptoms of PTSD.

- Change in symptoms of agoraphobia [Time Frame: Baseline (within two weeks of first treatment session), within one week of the final treatment session, three-months following final treatment session]

  The Agoraphobia-Mobility Inventory (MI; Chambless, Caputo, Jasin, Gracely, & Williams, 1985). This measure will only be completed by participants with symptoms of agoraphobia.

- Change in symptoms of specific phobias [Time Frame: Baseline (within two weeks of first treatment session), within one week of the final treatment session, three-months following final treatment session]

  The Fear Questionnaire (FQ; Marks & Mathews, 1979). This measure will only be completed by participants with symptoms of specific phobia.

- Change in symptoms of panic disorder [Time Frame: Baseline (within two weeks of first treatment session), within one week of the final treatment session, three-months following final treatment session]

  The Panic Disorder Severity Scale - self report version (PDSS-SR; Shear, Brown, Barlow, Money, Sholomskas, Woods, Gorman, & Papp, 1997). This measure will only be completed by participants with symptoms of panic disorder.

- Change in symptoms of illness anxiety [Time Frame: Baseline (within two weeks of first treatment session), within one week of the final treatment session, three-months following final treatment session]

  The Health Anxiety Inventory - short version (SHAI; Salkovskis, Rimes, Warwick, & Clark, 2002). This measure will only be completed by participants with symptoms of illness anxiety.

- Change in level of disability and functional impairment [Time Frame: Baseline (within two weeks of first treatment session), within one week of the final treatment session, three-months following final treatment session]

  Sheehan Disability Scale (SDS; Sheehan, 1983)

- Change in expectancy about treatment outcome, as well as the credibility of the treatment [Time Frame: Baseline (within two weeks of first treatment session), within one week of the final treatment session, three-months following final treatment session]

	The Treatment Credibility / Expectancy Questionnaire (CEQ; Devilly & Borkovec, 2000)<br>• Change in ability to contextualize negative events within a wider frame of reference [Time Frame: Baseline (within two weeks of first treatment session), within one week of the final treatment session, three-months following final treatment session]<br>Cognitive Emotion Regulation Questionnaire (CERQ; Garnefski, Kraaij & Spinhoven, 2000)<br>• Change in disengagement from and acceptance of troublesome mental content, rumination [Time Frame: Baseline (within two weeks of first treatment session), within one week of the final treatment session, three-months following final treatment session]<br>Experiences Questionnaire (EQ; Fresco et al., 2007)<br>• Intensity with which participants experience 36 different emotions on a typical day [Time Frame: Baseline (within two weeks of first treatment session), within one week of the final treatment session, three-months following final treatment session]<br>Differential Emotions Scale (DES; Izard, 1993)<br>• Change in personality functioning on four subscales: Identity, Self-Direction, Empathy, and Intimacy [Time Frame: Baseline (within two weeks of first treatment session), within one week of the final treatment session, three-months following final treatment session]<br>Levels of Personality Functioning Scale (LPFS; Morey et al., 2017)<br>• Change in use of NHS services, other mental health services or activities, help at home, and time off work/lost income [Time Frame: Baseline (within two weeks of first treatment session), within one week of the final treatment session, three-months following final treatment session]<br>Healthlines Resource Use Questionnaire (Salisbury, O'Cathain, Edwards, Thomas, Gaunt, Hollinghurst, et al., 2016)<br>• Change in quality of life assessment of health and wellbeing, daily functioning, and general life satisfaction across multiple domains [Time Frame: Baseline (within two weeks of first treatment session), within one week of the final treatment session, three-months following final treatment session]<br>World Health Organisation (WHO) Quality of Life Inventory (WHOQOL; World Health Organisation, 1998)
**Original Secondary Outcome Measures** ICMJE	*Same as current*
**Current Other Outcome Measures** ICMJE **(submitted: May 4, 2017)**	• Change in measure of memory specificity [Time Frame: Baseline (within two weeks of first treatment session), within one week of the final treatment session]<br>Autobiographical Memory Test (Williams & Broadbent, 1986).<br>• Change in cognitive flexibility for emotional information [Time Frame: Baseline (within two weeks of first treatment session), within one week of the final treatment session]<br>Affective Card Sorting Task (Barcelo, 2003; Deveney & Deldin, 2006)

For peer review only - http://bmjopen.bmj.com/site/about/guidelines.xhtml

- Change in immediate recall and working memory for neutral information [Time Frame: Baseline (within two weeks of first treatment session), within one week of the final treatment session]

  Digit Span Task (Wechsler, 2008)

- Change in brief measure of premorbid intelligence [Time Frame: Baseline (within two weeks of first treatment session), within one week of the final treatment session]

  National Adult Reading Test (Nelson & Wilson, 1991)

- Change in emotional structure (awareness and differentiation) [Time Frame: Baseline (within two weeks of first treatment session), within one week of the final treatment session]

  Levels of Emotional Awareness Scale (Lane et al., 1990)

- Change in potential neurobiomarkers that may account for risk of disorders and/or predict treatment response (optional component of participation) [Time Frame: Baseline (within two weeks of first treatment session), within one week of the final treatment session]

  Functional connectivity during resting state MRI

- Change in levels of rumination [Time Frame: Within one-week pre-delivery of relevant treatment module; within one-week post-delivery of relevant treatment module (varied time-frame)]

  Ruminative Responses Scale of the Response Styles Questionnaire (RRS; Treynor, Gonzalez, Nolen-Hoeksema, 2003)

- Change in ability to tolerate distress [Time Frame: Within one-week pre-delivery of relevant treatment module; within one-week post-delivery of relevant treatment module (varied time-frame)]

  Distress Tolerance Scale (DTS; Simons & Gaher, 2005)

- Change in ability to label, perceive, and regulate emotions [Time Frame: Within one-week pre-delivery of relevant treatment module; within one-week post-delivery of relevant treatment module (varied time-frame)]

  Difficulties with Emotion Regulation Scale (DERS; Gratz & Roemer, 2004)

- Change in negative beliefs, thoughts and assumptions [Time Frame: Within one-week pre-delivery of relevant treatment module; within one-week post-delivery of relevant treatment module (varied time-frame)]

  Dysfunctional Attitudes Scale (DAS; Weissman, 1979)

- Change in mindful awareness [Time Frame: Within one-week pre-delivery of relevant treatment module; within one-week post-delivery of relevant treatment module (varied time-frame)]

  Kentucky Inventory of Mindfulness Skills (KIMS; Baer, Smith, & Allen, 2004)

- Change in fear of physical anxiety sensations [Time Frame: Within one-week pre-delivery of relevant treatment module; within one-week post-delivery of relevant treatment module (varied time-frame)]

  Anxiety Sensitivity Index (ASI; Reiss, Peterson, Gursky, & McNally 1986)

- Change in trauma-related beliefs and maladaptive appraisals of intrusive symptoms [Time Frame: Within one-week pre-delivery of relevant treatment module; within one-week post-delivery of relevant treatment module (varied time-frame)]

  Posttraumatic cognitions inventory - short version (PTCI; Foa, Ehlers, Clark, Tolin, Orsillo, 1999)

- Change in implementation of cognitive therapy skills [Time Frame: Within one-week pre-delivery of relevant treatment module; within one-week post-delivery of relevant treatment module (varied time-frame)]

  Skills of Cognitive Therapy (SoCT; Jarrett, 2010)

- Change in avoidance of internal experiences including thoughts, feelings, physical sensations. [Time Frame: Within one-week pre-delivery of relevant treatment module; within one-week post-delivery of relevant treatment module (varied time-frame)]

  The Multidimensional Experiential Avoidance Questionnaire (Gamez, Chmielewski, Kotov, Ruggero, & Watson, 2011)

**Original Other Outcome Measures** ICMJE	*Same as current*

Descriptive Information

**Brief Title** ICMJE	The Modular Protocol for Mental Health (MPMH)
**Official Title** ICMJE	The Modular Protocol for Mental Health (MPMH): A Pilot Randomised Clinical Trial of a Transdiagnostic Psychological Treatment for **Mood** and **Anxiety Disorders** in Adults
**Brief Summary**	Currently, our best psychological treatments for anxiety and mood disorders only focus on individual diagnoses. So, there are separate treatments for Panic Disorder, or Depressive Disorder, or Social Anxiety, etc. These 'diagnosis-specific' treatments work well for people whose problems fit neatly into a single diagnosis. However, they work far less well for people with complex problems involving multiple diagnoses, and 50% of patients fail to respond well to these existing treatments.<br><br>The purpose of this study is to test a new psychological treatment for anxiety and mood problems (the Modular Protocol for Mental Health [MPMH]). Instead of focusing on any single diagnosis, MPMH combines the best treatment techniques into 10 modules to target problems common across all of the different mood and anxiety diagnoses (e.g., intense emotions, negative thinking, upsetting memories, distressing habits). MPMH should therefore be a better treatment for the large numbers of individuals whose problems do not fit neatly into a single diagnosis and for whom any treatments targeting a single diagnosis would leave significant difficulties unaddressed.

**Detailed Description**	*Not Provided*
**Study Type** ICMJE	Interventional
**Study Phase**	Phase 1<br>Phase 2
**Study Design** ICMJE	Allocation: Randomized<br>Intervention Model: Parallel Assignment<br>Masking: Single (Outcomes Assessor)<br>Masking Description:<br>Only the outcome assessor will be masked, as this is a psychological intervention it is not possible to mask the participant, care provider or investigator.<br>Primary Purpose: Treatment
**Condition** ICMJE	• Major Depressive **Disorder**<br>• Generalized **Anxiety Disorder**<br>• Posttraumatic Stress **Disorder**<br>• Social **Anxiety Disorder**<br>• Panic **Disorder**<br>• Agoraphobia<br>• Obsessive-Compulsive **Disorder**<br>• Illness **Anxiety Disorder**
**Intervention** ICMJE	• Behavioral: The Modular Protocol for Mental Health<br>This intervention is based on evidence-based cognitive-behavioural approaches to psychological disorders and offers a flexible approach to treatment delivery that targets the maladaptive processes common to mood and anxiety disorders.<br>• Behavioral: Treatment-as-usual<br>This intervention will consist of psychological therapies delivered by high-intensity therapists or clinical psychologists.
**Study Arms**	• Experimental: The Modular Protocol for Mental Health<br>The Modular Protocol for Mental Health (Psychological Therapy) will last up to 18 regular weekly face-to-face sessions. Treatment follows a standard structured treatment session as per Cognitive Behavioural Therapy. The MPMH Treatment Manual was written by clinical

	psychologists. The selection and ordering of the modules for treatment will be delivered and determined by the Trial Clinical Psychologist in collaboration with the service-user. The treatment modules include:<br>M1. Getting Acquainted M2. Understanding Emotions M3. Managing and Tolerating Emotions M4. Behavioural activation M5. Tackling Avoidance M6. Tackling Unhelpful Thoughts M7. Tackling Unhelpful Habits M8. Overcoming Repetitive Thinking M9. Managing Upsetting Memories and Images M10. Relapse Prevention and Future Orientation<br>Intervention: Behavioral: The Modular Protocol for Mental Health<br>• Placebo Comparator: Treatment-as-usual<br>For Treatment-as-usual (Psychological Therapy), clinicians will be asked to provide whatever treatment they deem appropriate, including psychological services, medication and referral to other services. TAU will be delivered by high-intensity therapists or clinical psychologists.<br>Intervention: Behavioral: Treatment-as-usual
**Publications ***	• First MB, Williams JBW, Karg RS, Spitzer RL: Structured Clinical Interview for DSM-5—Research Version (SCID-5 for DSM-5, Research Version; SCID-5-RV). Arlington, VA, American Psychiatric Association, 2015<br>• Newby JM, McKinnon A, Kuyken W, Gilbody S, Dalgleish T. Systematic review and meta-analysis of transdiagnostic psychological treatments for anxiety and depressive disorders in adulthood. Clin Psychol Rev. 2015 Aug;40:91-110. doi: 10.1016/j.cpr.2015.06.002. Epub 2015 Jun 6. Review.<br>• Kroenke, K.; Spitzer, R.L.The PHQ-9: A new depression diagnostic and severity measure. Psychiatric Annals, Vol 32(9), Sep 2002, 509-515. http://dx.doi.org/10.3928/0048-5713-20020901-06<br>• Spitzer RL, Kroenke K, Williams JB, Löwe B. A brief measure for assessing generalized anxiety disorder: the GAD-7. Arch Intern Med. 2006 May 22;166(10):1092-7.<br>• Mundt JC, Marks IM, Shear MK, Greist JH. The Work and Social Adjustment Scale: a simple measure of impairment in functioning. Br J Psychiatry. 2002 May;180:461-4.<br>• IAPT/Department of Health, The IAPT data handbook version 2.0.1. 2011: Published to Department of Health Website, in electronic PDF format only<br>• Connor KM, Davidson JR, Churchill LE, Sherwood A, Foa E, Weisler RH. Psychometric properties of the Social Phobia Inventory (SPIN). New self-rating scale. Br J Psychiatry. 2000 Apr;176:379-86.<br>• Meyer TJ, Miller ML, Metzger RL, Borkovec TD. Development and validation of the Penn State Worry Questionnaire. Behav Res Ther. 1990;28(6):487-95.<br>• Foa, E.B., Kozak, M.J., Salkovskis, P.M., Coles, M.E., and Amir, N. (1998). The validation of a new obsessive-compulsive disorder scale: The Obsessive-Compulsive Inventory. Psychological Assessment, 10(3), 206-214.

- Weiss, D.S., The Impact of Event Scale: Revised, in Cross-cultural assessment of psychological trauma and PTSD, J.P. Wilson and C.S. Tang, Editors. 2007, Springer: New York. p. 219-238.
- Chambless DL, Caputo GC, Jasin SE, Gracely EJ, Williams C. The Mobility Inventory for Agoraphobia. Behav Res Ther. 1985;23(1):35-44.
- Marks IM, Mathews AM. Brief standard self-rating for phobic patients. Behav Res Ther. 1979;17(3):263-7.
- Shear MK, Brown TA, Barlow DH, Money R, Sholomskas DE, Woods SW, Gorman JM, Papp LA. Multicenter collaborative panic disorder severity scale. Am J Psychiatry. 1997 Nov;154(11):1571-5.
- Salkovskis PM, Rimes KA, Warwick HM, Clark DM. The Health Anxiety Inventory: development and validation of scales for the measurement of health anxiety and hypochondriasis. Psychol Med. 2002 Jul;32(5):843-53.
- Sheehan, D.V., The Anxiety Disease. 1983, New York: Charles Scribner and Sons.
- Devilly GJ, Borkovec TD. Psychometric properties of the credibility/expectancy questionnaire. J Behav Ther Exp Psychiatry. 2000 Jun;31(2):73-86.
- Garnefski N, Rieffe C, Jellesma F, Terwogt MM, Kraaij V. Cognitive emotion regulation strategies and emotional problems in 9 - 11-year-old children: the development of an instrument. Eur Child Adolesc Psychiatry. 2007 Feb;16(1):1-9. Epub 2006 Jun 21.
- Fresco DM, Moore MT, van Dulmen MH, Segal ZV, Ma SH, Teasdale JD, Williams JM. Initial psychometric properties of the experiences questionnaire: validation of a self-report measure of decentering. Behav Ther. 2007 Sep;38(3):234-46. Epub 2007 Apr 24.
- Izard CE, Libero DZ, Putnam P, Haynes OM. Stability of emotion experiences and their relations to traits of personality. J Pers Soc Psychol. 1993 May;64(5):847-60.
- Morey LC. Development and initial evaluation of a self-report form of the DSM-5 Level of Personality Functioning Scale. Psychol Assess. 2017 Oct;29(10):1302-1308. doi: 10.1037/pas0000450. Epub 2017 Feb 27.
- Development of the World Health Organization WHOQOL-BREF quality of life assessment. The WHOQOL Group. Psychol Med. 1998 May;28(3):551-8.
- Salisbury C, O'Cathain A, Edwards L, Thomas C, Gaunt D, Hollinghurst S, Nicholl J, Large S, Yardley L, Lewis G, Foster A, Garner K, Horspool K, Man MS, Rogers A, Pope C, Dixon P, Montgomery AA. Effectiveness of an integrated telehealth service for patients with depression: a pragmatic randomised controlled trial of a complex intervention. Lancet Psychiatry. 2016 Jun;3(6):515-25. doi: 10.1016/S2215-0366(16)00083-3. Epub 2016 Apr 27.
- Treynor, W., R. Gonzalez, and S. Nolen-Hoeksema, Rumination Reconsidered: A Psychometric Analysis. Cognitive Therapy and Research 2003. 27(3): p. 247-259.
- Simons, J.S. and R.M. Gaher, The Distress Tolerance Scale: Development and Validation of a Self-Report Measure. Motivation and Emotion, 2005. 29(2): p. 83-102.
- Gratz, K.L. & Roemer, L. Journal of Psychopathology and Behavioral Assessment (2004) 26: 41. doi:10.1023/B:JOBA.0000007455.08539.94

- Weissman, A.N., The Dysfunctional Attitude Scale: A Validation Study. 1979, University of Pennsylvania.
- Baer RA, Smith GT, Allen KB. Assessment of mindfulness by self-report: the Kentucky inventory of mindfulness skills. Assessment. 2004 Sep;11(3):191-206.
- Reiss S, Peterson RA, Gursky DM, McNally RJ. Anxiety sensitivity, anxiety frequency and the prediction of fearfulness. Behav Res Ther. 1986;24(1):1-8.
- Foa, E.B., Ehlers, A., Clark, D.M., Tolin, D.F., Orsillo, S.M., The Posttraumatic Cognitions Inventory (PTCI): Development and validation. Psychological Assessment, 1999. 11(3): p. 303-314.
- Jarrett RB, Vittengl JR, Clark LA, Thase ME. Skills of Cognitive Therapy (SoCT): a new measure of patients' comprehension and use. Psychol Assess. 2011 Sep;23(3):578-86. doi: 10.1037/a0022485.
- Gámez W, Chmielewski M, Kotov R, Ruggero C, Watson D. Development of a measure of experiential avoidance: the Multidimensional Experiential Avoidance Questionnaire. Psychol Assess. 2011 Sep;23(3):692-713. doi: 10.1037/a0023242.
- Williams JM, Broadbent K. Autobiographical memory in suicide attempters. J Abnorm Psychol. 1986 May;95(2):144-9.
- Deveney CM, Deldin PJ. A preliminary investigation of cognitive flexibility for emotional information in major depressive disorder and non-psychiatric controls. Emotion. 2006 Aug;6(3):429-37.
- Barceló F. The Madrid card sorting test (MCST): a task switching paradigm to study executive attention with event-related potentials. Brain Res Brain Res Protoc. 2003 Mar;11(1):27-37.
- Wechsler, D. (2008). Wechsler Adult Intelligence Scale—Fourth Edition. San Antonio, TX: Pearson.
- Nelson HE, Wilson J (1991) National Adult Reading Test (NART), NFER-Nelson, Windsor, UK.
- Lane RD, Quinlan DM, Schwartz GE, Walker PA, Zeitlin SB. The Levels of Emotional Awareness Scale: a cognitive-developmental measure of emotion. J Pers Assess. 1990 Fall;55(1-2):124-34.

*** Includes publications given by the data provider as well as publications identified by ClinicalTrials.gov Identifier (NCT Number) in Medline.**

Recruitment Information

**Recruitment Status** ICMJE	Enrolling by invitation
**Estimated Enrollment** ICMJE **(submitted: May 4, 2017)**	50

**Original Estimated Enrollment** ICMJE	*Same as current*
**Estimated Study Completion Date**	March 31, 2019
**Estimated Primary Completion Date**	March 31, 2019 (Final data collection date for primary outcome measure)
**Eligibility Criteria** ICMJE	Inclusion Criteria:<br>• Patients with a primary diagnosis of a unipolar mood, anxiety, stress or obsessive-compulsive disorder with at least one additional comorbid diagnosis according to the DSM-5. The criteria for diagnosis will be based on the Structured Clinical Interview for the DSM (SCID) which assesses disorders according to DSM-5 diagnostic criteria.<br>• To be eligible participants will also need to score >10 on either the Patient Health Questionnaire (PHQ-9) or Generalised Anxiety Disorder -7 item scale, GAD-7 (see Study Measures above).<br>Exclusion Criteria:<br>• Current/past psychosis or bipolar disorder<br>• Current full diagnosis of substance use disorder<br>• Organic brain damage<br>• Complex trauma history or recurrent self-injury requiring specialist services (as deemed by the NHS Clinical Care team)<br>• Current suicidality that warrants immediate clinical attention and constitutes a current risk of harm to the individual.
**Sex/Gender**	Sexes Eligible for Study: All
**Ages**	18 Years to 65 Years (Adult)
**Accepts Healthy Volunteers**	No
**Contacts** ICMJE	*Contact information is only displayed when the study is recruiting subjects*
**Listed Location Countries** ICMJE	**United Kingdom**
**Removed Location Countries**	

Administrative Information

NCT Number ICMJE	NCT03143634
Other Study ID Numbers ICMJE	MP:MH RfPB PB-PG-0214-33072
Has Data Monitoring Committee	No
U.S. FDA-regulated Product	Studies a U.S. FDA-regulated Drug Product: No<br>Studies a U.S. FDA-regulated Device Product: No
IPD Sharing Statement	Plan to Share IPD: Undecided
Responsible Party	Medical Research Council Cognition and Brain Sciences Unit
Study Sponsor ICMJE	Medical Research Council Cognition and Brain Sciences Unit
Collaborators ICMJE	Cambridgeshire and Peterborough NHS Foundation Trust
Investigators ICMJE	Principal Investigator: Tim Dalgleish, PhD Medical Research Council Cognition and Brain Sciences Unit
PRS Account	Medical Research Council Cognition and Brain Sciences Unit
Verification Date	August 2017

ICMJE **Data element required by the International Committee of Medical Journal Editors and the World Health Organization ICTRP**

SPIRIT

STANDARD PROTOCOL ITEMS: RECOMMENDATIONS FOR INTERVENTIONAL TRIALS

SPIRIT 2013 Checklist: Recommended items to address in a clinical trial protocol and related documents*

Section/item	Item No	Description	Page/Line Reference
**Administrative information**			
Title	1	Descriptive title identifying the study design, population, interventions, and, if applicable, trial acronym	p. 1, line 1
Trial registration	2a	Trial identifier and registry name. If not yet registered, name of intended registry	p. 1, line 28
	2b	All items from the World Health Organization Trial Registration Data Set	See attached
Protocol version	3	Date and version identifier	p. 1, line 29
Funding	4	Sources and types of financial, material, and other support	p. 1, line 30<br>p. 22, line 1
Roles and responsibilities	5a	Names, affiliations, and roles of protocol contributors	p. 1, line 4
	5b	Name and contact information for the trial sponsor	p. 22, line 20
	5c	Role of study sponsor and funders, if any, in study design; collection, management, analysis, and interpretation of data; writing of the report; and the decision to submit the report for publication, including whether they will have ultimate authority over any of these activities	p. 22, line 1
	5d	Composition, roles, and responsibilities of the coordinating centre, steering committee, endpoint adjudication committee, data management team, and other individuals or groups overseeing the trial, if applicable (see Item 21a for data monitoring committee)	p. 16, line 16
**Introduction**			

For peer review only

Background and rationale	6a	Description of research question and justification for undertaking the trial, including summary of relevant studies (published and unpublished) examining benefits and harms for each intervention	p. 4, line 4
	6b	Explanation for choice of comparators	p. 8, line 6
Objectives	7	Specific objectives or hypotheses	p. 8, line 9
Trial design	8	Description of trial design including type of trial (eg, parallel group, crossover, factorial, single group), allocation ratio, and framework (eg, superiority, equivalence, noninferiority, exploratory)	p. 8, line 23

Methods: Participants, interventions, and outcomes

Study setting	9	Description of study settings (eg, community clinic, academic hospital) and list of countries where data will be collected. Reference to where list of study sites can be obtained	p. 9, line 23
Eligibility criteria	10	Inclusion and exclusion criteria for participants. If applicable, eligibility criteria for study centres and individuals who will perform the interventions (eg, surgeons, psychotherapists)	p. 9, line 4<br>p. 9, line 12<br>p. 12, line 15
Intervention s	11a	Interventions for each group with sufficient detail to allow replication, including how and when they will be administered	p. 10, line 21
	11b	Criteria for discontinuing or modifying allocated interventions for a given trial participant (eg, drug dose change in response to harms, participant request, or improving/worsening disease)	p. 10, line 17
	11c	Strategies to improve adherence to intervention protocols, and any procedures for monitoring adherence (eg, drug tablet return, laboratory tests)	p. 12, line 15<br>p. 17, line 5
	11d	Relevant concomitant care and interventions that are permitted or prohibited during the trial	p. 9, line 20
Outcomes	12	Primary, secondary, and other outcomes, including the specific measurement variable (eg, systolic blood pressure), analysis metric (eg, change from baseline, final value, time to event), method of aggregation (eg, median, proportion), and time point for each outcome. Explanation of the clinical relevance of chosen efficacy and harm outcomes is strongly recommended	p. 13, line 9

Participant timeline	13	Time schedule of enrolment, interventions (including any run-ins and washouts), assessments, and visits for participants. A schematic diagram is highly recommended (see Figure)	p. 9, line 23 Figure 1
Sample size	14	Estimated number of participants needed to achieve study objectives and how it was determined, including clinical and statistical assumptions supporting any sample size calculations	p. 15, line 2
Recruitment	15	Strategies for achieving adequate participant enrolment to reach target sample size	p. 9, line 23

Methods: Assignment of interventions (for controlled trials)

Allocation:

Sequence generation	16a	Method of generating the allocation sequence (eg, computer-generated random numbers), and list of any factors for stratification. To reduce predictability of a random sequence, details of any planned restriction (eg, blocking) should be provided in a separate document that is unavailable to those who enrol participants or assign interventions	p. 10, line 10
Allocation concealment mechanism	16b	Mechanism of implementing the allocation sequence (eg, central telephone; sequentially numbered, opaque, sealed envelopes), describing any steps to conceal the sequence until interventions are assigned	p. 10, line 10
Implementation	16c	Who will generate the allocation sequence, who will enrol participants, and who will assign participants to interventions	p. 10, line 14
Blinding (masking)	17a	Who will be blinded after assignment to interventions (eg, trial participants, care providers, outcome assessors, data analysts), and how	p. 15, line 20
	17b	If blinded, circumstances under which unblinding is permissible, and procedure for revealing a participant's allocated intervention during the trial	p. 15, line 20

Methods: Data collection, management, and analysis

Data collection methods	18a	Plans for assessment and collection of outcome, baseline, and other trial data, including any related processes to promote data quality (eg, duplicate measurements, training of assessors) and a description of study instruments (eg, questionnaires, laboratory tests) along with their reliability and validity, if known. Reference to where data collection forms can be found, if not in the protocol	p. 13, line 8<br>p. 15, line 13
	18b	Plans to promote participant retention and complete follow-up, including list of any outcome data to be collected for participants who discontinue or deviate from intervention protocols	p. 16, line 16
Data manageme nt	19	Plans for data entry, coding, security, and storage, including any related processes to promote data quality (eg, double data entry; range checks for data values). Reference to where details of data management procedures can be found, if not in the protocol	p. 15, line 17<br>p. 16, line 16
Statistical methods	20a	Statistical methods for analysing primary and secondary outcomes. Reference to where other details of the statistical analysis plan can be found, if not in the protocol	p. 16, line 1
	20b	Methods for any additional analyses (eg, subgroup and adjusted analyses)	p. 16, line 10
	20c	Definition of analysis population relating to protocol non-adherence (eg, as randomised analysis), and any statistical methods to handle missing data (eg, multiple imputation)	p. 16, line 9
**Methods: Monitoring**			
Data monitoring	21a	Composition of data monitoring committee (DMC); summary of its role and reporting structure; statement of whether it is independent from the sponsor and competing interests; and reference to where further details about its charter can be found, if not in the protocol. Alternatively, an explanation of why a DMC is not needed	p. 16, line 16
	21b	Description of any interim analyses and stopping guidelines, including who will have access to these interim results and make the final decision to terminate the trial	p. 16, line 3

Harms	22	Plans for collecting, assessing, reporting, and managing solicited and spontaneously reported adverse events and other unintended effects of trial interventions or trial conduct	p. 17, line 5
Auditing	23	Frequency and procedures for auditing trial conduct, if any, and whether the process will be independent from investigators and the sponsor	p. 16, line 17
**Ethics and dissemination**			
Research ethics approval	24	Plans for seeking research ethics committee/institutional review board (REC/IRB) approval	p. 17, line 17
Protocol amendments	25	Plans for communicating important protocol modifications (eg, changes to eligibility criteria, outcomes, analyses) to relevant parties (eg, investigators, REC/IRBs, trial participants, trial registries, journals, regulators)	p. 16, line 16<br>p. 17, line 21
Consent or assent	26a	Who will obtain informed consent or assent from potential trial participants or authorised surrogates, and how (see Item 32)	p. 10, line 7
	26b	Additional consent provisions for collection and use of participant data and biological specimens in ancillary studies, if applicable	N/A
Confidentiality	27	How personal information about potential and enrolled participants will be collected, shared, and maintained in order to protect confidentiality before, during, and after the trial	p. 15, line 13
Declaration of interests	28	Financial and other competing interests for principal investigators for the overall trial and each study site	p. 22, line 11
Access to data	29	Statement of who will have access to the final trial dataset, and disclosure of contractual agreements that limit such access for investigators	p. 17, line 2
Ancillary and post-trial care	30	Provisions, if any, for ancillary and post-trial care, and for compensation to those who suffer harm from trial participation	p. 17, line 14
Dissemination policy	31a	Plans for investigators and sponsor to communicate trial results to participants, healthcare professionals, the public, and other relevant groups (eg, via publication, reporting in results databases, or other data sharing arrangements), including any publication restrictions	p. 17, line 24

For peer review only

	31b	Authorship eligibility guidelines and any intended use of professional writers	p. 21, line 1
	31c	Plans, if any, for granting public access to the full protocol, participant-level dataset, and statistical code	p. 17, line 24
**Appendices**			
Informed consent materials	32	Model consent form and other related documentation given to participants and authorised surrogates	Supplementary materials
Biological specimens	33	Plans for collection, laboratory evaluation, and storage of biological specimens for genetic or molecular analysis in the current trial and for future use in ancillary studies, if applicable	N/A

*It is strongly recommended that this checklist be read in conjunction with the SPIRIT 2013 Explanation & Elaboration for important clarification on the items. Amendments to the protocol should be tracked and dated. The SPIRIT checklist is copyrighted by the SPIRIT Group under the Creative Commons "Attribution-NonCommercial-NoDerivs 3.0 Unported" license.

For peer review only

BMJ Open

The HARMONIC trial: Study protocol for a randomised controlled feasibility trial of Shaping Healthy Minds – a modular transdiagnostic intervention for mood, stressor-related and anxiety disorders in adults

Journal:	*BMJ Open*
Manuscript ID	bmjopen-2018-024546.R1
Article Type:	Protocol
Date Submitted by the Author:	15-Jun-2018
Complete List of Authors:	Black, Melissa; MRC Cognition and Brain Sciences Unit, University of Cambridge; Cambridgeshire and Peterborough NHS Foundation Trust<br>Hitchcock, Caitlin; University of Cambridge, MRC Cognition and Brain Sciences Unit; Cambridgeshire and Peterborough NHS Foundation Trust<br>Bevan, Anna; MRC Cognition and Brain Sciences Unit, University of Cambridge<br>O Leary, Cliodhna; University of Cambridge, MRC Cognition and Brain Sciences Unit; Cambridgeshire and Peterborough NHS Foundation Trust<br>Clarke, James; Cambridgeshire and Peterborough NHS Foundation Trust<br>Elliott, Rachel; MRC Cognition and Brain Sciences Unit, University of Cambridge; Cambridgeshire and Peterborough NHS Foundation Trust<br>Watson, Peter; MRC Cognition and Brain Sciences Unit<br>LaFortune, Louise; University of Cambridge, Institute of Public Health<br>Rae, Sarah; Cambridgeshire and Peterborough NHS Foundation Trust<br>Gilbody, Simon; The University of York, Department of Health Sciences<br>Kuyken, Willem; University of Oxford, Department of Psychiatry<br>Johnston, David; MRC Cognition and Brain Sciences Unit, University of Cambridge; Cambridgeshire and Peterborough NHS Foundation Trust<br>Newby, Jill M.; UNSW, Clinical Research Unit for Anxiety and Depression<br>Dalgleish, Tim; Medical Research Council Cognition and Brain Sciences Unit; Cambridgeshire and Peterborough NHS Foundation Trust
**Primary Subject Heading**:	Mental health
Secondary Subject Heading:	Research methods
Keywords:	Transdiagnostic, Depression, Anxiety, Posttraumatic Stress Disorder, Common Mental Health Problems

SCHOLARONE™
Manuscripts

The HARMONIC trial: Study protocol for a randomised controlled feasibility trial of *Shaping Healthy Minds* – a modular transdiagnostic intervention for mood, stressor-related and anxiety disorders in adults

Melissa Black[1,2]
Caitlin Hitchcock[1,2]
Anna Bevan[1]
Cliodhna O Leary[1,2]
James Clarke[2]
Rachel Elliott[1,2]
Peter Watson[1]
Louise Lafortune[5]
Sarah Rae[2]
Simon Gilbody[6]
Willem Kuyken[4]
David Johnston[1,2]
Jill Newby[3]
Tim Dalgleish[1,2]

1. Medical Research Council Cognition and Brain Sciences Unit, University of Cambridge, Cambridge UK
2. Cambridgeshire and Peterborough NHS Foundation Trust
3. School of Psychology, UNSW Sydney
4. Department of Psychiatry, University of Oxford
5. Cambridge Institute of Public Health, University of Cambridge
6. Department of Health Sciences, University of York

Corresponding Author: Tim Dalgleish (tim.dalgleish@mrc-cbu.cam.ac.uk)

Trial Registration: NCT03143634 (ClinicalTrials.gov)
Protocol Version: First, 31/05/2018
Funding: National Institute of Health Research, Research for Patient Benefit (RfPB: PB-PG-0214-33072)

Word count: 4652 words

For peer review only

Abstract

Introduction: Anxiety, mood and trauma-related disorders are common, affecting up to 20% of adults. Many of these individuals will experience symptoms of more than one disorder as diagnostically defined. However, most psychological treatments focus on individual disorders and are less effective for those who experience comorbid disorders. The HARMONIC trial introduces a novel transdiagnostic intervention (*Shaping Healthy Minds*), which synthesises several evidence-based treatment techniques to address the gap in effective interventions for people with complex and comorbid difficulties. This early-phase trial aims to estimate the efficacy and feasibility of the transdiagnostic intervention in preparation for a later-phase randomised controlled trial, and to explore mechanisms of change.

Methods/Analysis: We outline a patient-level two-arm randomised controlled trial (HARMONIC) that compares *Shaping Healthy Minds* to treatment-as-usual (TAU) for individuals aged >18 years (N=50) with co-morbid mood, anxiety, obsessive-compulsive or trauma/stressor disorder diagnoses, recruited from outpatient psychological services within the UK National Health Service. The co-primary outcomes will be 3-month follow-up scores on self-report measures of depressive symptoms, anxiety symptoms, and disability and functional impairment. Secondary outcomes include changes in symptoms linked to individual disorders. We will assess the feasibility and acceptability of *Shaping Healthy Minds*, the utility of proposed outcome measures, and refine the treatment manuals in preparation for a later-phase trial.

Ethics and dissemination: This trial protocol has been approved by the Health Research Authority of the National Health Service of the United Kingdom (East of England, Reference: 16/EE/0095). We anticipate that trial findings will inform future revisions of clinical guidelines for numerous forms of mood, anxiety, and stressor-related disorders. Findings will be disseminated broadly via peer-reviewed empirical journal articles, conference presentations, clinical workshops, and a trial website.

For peer review only

Key Words: Transdiagnostic, Depression, Anxiety, Posttraumatic Stress Disorder, Common Mental Health Problems

Trial registration: Clinicaltrials.gov identifier: NCT03143634.

Strengths and limitations of this study

- The first study to investigate the feasibility and procedural uncertainties of a flexibly-delivered modular transdiagnostic treatment protocol – *Shaping Healthy Minds* – in adults with unipolar mood, anxiety, and trauma-related disorders.
- This trial will provide a point estimate of efficacy of the *Shaping Healthy Minds* protocol, relative to TAU, in preparation for a later-stage trial, and explore putative mediators and moderators of treatment outcome.
- Comparison of *Shaping Healthy Minds* against treatment-as-usual (TAU) currently provided by the NHS will provide a rigorous evaluation of treatment potential
- Administering self-report questionnaires that are specific to each service-user's secondary diagnoses may limit the ability to draw group-based conclusions

For peer review only

The HARMONIC trial: Study protocol for a randomised controlled feasibility trial of *Shaping Healthy Minds* – a modular transdiagnostic intervention for mood, stressor-related and anxiety disorders in adults

Mood, stressor-related, obsessive-compulsive and anxiety disorders – so called common mental health problems (CMHP; NICE, 2011) – are one of the largest causes of disability in the world, with 16 – 20% of adults affected at any given time [1, 2]. Maximising our ability to treat CMHP in cost effective, efficient, and effective ways that can be widely disseminated is a priority [2]. At present, there is a range of complex psychological treatments with demonstrated efficacy in the treatment of CMHP, and in preventing recurrence. Consequently, the National Institute of Health and Care Excellence (NICE) recommends psychological treatment at various points in the care pathway to all those suffering from such problems, although there are not specific recommendations for individuals experiencing more than one problem [3]. Between 40 – 80% of patients experiencing a CMHP also experience an additional comorbid CMHP [4, 5]. Even our best available psychological treatments only achieve clinical recovery for 40 – 70% of patients, depending on their primary CMHP, with people suffering complex co-morbid conditions faring significantly worse [6]. For the majority of patients who receive treatment, there remains a significant risk of future relapse [7, 8]. A key challenge therefore is how we can build on and extend beyond the current psychological treatments for CMHP to increase efficacy, and sustained recovery, particularly for those with co-morbid, recurrent, and complex presentations [9].

Over the past decade, there has been a major shift in the conceptualisation of CMHP, away from a single-diagnosis approach in favour of a transdiagnostic model [10, 11]. There is strong empirical and theoretical support for development of transdiagnostic treatment approaches, as many of the cognitive, emotional, behavioural, and interpersonal factors which drive symptomology are consistent across disorders [12, 13]. A transdiagnostic approach

For peer review only

thereby has the potential to improve the efficacy and efficiency of treatment for people with anxiety, stress and depression.

There are potential limitations to the commonly-utilised single-disorder-focused treatment approach. First, with the exception of a few existing programmes [14, 15], most evidence-based treatment protocols are single-disorder-focused programs (e.g., depression [16], generalised anxiety [17, 18], social anxiety [19], and post-traumatic stress disorder [20]). Comorbid conditions and disorders are either ignored, or minimally treated within these treatment packages. This leaves a mismatch between the available evidence base and the clinical reality which clinicians face: the majority of people with any given CMHP have at least one or more comorbid disorders to their primary diagnosis [4]. Second, in the attempt to manualise treatments, most packages are inflexible 'one-size-fits-all' approaches, leaving patients with a wide range of problems and presentations receiving the same treatment package, regardless of their symptoms, goals, and concerns [14]. Third, in practice, many clinicians already deliver evidence-based psychological treatments in a flexible manner in order to address individual concerns and goals. Manualised treatments need to better reflect the realities of service-user experiences and treatment delivery. This approach merits improvement so that delivered treatments are more efficient, effective, and personalised to individuals' concerns.

Existing psychological treatments for CMHP share more similarities than differences [21]. Despite differences in the theoretical foundation underlying available psychological treatments, and the terms used to describe maintaining factors and treatment targets, there are many common elements. For instance, psychoeducation, graded exposure, mindfulness techniques, and behavioural activation form a key component of a variety of effective treatments such as trauma-focused cognitive behavioural therapy, cognitive behavioural therapy (CBT), acceptance and commitment therapy (ACT), Dialectical Behaviour Therapy

For peer review only

(DBT), exposure therapy and mindfulness-based cognitive therapy (MBCT). The widespread availability of so many treatment options has the potential to elicit considerable decision-making difficulties for the treating clinician. In both formulation and treatment planning, challenging decisions occur when selecting the order in which to treat multiple difficulties, in evaluating the most appropriate treatment approach, and working out which treatment option will be acceptable and effective for the client.

A recent equivalence RCT demonstrated that a transdiagnostic protocol (The *Unified Protocol for Transdiagnostic Treatment of Emotional Disorders* [22]) and single-disorder protocols produced statistically equivalent reduction in severity of principal anxiety disorder diagnosis, but that there was less attrition in the transdiagnostic group [23]. Promising results have also been found for other transdiagnostic treatment protocols, including Norton's Transdiagnostic Group Cognitive Behavioral Therapy for Anxiety [15], Gros's Transdiagnostic Behavior Therapy for affective disorders [24], and Schmidt's False Safety Behaviour Elimination Therapy for Anxiety Disorders [25]. In addition, our systematic review and meta-analysis supported the overall efficacy of transdiagnostic treatments [10]. The review called for more high-quality studies to resolve uncertainties surrounding the heterogeneity of treatment effects and to determine the best treatment approaches and designs. We aim to address these issues through evaluating a novel intervention which combines a number of evidence-based treatment strategies. In utilising a modular approach, this trial will contribute to identification and evaluation of effective treatment components and delivery method. The modular approach to treatment design incorporates self-contained functional units (therapy modules) that can operate independently and be delivered flexibly, but also refer to other modules if needed [22]. A complex, modular, tailored transdiagnostic intervention that targets common underlying processes maximises goodness of fit and has a

For peer review only

direct focus on process rather than symptoms. The approach is thereby suitable for complexity and comorbidity as well as sub-syndromal and prodromal symptoms.

The transdiagnostic intervention we have developed – *Shaping Healthy Minds* – targets the processes and symptoms that are common to CMHPs and offers a number of advances in transdiagnostic treatment by incorporating the best available techniques from existing manual-based treatments into the one treatment package. A key aim of the programme is to encompass the treatment techniques that skilled psychologists and mental health clinicians already implement in standard practice for depressed, stressed and anxious patients with complex presentations [26]. The intervention also builds on the Unified Protocol for Transdiagnostic Treatment for Emotional Disorders described by Barlow et al. [14], by working towards a prescriptive approach for the delivery of treatment modules based on the formulation of the client's presenting difficulties [9]. Specifically, the treatment expands beyond interventions grounded in a sole treatment paradigm (e.g., CBT) towards a theory-driven approach that utilises efficacious techniques translated from basic science alongside components drawn from a wide range of evidence-based psychological treatments (e.g., Mindfulness-based interventions, ACT, Behavioural Activation, and DBT).

The treatment protocol for *Shaping Healthy Minds* also changes the way that standardised manual-based treatments are delivered. Rather than using integral interventions (where all patients receive the same relatively fixed, complete protocol), the transdiagnostic intervention is a modular intervention, whereby the assessment of core problematic areas of emotional, cognitive, interpersonal, and behavioural processes informs the selection and sequence of treatment modules targeted at specific problem areas for patients [22]. This modular approach allows for standardised, yet flexible treatment that is personalised to the individual concerns, problems, and goals for the patient. Finally, it expands beyond interventions that typically focus on alleviating negative symptomatology (e.g., negative

For peer review only

thoughts, excessive negative emotions) and incorporates interventions designed to increase positive emotions, capture strengths, and enhance resilience for sustained recovery.

We aim to examine the feasibility of *Shaping Healthy Minds* in reducing symptoms of depression, anxiety, distress, disability and functional impairment through an early-stage randomised clinical trial, in line with recommendations for the development of complex interventions [27]. In particular, we will gather data on the extent to which *Shaping Healthy Minds* performs comparably to TAU for a given service-user's primary diagnosed problem [10, 28] as well as for other significant additional, secondary, and/or comorbid difficulties. In addition, this trial will provide a preliminary evaluation of whether a modular transdiagnostic treatment approach may be effective at reducing the distress and impairment associated with CMHP [10]. The trial will also provide an indication of the feasibility and acceptability, of the transdiagnostic intervention to service-users and clinicians by recruiting through post-primary care UK NHS psychology services where complex comorbidity represents the modal clinical presentation. In addition, the trial will provide initial estimates of cost-effectiveness in terms of service-use and potential quality-adjusted life years added. We therefore present the protocol for a feasibility trial with co-primary outcomes, examining the effect of *Shaping Healthy Minds* on primary and comorbid diagnoses. The feasibility trial will provide a plausible range of point estimates of the efficacy of *Shaping Healthy Minds* on standardised continuous symptom measures for primary and secondary diagnoses to inform this key question, refine the treatment manual and contribute to the design of future scaled-up trials [27].

Methods and Analysis

This trial protocol is written in compliance with the Standard Protocol Items: Recommendations for Interventional Trials (SPIRIT) guidelines.

For peer review only

Study Design

The design is a parallel arm RCT comparing *Shaping Healthy Minds* to TAU. Participants will be assessed three times - at baseline, at post-treatment, and at 3-month follow-up. These three time points involve face-to-face assessments including the full battery of primary and additional outcomes and process measures, described below.

Participants and recruitment

The proposed feasibility study will seek to recruit 50 people aged 18 and above with a primary diagnosis of a unipolar mood, anxiety, obsessive-compulsive, or trauma- and stressor-related disorder (CMHPs) with at least one additional comorbid diagnosis according to the Diagnostic and Statistical Manual of Mental Disorders – Fifth Edition (DSM-5 [29]). Participants will be randomly allocated to one of two groups: (1) *Shaping Healthy Minds*, or (2) TAU. Diagnosis of CMHPs will be determined by trained research staff using the Structured Clinical Interview for the DSM-5 (SCID-5; [30]). To be eligible participants will also need to score > 10 on either the Patient Health Questionnaire (PHQ-9) or the Generalised Anxiety Disorder Questionnaire (GAD-7; see Study Measures below). Exclusion criteria are current/past psychosis or bipolar disorder, current diagnosis of alcohol or substance use disorder (all assessed via the Structured Clinical Interview for the DSM-5; SCID-5; [30]), organic brain damage, complex trauma history or recurrent self-injury requiring specialist services, or current suicidality that warrants immediate clinical attention and constitutes a current risk of harm to the individual (all assessed via participant report and the clinical care team). Participants may be engaged with the multi-disciplinary clinical care team, but those randomised to *Shaping Healthy Minds* will not be receiving other psychological services whilst participating in the trial. All other services (e.g., medication review with psychiatrist or general practitioner, occupational therapy, social support services) may be continued, and there are no medication exclusions.

For peer review only

Participants will be recruited through local NHS clinical psychology services, including the high intensity team of the Cambridge Psychological Wellbeing Service, and secondary care services with expertise in treatment of more complex and comorbid affective disorders. The recruitment pathways will involve suitable service-users on a waitlist to receive treatment being identified by a member of the clinical service (including an Assistant Psychologist focusing primarily on recruitment into clinical research studies) who will provide them with a letter outlining the study. Service-users will then be able to contact the research team to opt into the study. Initial eligibility will be screened over the telephone, and suitable participants will be invited to complete the SCID, either at the clinical service or the research unit. At the beginning of this session, all participants will provide written informed consent (see supplementary materials for a sample Participant Information Sheet and Consent Form, Supplementary File 1). No potential participants will be contacted by a member of the research team until they have given consent for such contact to a member of the clinical care team.

Participant allocation

Following both baseline assessment sessions, eligible participants will be stratified according to depression (PHQ-9) and anxiety (GAD-7) severity scores and randomised to either *Shaping Healthy Minds* (n = 25) or TAU (n = 25). This will be achieved using computer generated, quasi-random numbers and will be conducted by the trial statistician (PW), blind to study objectives. Once generated, this information is passed to the project coordinator responsible for delivering the intervention. Once a participant begins treatment, he or she is free to discontinue participation at any time, in which case s/he will be referred back to the appropriate NHS clinical care team. Figure 1 summarises participant flow through the trial.

For peer review only

Interventions

Shaping Healthy Minds (SHM) is a modular intervention, comprising 10 independent modules that will last up to 20 sessions. The content of the modules is drawn from a number of evidence-based psychological therapies, including CBT [31], ACT [32], DBT [33], MBCT [34], and behavioural activation [35, 36]. The programme aims to bring together the core and unique therapeutic techniques from the best available disorder-focused treatment packages into the one transdiagnostic treatment package (e.g., behavioural experiments [37] and graded exposure [38] from CBT, values exercises and mindfulness strategies from ACT [39], activity scheduling from BA [35], emotion regulation strategies from DBT [33], and present-moment awareness exercises from MBCT [40]). The elements of *Shaping Healthy Minds* were drawn from recent meta-analyses supporting the effectiveness of these treatment strategies (e.g., [36, 41-43]), and the manuals were written and reviewed by experienced clinical psychologists (TD, JN, AB, CH, and MB). In addition, experts in particular fields (e.g., WK for mindfulness and case formulation) were consulted on the content of specific modules.

The modular approach is standardised, yet can be flexibly delivered according to an individual's concerns, problems, and goals [22]. The treatment focuses both on alleviating negative symptoms and enhancing positive wellbeing, by teaching skills and techniques and enhancing positive emotions, harness and build on strengths, and maximise resilience over the longer-term. Choice, order, and length of modules (i.e., number of session over which they are completed) is tailored to the transdiagnostic difficulties of the individual using collaborative case formulation [44], although there are three core modules that everyone receives (outlined below). Treatment consists of weekly face-to-face one-hour sessions with the trial therapists. Sessions will involve collaboratively setting an agenda for the session based on the participants' ratings for their top 3 problems and top 3 strengths, the goals set for

For peer review only - http://bmjopen.bmj.com/site/about/guidelines.xhtml

therapy, and the module in focus. Participants will complete homework exercises to consolidate and practice the skills learned during the specific modules and will be strongly encouraged to continue this practice following the end of one module and move to the next.

The ten SHM modules (core modules listed in bold) are: (1) **Getting Acquainted with SHM**, (2) **Understanding Emotions**, (3) Managing and Tolerating Emotions, (4) Behavioural Activation, (5) Tackling Avoidance, (6) Tackling Unhelpful Thoughts, (7) Tackling Unhelpful Habits, (8) Overcoming Repetitive Thinking, (9) Managing Upsetting Memories and Images, and (10) **Relapse Prevention and Future Orientation**. Additional information about the content of the modules can be found in the supplementary materials (Supplementary Table 1).

Treatment-as-usual (TAU). For TAU, clinical psychologists and high-intensity CBT Therapists in teams specialising in CMHPs will be asked to provide the course of psychological therapy that they deem appropriate, in addition to referral to other health/social services and medication management. Psychological treatment in the specialist teams delivering TAU will standardly consist of disorder-focused Cognitive Behavioural Therapy, Eye Movement Desensitisation Reprocessing, or Behavioural Activation. The delivered treatment will be documented to ensure systematic understanding of the duration, frequency, and type of treatment administered.

Treatment Integrity. Therapists with experience in treating adult CMHPs will deliver the transdiagnostic intervention. Treatment fidelity and clinician adherence for the *Shaping Healthy Minds* group will be established using continued monitoring of completion of module components and through independent rating of specific treatment strategies by the supervising clinical psychologist. After every session, clinicians will complete a bespoke Treatment Fidelity Checklist which is a session-by-session self-report measure of compliance with the *Shaping Healthy Minds* approach, and these will be evaluated during weekly clinical

For peer review only

supervision with the trial clinical supervisor. In addition, a randomly-selected 25% of the audio-taped treatment sessions will be rated for adherence to the manuals by an experienced clinician, independent of the trial. Homework completion will be monitored by trial therapists.

This bespoke measure will be supplemented with The Cognitive Therapy Scale – Revised (CTS-R) – a standardised measure of competence within cognitive therapy, consisting of adherence to and skilful application of cognitive therapy methods and the therapeutic alliance [45]. The CTS-R has 13-items that are completed by an independent rater, assessing agenda setting, feedback, collaboration, pacing and efficient use of time, interpersonal effectiveness, charisma/flair, facilitation of emotional expression, guided discovery, conceptualisation, identifying key cognitions, application of cognitive change methods, application of behavioural techniques, use of homework.

Measures

Co-primary outcomes. The co-primary outcome measures are self-reported symptoms of depression and anxiety, indexed by the PHQ-9 [46], and GAD-7 [47], as well as levels of disability and functional impairment, indexed by the Work and Social Adjustment Scale (WSAS [48]).

Secondary outcomes. Given the transdiagnostic focus of the study, self-reported symptoms on specific disorders that client's meet criteria for at trial baseline will be indexed by the IAPT Phobia Scales (social phobia, agoraphobia, specific phobia, [49]), the Social Phobia Inventory (SPIN, [50]), the Penn State Worry Questionnaire (generalised anxiety, PSWQ, [51]), the Obsessive-Compulsive Inventory (OCI, [52]), the Revised Impact of Event Scale (posttraumatic stress, IES-R, [53]), the Agoraphobia-Mobility Inventory (MI, [54]), the Fear Questionnaire (specific phobias, FQ, [55]), the Panic Disorder Severity Scale- self report version (PDSS-SR, [56]), the Health Anxiety Inventory – short version (SHAI, [57]), and the Sheehan Disability Scale (SDS, [58]). Participants will only complete a selection of these

For peer review only

measures depending on their concerns and associated diagnoses. In addition to these disorder-specific measures, the Inventory of Depression and Anxiety Symptoms (IDAS-II) will capture both disorder-specific and transdiagnostic symptom dimensionality within a single measure [59]. Selection of these measures will be determined following completion of the structured clinical interview at the beginning of assessment.

Process Measures. We will also include a number of process-related measures which will be administered at baseline, at post-intervention, and at 3-month follow-up to begin to explore mechanisms of change and the feasibility of conducting embedded process outcome research within this type of trial (see Table 1). To explore the value of the individual modules administered within the transdiagnostic intervention, we will also administer module-relevant measures (e.g., rumination, distress tolerance) before and after completion of the module. Finally, participants' expectancy of treatment outcomes and measures of engagement and compliance will be administered at pre and post-intervention to inform the further development of the protocol.

Health economics measures. Data collection for the health economic evaluation will take a patient-level perspective [60, 61], recording the cost-per-session of treatment and productivity losses resulting from time off work as a consequence of their mental health difficulties. Data will be collected using the Healthlines Resource Use Questionnaire [60, 61], which is a measure of the participants use of health care services (including NHS, help at home), occupational productivity (i.e., time off work) and cost of transdiagnostic treatment delivery (e.g., direct and indirect time spent in service delivery). The Medical Outcomes Study Questionnaire Short Form 36 Health Survey (SF-36) [62], a generic quality of life questionnaire, will measure overall health and wellbeing, daily functioning, and general life satisfaction across multiple domains. These measures allow calculation of the additional number of quality of life years the treatment will yield. These data will allow preliminary

For peer review only

estimates of the potential cost-utility of the transdiagnostic intervention and also of the feasibility of acquiring these data within the trial protocol.

Methodological Aspects

Power analysis and sample size. Although a standard power calculation based on detecting treatment effects is the conventional approach to determining sample sizes for trials, the main aim of the current trial is to elucidate feasibility for a larger later-stage evaluation. We therefore sought at this stage only to provide a point-estimate of the effect of *Shaping Healthy Minds* to inform a power calculation for this putative fully powered later phase evaluation. Our previous experience with such early phase trial platforms indicates that 50 patients will provide sufficient numbers and diagnostic diversity to evaluate feasibility, acceptability, and procedural uncertainty for *Shaping Healthy Minds* and a plausible test of recruitment protocols. This will give 40 patients (20 per arm) at 3-month posttreatment follow up, assuming 20% attrition. This will provide a reasonable range of point estimates of effect on our set of candidate outcome measures sufficient to guide later phase trial work.

Data collection and confidentiality. Outcome data for all participants who are randomised will be collected via face-to-face interviews and written questionnaires at baseline, post-treatment, and 3-month follow-up. To maintain confidentiality all participants will be given a trial number so that personally identifying information is not linked to assessment or trial information. All data (including personally-identifiable information) will be stored on secure UK NHS databases, secure University of Cambridge servers and within locked filing cabinets under the management of the trial coordinator. Access will be limited to the immediate clinical research team.

Blinding. Outcome assessments will be conducted by independent raters who have no therapeutic relationship with the patients and are blind to treatment condition. Double blinding of patients and therapists is not possible due to the nature of the trial (i.e., a

For peer review only

psychological intervention). Unblinding will not be necessary because participants and therapists are not blinded to intervention allocation.

Statistical analysis plan. Initial analyses of the outcomes will be conducted by the trial statistician, blind to trial condition, following CONSORT standards (see Supplementary Figure File 2). There are no planned interim analyses. Initial analyses will be conducted on an intention-to-treat basis, with subsequent analyses being per protocol. Mixed model analyses of variance (ANOVAs) will be used to compare groups on outcomes at the three assessment points – baseline, post-intervention, and 3-month follow-up. Baseline levels on relevant measures will be included as covariates, as appropriate. Both intent-to-treat and per-protocol exploratory analyses will be conducted with our range of outcome measures following CONSORT standards. Multiple imputation will be used to account for missing data. Intent-to-treat analysis will also be used for those lost to attrition. Exploratory moderation and mediation analyses to examine process variables will be conducted using the MacArthur approach [63]. For the health economic data, costs associated with service use will be calculated by attaching a unit cost to each instance of use, and data will be combined with quality-adjusted life years (QALYs, [62]) derived from the SF-36 to arrive at a preliminary estimate of the cost-utility of the transdiagnostic intervention.

Monitoring and data management. The trial will take place at NHS sites and a research unit in the East of England. A Trial Management Group (TMG), will meet 1-2 times a year to: manage the protocol; monitor recruitment in relation to targets; deal with any adverse events; and co-ordinate the different stages of the project. The TMG consists of research clinical psychologists and assistant psychologists, a psychiatrist, a health economist, clinical psychology researchers, the trial statistician, a nurse practitioner, and a service user representative. Day-to-day project management will be the responsibility of a smaller trial team, meeting fortnightly to deal with administrative issues, troubleshooting, and recruitment

For peer review only

flow. Clinical supervision will take place fortnightly. As this is a Phase I/II trial, a data-management committee was deemed unnecessary, and as such the trial team are responsible for monitoring and data management. Data will be monitored for completeness and consistency using spot checks and plausibility checks carried out by the trial statistician. The trial lead, trial coordinator, and statistician will have full access to the final trial dataset. The study data will be reported in line with the current CONSORT recommendations.

Patient and Public Involvement

Most broadly, the driving force behind the development of *Shaping Healthy Minds* has been feedback from many hundreds of service users in clinical settings, specifically related to the suitability of treatments for complex and comorbid CMHPs. A Lived Experience Group comprising service users and carers hosted by the Cambridge Centre for Affective Disorders discussed the details of the current study at its meeting on 12 November 2013. This group provided useful feedback to the research team in terms of the materials for service-users (e.g., consent forms), the clinical setting for the intervention, and optimal forms of PPI involvement (specifically, the use of service-user researchers to conduct qualitative interviews with service users). Further, we received service-user input on the content of the draft treatment modules, and a part of this feasibility trial will be receiving feedback from participants on their experience of participating in the trial and using the manuals. We will send all participants a report describing the findings and their implications. We will also make participants aware of the study website. There will be a number of roles following completion of recruitment including the refinement and revision of the treatment manual along with involvement in the academic output preparation.

Ethics and Dissemination

Ethical approval and protocol amendments. This project has received ethical approval from the Health Research Authority of the UK National Health Service (East of

For peer review only

England; REC reference: 16/EE/0095). The study will be conducted within appropriate UK MRC, National Health Service and professional ethical guidelines, ensuring that Good Clinical Practice procedures are adhered to at all times. Protocol amendments will be circulated to the ethics committee, and trial team, and published in the online registration of the trial, and in the trial paper.

Safety aspects. Adverse events are managed in line with UK MRC protocols and, in the unlikely case of an adverse event, will be documented appropriately. Precautions have been taken to reduce the likelihood of adverse events occurring; for example, patients who are acutely suicidal or at high risk of harm do not meet study inclusion criteria. The interventions are delivered by therapists experienced in the management of risk and in the treatment of psychological disorders. In the case of any adverse events as a result of the intervention that would interfere with participation, participation in the trial will be discontinued. Regular team meetings will be conducted to monitor any difficulties patients may be having and ways of best dealing with these difficulties. Serious adverse events will be reported to the Ethics Committee. The trial is underwritten by the University of Cambridge in the case that any individual suffers harm or requires post-trial care. Any adverse events will be reported in the trial paper.

Dissemination policy. There are no publication restrictions and findings will be disseminated broadly to participants, healthcare professionals, the public, and other relevant groups. Academic outputs will take the form of peer-reviewed empirical journal articles, commentary pieces, and conference presentations. Clinical outputs will be prioritised by the research team in order to maximise the impact of the findings with practitioners and commissioners. Outputs will comprise clinical conferences, workshops, service user groups, and a study website that will make the intervention materials and related measures generally available. We will send all participants a report describing the findings and their implications.

For peer review only

We will also make participants aware of the study website. We anticipate that trial findings will inform future revisions of clinical guidelines for numerous forms of mood, anxiety, and stress disorders, and the development of guidelines for comorbid conditions. Anonymised data from the trial will be made publicly available on an open-access database.

Discussion

A significant proportion of the cost of CMHPs is generated by adults suffering from complex and comorbid depression, stress and anxiety, where treatment non-response, cross-sector service-use across health, social care, and housing, and loss of productivity are greatest. Providing effective interventions for these mental health problems therefore has the potential to reduce both long-term treatment costs as well as prevent large productivity losses. At present, most psychological interventions focus on specific diagnoses and many treatment manuals take a 'one-size-fits-all' approach. Current evidence-based interventions only achieve clinical recovery for 40-70% of patients, with people suffering complex co-morbid conditions faring significantly worse. This randomised controlled feasibility trial aims to pave the way for a scaled-up efficacy trial of a new transdiagnostic modular treatment for all CMHPs – *Shaping Healthy Minds* – that enables the flexible delivery of evidence-based techniques. This treatment approach may improve the effectiveness and dissemination of evidence-based intervention for the many individuals for whom diagnosis-specific treatments leave significant difficulties unaddressed. The results from this trial will provide a range of estimates of effect sizes that can be used to power a later stage trial of treatment efficacy, to refine the treatment protocol, and to inform future evaluation of the mechanisms underlying any treatment effects. If effective, *Shaping Healthy Minds* has the potential to improve outcome for those with complex presentations, through offering a cost-effective treatment option to reduce chronic, transdiagnostic psychological difficulties.

For peer review only

Trial Status: This trial was registered at clinicaltrials.gov on 4 May 2017 (NCT03143634). This article was submitted on 31 May 2018. To date, 15 participants have met eligibility criteria for the study and have been randomised to a condition. The trial opened on 31 July 2017, and data collection aims to be completed by September 2019.

Figure 1. Participant flow diagram for the HARMONIC Trial with anticipated participant numbers at each stage

For peer review only

List of Abbreviations

DSM – Diagnostic and Statistical manual

IAPT – Improving Access to Psychological Therapies

HARMONIC – The Healthy and Resilient Mind Programme: Building Block for Mental Wellbeing

MRC – Medical Research Council

NHS – National Health Service

PTSD – Posttraumatic Stress Disorder

RCT – Randomised Controlled Trial

SCID-5 – Structured Clinical Interview for DSM-5 disorders

TAU – Treatment-as-usual

CMHP – Common Mental Health Problems

NICE – National Institute for Health and Care Excellence

CBT – Cognitive Behaviour Therapy

ACT – Acceptance and Commitment Therapy

DBT – Dialectical Behaviour Therapy

MBCT – Mindfulness Based Cognitive Therapy

For peer review only

Authors' contributions

MB will manage the trial, deliver the intervention, co-develop the treatment manuals and helped to draft the manuscript; CH will assist with trial management and helped to draft the manuscript; AB advises on the delivery of the interventions and provided guidance on the protocol; JC provides practical and NHS service support for the trial; COL, RE and DJ provides support for the trial management; PW is the trial statistician responsible for the randomisation, advise on analysis strategy, and analysis of the data; LL provides support for the health economics component; SR provides service-user support for the trial; SG and WK advised on the treatment manual; JN co-developed and adapted the treatment manuals; TD designed the study, co-developed and adapted the treatment manuals, and helped to draft the manuscript. All authors read and approved the final manuscript.

Authors' information

Melissa Black:	melissa.black@mrc-cbu.cam.ac.uk
Caitlin Hitchcock:	caitlin.hitchcock@mrc-cbu.cam.ac.uk
Anna Bevan:	anna.bevan@mrc-cbu.cam.ac.uk
Cliodhna O Leary:	cliodhna.oleary@cpft.nhs.uk
James Clarke:	james.clarke@cpft.nhs.uk
Rachel Elliott:	rachel.elliott@cpft.nhs.uk
Peter Watson:	peter.watson@mrc-cbu.cam.ac.uk
Louise Lafortune:	ll394@medschl.cam.ac.uk
Sarah Rae:	sarah.rae@rockyroad.org.uk
Simon Gilbody:	simon.gilbody@york.ac.uk
Willem Kuyken:	willem.kuyken@psych.ox.ac.uk
David Johnston:	david.johnston@mrc-cbu.cam.ac.uk
Jill Newby:	j.newby@unsw.edu.au
Tim Dalgleish:	tim.dalgleish@mrc-cbu.cam.ac.uk

For peer review only - http://bmjopen.bmj.com/site/about/guidelines.xhtml

Funding

This paper presents independent research funded by the National Institute of Health Research (NIHR) under its Research for Patient Benefit (RfPB) Programme (Grant Reference Number PB-PG-0214-33072) grant awarded to Tim Dalgleish, Peter Watson, Jill Newby, Leonora Brosan, Rajini Ramana, Louise Lafortune, Sarah Rae, Simon Gilbody, Willem Kuyken, and Caitlin Hitchcock. Jill Newby is supported by a NHMRC/MRFF Fellowship (1145382). Please note that the funding body does not have authority over the running of the trial – all decisions rest with the trial team. The views expressed are those of the author(s) and not necessarily those of the NHS, the NIHR or the Department of Health and Social Care.

Competing Interests

The authors have no competing interests.

Patient consent

Obtained.

Ethics approval

NHS National Research Ethics Committee (East of England, Reference: 16/EE/0095)

Sponsor Information

University of Cambridge School of Clinical Medicine
Carolyn Read (Research Governance Officer)
Cad50@medschl.cam.ac.uk
Box 111 Cambridge Biomedical Campus
Cambridge, CB2 0SP
United Kingdom

For peer review only

References

1. Kessler, R.C., et al., The epidemiology of major depressive disorder: results from the National comorbidity survey replication (NCS-R). Journal of the American Medical Association, 2003. 289: p. 3095e3105.
2. Medical Research Council, Review of mental health research: Report of the strategic review group. 2010, Medical Research Council: London.
3. Pilling, S., et al., Identification and care pathways for common mental health disorders: summary of NICE guidance. BMJ, 2011. 342: p. d2868.
4. Brown, T.A., et al., Current and lifetime comorbidity of the DSM-IV anxiety and mood disorders in a large clinical sample. Journal of Abnormal Psychology, 2001. 110(4): p. 585-599.
5. Kessler, R.C., et al., Lifetime prevalence and age-of-onset distributions of DSM-IV disorders in the national comorbidity survey replication. . Archives of General Psychiatry, 2005. 62(6): p. 593-602.
6. Moses, E.B. and D.H. Barlow, A new unified treatment approach for emotional disorders based on emotion science. Current Directions and Psychological Science, 2006. 15(3): p. 146-150.
7. Bockting, C.L., et al., A lifetime approach to major depressive disorder: The contributions of psychological interventions in preventing relapse and recurrence. Clinical Psychology Review, 2015. 41: p. 16-26.
8. Loerinc, A.G., et al., Response rates for CBT for anxiety disorders: Need for standardized criteria. Clinical Psychology Review, 2015. 42: p. 72-82.
9. Ellard, K.K., et al., Unified Protocol for the Transdiagnostic Treatment of Emotional Disorders: Protocol Development and Initial Outcome Data. Cognitive and Behavioral Practice, 2010. 17: p. 88-101.
10. Newby, J.M., et al., Systematic review and meta-analysis of transdiagnostic psychological treatments for anxiety and depressive disorders in adulthood. Clin Psychol Rev, 2015. 40: p. 91-110.
11. Craske, M.G., Transdiagnostic Treatment for Anxiety and Depression. Depression and Anxiety, 2012. 29: p. 749-753.
12. Brown, T.A. and D.H. Barlow, A proposal for a dimensional classification system based on the shared features of the DSM-IV anxiety and mood disorders: implications for assessment and treatment. Psychol Assess, 2009. 21(3): p. 256-71.
13. Harvey, A., G., et al., eds. Cognitive behavioural processes across psychological disorders: A transdiagnostic approach to research and treatment. 2004, Oxford University Press: Oxford.
14. Barlow, D.H., L.B. Allen, and M.L. Choate, Toward a Unified Treatment for Emotional Disorders. Behavior Therapy, 2004. 35: p. 205-230.
15. Norton, P.J., S.A. Hayes, and D.A. Hope, Effects of a transdiagnostic group treatment for anxiety on secondary depression. Depress Anxiety, 2004. 20(4): p. 198-202.
16. Richards, D.A., et al., Cost and Outcome of Behavioural Activation versus Cognitive Behavioural Therapy for Depression (COBRA): a randomised, controlled, non-inferiority trial. The Lancet, 2016. 388(10047): p. 871-880.

17. Borkovec, T.D. and E. Costello, Efficacy of applied relaxation and cognitive-behavioral therapy in the treatment of Generalised Anxiety Disorder. Journal of Consulting and Clinical Psychology, 1993. 61: p. 611-619.
18. Dugas, M.J., et al., Group Cognitive–Behavioral Therapy for Generalized Anxiety Disorder: Treatment Outcome and Long-Term Follow-Up. Journal of Consulting and Clinical Psychology, 2003. 71: p. 821-825.
19. Clark, D.M., et al., Cognitive therapy versus exposure and applied relaxation in social phobia: A randomized controlled trial. J Consult Clin Psychol, 2006. 74(3): p. 568-78.
20. Bryant, R.A., et al., Imaginal exposure alone and imaginal exposure with cognitive restructuring in treatment of posttraumatic stress disorder. Journal of Consulting and Clinical Psychology, 2003. 71(4): p. 706-712.
21. Chorpita, B.F. and E.L. Daleiden, Mapping evidence-based treatments for children and adolescents: application of the distillation and matching model to 615 treatments from 322 randomized trials. Journal of Consulting and Clinical Psychology, 2009. 77(3): p. 566-79.
22. Chorpita, B.F., E.L. Daleiden, and J.R. Weisz, Modularity in the design and application of therapeutic interventions. Applied and Preventive Psychology, 2005. 11(3): p. 141-156.
23. Barlow, D.H., et al., The Unified Protocol for Transdiagnostic Treatment of Emotional Disorders Compared With Diagnosis-Specific Protocols for Anxiety Disorders: A Randomized Clinical Trial. JAMA Psychiatry, 2017.
24. Gros. D.F. Development and initial evaluation of Transdiagnostic Behavior Therapy (TBT) for veterans with affective disorders. Psychiatry Research, 2014, 220, 275-282.
25. Riccardi, C.J., Korte, K.J. and Schmidt, N.B. False Safety Behavior Elimination Therapy: A randomized study of a brief individual transdiagnostic treatment for anxiety disorders. Journal of Anxiety Disorders, 46, 35-45.
26. Wilson, G.T., Manual-based treatments: The clinical application of research findings. Behaviour Research and Therapy, 1996. 34(4): p. 295-314.
27. Craig, P., et al., Developing and evaluating complex interventions: The new Medical Research Council guidance. British Medical Journal, 2008. 337.
28. Reinholt, N. and J. Krogh, Efficacy of transdiagnostic cognitive behaviour therapy for anxiety disorders: a systematic review and meta-analysis of published outcome studies. Cognitive Behaviour Therapy 2014. 43: p. 171-184.
29. American Psychiatric Association. Diagnostic and Statistical Manual of Mental Disorders. Fifth ed. 2013, Arlington, VA: American Psychiatric Publishing.
30. First, M.B., et al., Structured Clinical Interview for DSM-5 - research Version (SCID-5 for DSM-5, Research Version, SCID5-RV, Version 1.0.0). 2015, Arlington, VA: American Psychiatric Association.
31. Beck, J., Cognitive Behavior Therapy: Basics and Beyond (Second Edition). 2011, New York: Guilford Press.
32. Hayes, S.C., K.D. Strosahl, and K.G. Wilson, Acceptance and commitment therapy: An experiential approach to behavior change. . 1999, New York: Guilford Press.
33. Linehan, M.M., Cognitive-Behavioral Treatment of Borderline Personality Disorder. 1993, New York: Guilford Press.
34. Segal, Z.V., J.M.G. Williams, and J.D. Teasdale, Mindfulness-based Cognitive Therapy for Depression: A new approach to preventing relapse. 2002, New York: Guilford Press.

35. Martell, C.R., S. Dimidjian, and H.-D. Ruth, Behavioral Acitvation for Depression: A Clinician's Guide. 2010, New York: Guilford Press.
36. Ekers, D., et al., Behavioural Activation for Depression; An update of meta-analysis of effectiveness and subgroup analysis. PLoS ONE, 2014. 6(6).
37. Bennett-Levy, J., et al., Oxford Guide to Behavioural Experiments in Cognitive Therapy. 2004, Oxford: Oxford University Press.
38. Norton, P.J., Group Cognitive-Behavioural Therapy of Anxiety. 2012, New York: Guilford Press.
39. Harris, R., ACT Made Simple: A quick start guide to ACT basics and beyond. 2009, Oakland, CA: New Harbinger Publications, Inc.
40. Kuyken, W., et al., How does mindfulness-based cognitive therapy work? Behaviour Research and Therapy, 2010. 48(11): p. 1105-1112.
41. Khoury, B., et al., Mindfulness-based therapy: A comprehensive meta-analysis. Clinical Psychology Review, 2013. 33(6): p. 763-771.
42. Dimidjian, S., et al., Considering Meta-Analysis, Meaning, and Metaphor: A Systematic Review and Critical Examination of "Third Wave" Cognitive and Behavioral Therapies. Behavior Therapy, 2016. 47: p. 886-905.
43. Cuijpers, P., et al., How effective are cognitive behavior therapies for major depressionand anxiety disorders? A meta-analytic update of the evidence. World Psychiatry, 2016. 15: p. 245-258.
44. Kuyken, W., C.A. Padesky, and R. Dudley, Collaborative Case Conceptualisation: Working Effectively with Clients in Cognitive-Behavioral Therapy. 2009, New York, NY. : The Guilford Press.
45. Blackburn, I.-M., et al., The Revised Cognitive Therapy Scale (Cts-R): Psychometric Properties. Behavioural and Cognitive Psychotherapy, 2001. 29(04).
46. Kroenke, K., R.L. Spitzer, and J.B.W. Williams, The PHQ-9: Validity of a Brief Depression Severity Measure. Journal of General Internal Medicine, 2001. 16(9): p. 606-613.
47. Spitzer, R.L., et al., A brief measure for assessing generalized anxiety disorder: The GAD-7. Archives of Internal Medicine, 2006. 166: p. 1092-1097.
48. Mundt, J.C., et al., The Work and Social Adjustment Scale: a simple measure of impairment in functioning. British Journal of Psychiatry, 2002. 180(5): p. 461-464.
49. IAPT, D.o.H., The IAPT data handbook version 2.0.1. 2011: Published to DH Website, in electronic PDF format only. .
50. Connor, K.M., et al., Psychometric properties of the Social Phobia Inventory (SPIN): New self-rating scale. British Journal of Psychiatry, 2000. 176: p. 379-386.
51. Meyer, T.J., et al., Development and validation of the Penn State Worry Questionnaire. Behaviour Research and Therapy 1990. 28(6): p. 487-495.
52. Foa, E.B., et al., The validation of a new obsessive-compulsive disorder scale: The Obsessive-Compulsive Inventory. Psychological Assessment, 1998. 10(3): p. 206-214.
53. Weiss, D.S., The Impact of Event Scale: Revised, in Cross-cultural assessment of psychological trauma and PTSD, J.P. Wilson and C.S. Tang, Editors. 2007, Springer: New York. p. 219-238.
54. Chambless, D.L., et al., The Mobility Inventory for Agoraphobia. Behaviour Research and Therapy, 1985. 23(1): p. 35-44.

55. Marks, I.M. and A.M. Mathews, Brief standard self-rating for phobic patients. Behavior Research and Therapy, 1979. 17: p. 263-267.
56. Shear, M.K., et al., Multicenter collaborative panic disorder severity scale. American Journal of Psychiatry, 1997. 154(11): p. 1571-1575.
57. Salkovskis, P.M., et al., The Health Anxiety Inventory: development and validation of scales for the measurement of health anxiety and hypochondriasis. Psychological Medicine, 2002. 32(5): p. 843-853.
58. Sheehan, D.V., The Anxiety Disease. 1983, New York: Charles Scribner and Sons.
59. Watson, D., et al., Development and Validation of New Anxiety and Bipolar Symptom Scales for an expanded version of the IDAS (the IDAS-II). Assessment, 2012. 19(4): p. 399-420.
60. Dixon, P., et al., Cost-effectiveness of telehealth for patients with depression: evidence from the Healthlines randomised controlled trial. British Journal of Psychiatry Open, 2016. 1: p. 262-269.
61. Salisbury, C., et al., Effectiveness of an integrated telehealth service for patients with depression: a pragmatic randomised controlled trial of a complex intervention. Lancet Psychiatry, 2016. 3: p. 515-525.
62. Ware, J.E. and C.D. Sherbourne, The MOS 36-item short-form health survey (SF-36). I. Conceptual framework and item selection. Medical Care, 1992. 30(6): p. 473-483.
63. Kraemer, H.C., et al., How and Why Criteria Defining Moderators and Mediators Differ Between the Baron & Kenny and MacArthur Approaches. Health Psychology, 2008. 27: p. S101-S108.
64. Devilly, G.J. and T.D. Borkovec, Psychometric properties of the credibility/expectancy questionnaire. Journal of Behavior Therapy and Experimental Psychiatry, 2000. 31(2): p. 73-86.
65. Garnefski, N., V. Kraaij, and P. Spinhoven, Negative life events, cognitive emotion regulation and emotional problems. Personality and Individual Differences, 2001. 30: p. 1311-1327.
66. Fresco, D.M., et al., Initial psychometric properties of the Experiences Questionnaire: Validation of a self-report measure of decentering. Behavior Therapy, 2007. 38: p. 234-246.
67. Izard, C.E., et al., Stability of emotion experiences and their relations to traits of personality. Journal of Personality and Social Psychology, 1993. 64(5): p. 847-860.
68. Morey, L.C., Development and initial evaluation of a self-report form of the DSM-5 Level of Personality Functioning Scale. Psychological Assessment, 2017. 29(10): p. 1302-1308.
69. Treynor, W., R. Gonzalez, and S. Nolen-Hoeksema, Rumination Reconsidered: A Psychometric Analysis. Cognitive Therapy and Research 2003. 27(3): p. 247-259.
70. Simons, J.S. and R.M. Gaher, The Distress Tolerance Scale: Development and Validation of a Self-Report Measure. Motivation and Emotion, 2005. 29(2): p. 83-102.
71. Gratz, K.L. and L. Roemer, Multidimensional Assessment of Emotion Regulation and Dysregulation: Development, Factor Structure, and Initial Validation of the Difficulties in Emotion Regulation Scale. Journal of Psychopathology and Behavioral Assessment, 2004. 26(1): p. 41-54.
72. Weissman, A.N., The Dysfunctional Attitude Scale: A Validation Study. 1979, University of Pennsylvania.

73. Baer, R.A., G.T. Smith, and K.B. Allen, Assessment of mindfulness by self-report: the Kentucky inventory of mindfulness skills. Assessment, 2004. 11(3): p. 191-206.
74. Reiss, S., et al., Anxiety sensitivity, anxiety frequency and the prediction of fearfulness. Behaviour Research and Therapy, 1986. 24(1): p. 1-8.
75. Foa, E.B., et al., The Posttraumatic Cognitions Inventory (PTCI): Development and validation. Psychological Assessment, 1999. 11(3): p. 303-314.
76. Jarrett, R.B., et al., Skills of Cognitive Therapy (SoCT): A New Measure of Patients' Comprehension and Use. Psychological Assessment, 2011. 23(3): p. 578-586.
77. Gámez, W., et al., Development of a measure of experiential avoidance: the Multidimensional Experiential Avoidance Questionnaire. Psychological Assessment, 2011. 23(3): p. 692-713.

For peer review only

Table 1.

Additional Outcome and Process measures to assess changes in potential mechanisms of psychological distress and in response to specific transdiagnostic intervention modules.

**Measure**	**Focus area**
The Treatment Credibility / Expectancy Questionnaire (CEQ) [64]	Expectancy about treatment outcome, as well as the credibility of the treatment.
Cognitive Emotion Regulation Questionnaire (CERQ) [65]	Ability to contextualize negative events within a wider frame of reference.
The Experiences Questionnaire (EQ) [66]	Ability to disengage from troublesome mental content and take a more accepting stance towards it, as well as the tendency to engage in rumination.
Differential Emotions Scale (DES [67])	Intensity with which they experience different emotions on a typical day to obtain summary scores for positive emotions, negative emotions, and denied emotions (the number of emotions *not* endorsed by the participant).
Levels of Personality Functioning Scale (LPFS) [68]	Personality functioning based on the DSM-5 Alternative Model of Personality Disorders. It has four subscales: Identity, Self-Direction, Empathy, and Intimacy.
Ruminative Responses Scale of the Response Styles Questionnaire (RRS) [69]	Rumination (Module 7 – Overcoming Repetitive Thinking)
Distress Tolerance Scale (DTS)[70]	Ability to tolerate distress (Module 3 – Managing and Tolerating Emotions)
Difficulties with Emotion Regulation Scale (DERS) [71]	Ability to label, perceive, and regulate emotions (Modules 2 – Understanding Emotions and 3 – Managing and Tolerating Emotions)
Dysfunctional Attitudes Scale (DAS) short form (version 1 and 2) [72]	Negative beliefs, thoughts and assumptions (Module 6 – Tackling Unhelpful Thoughts)

For peer review only

Kentucky Inventory of Mindfulness Skills (KIMS) [73]	Mindful awareness (Module 2 – Understanding Emotions)
Anxiety Sensitivity Index (ASI) [74]	Fear of physical anxiety sensations (Module 3 – Managing and Tolerating Emotions and Module 5 – Tackling Avoidance)
Posttraumatic cognitions inventory – short version (PTCI) [75]	Trauma-related beliefs and maladaptive appraisals of intrusive symptoms (Module 9 – Managing Upsetting Memories and Images)
Skills of Cognitive Therapy (SoCT) [76]	Implementation of cognitive therapy skills (Module 6 – Tackling Unhelpful Thoughts)
The Multidimensional Experiential Avoidance Questionnaire [77]	Avoidance of internal experiences including thoughts, feelings, physical sensations (Module 5 – Tackling Avoidance)

For peer review only

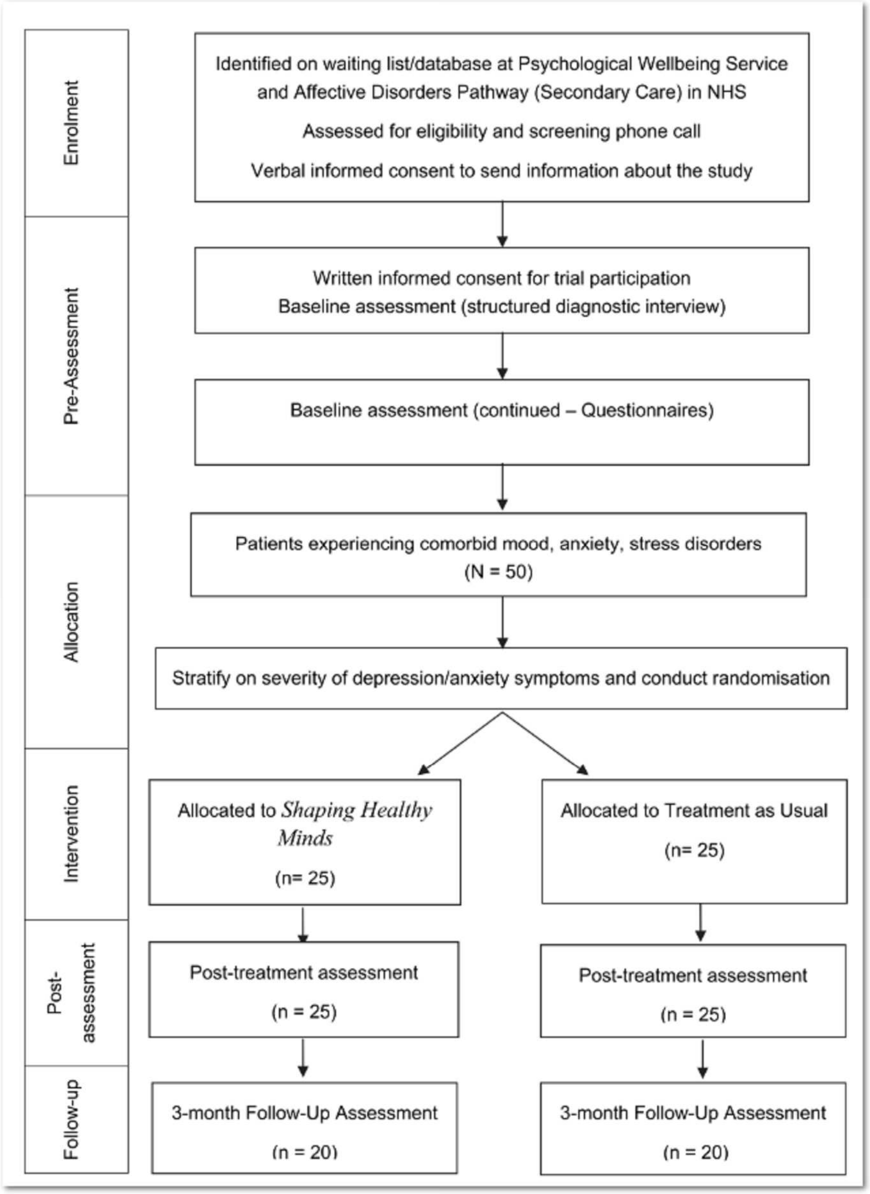

Figure 1. Participant flow diagram for the HARMONIC Trial with anticipated participant numbers at each stage

CAMBRIDGE CLINICAL RESEARCH CENTRE FOR AFFECTIVE DISORDERS

Cambridgeshire and Peterborough NHS Foundation Trust

MRC Cognition and Brain Sciences Unit

Participant Information Sheet
Study title: *Shaping Healthy Minds*

We invite you to participate in a study investigating a new modular treatment for mental health difficulties. Please read this information sheet if you wish to hear about the study in more detail. Your participation is *entirely voluntary*. Your treatment through the NHS will not be affected if you decide not to participate in this research.

This treatment – *Shaping Healthy Minds* (SHM) – treats symptoms that are common across depression and multiple anxiety disorders. The treatment consists of components of the best available evidence-based psychological treatments (e.g., mindfulness-based interventions, Acceptance and Commitment Therapy, Cognitive Behavioural Therapy, Behavioural Activation, and Dialectical Behaviour Therapy). Through using components of different types of therapy, the SHM aims to not only treat the principle problem that each individual is experiencing (e.g., depression or anxiety) but to target other psychological symptoms the individual is experiencing at the same time. In this way, someone who is experiencing both depression and anxiety will receive treatment for both of these issues, in contrast to the usual approach of treating one problem first, using only one type of therapy.

Purpose of the study

The purpose of this study is test whether the SHM is an effective treatment for people experiencing multiple psychological difficulties. We are interested in whether SHM will reduce symptoms of multiple mental health issues in those who are suffering from both depression and one or more anxiety disorders.

What's involved?

If you decide to take part, you would be asked to attend a 'screening' session to determine whether you are eligible to participate in this study. It will take 60-90 minutes and involves interviews and questionnaires focusing on emotions and mental health.

If you are eligible to participate in the trial you will then be randomly allocated to one of two groups:

(i) 15-20 sessions of the *Shaping Healthy Minds* (SHM) programme,
(ii) Treatment you would usually receive from the NHS (Treatment-as-Usual)

You will not be able to choose which of these groups you are allocated to, as the allocation is random and decided by a computer.

In the SHM programme, you will complete individual sessions with a clinical psychologist. The exact therapy components that you complete will be selected in consultation with you and based on the particular symptoms you are experiencing. That is, the treatment will be shaped for you. You will also receive the regular NHS care that you normally receive except for any psychological therapy.

In Treatment-as-Usual, you will receive all of the regular NHS services you would normally receive including any psychological therapy.

In addition to receiving treatment sessions, you will be asked to attend five assessment sessions where you will be asked to complete some questionnaires, some computer-based tasks that ask about your memories and emotions, including a task that involves pleasant and unpleasant images. In addition, there is an **optional** neuroimaging task, involving two functional magnetic resonance imaging scans (before and after treatment).

For peer review only - http://bmjopen.bmj.com/site/about/guidelines.xhtml

You can choose not to complete the imaging sessions and still participate in the study.

Prior to completing the treatment sessions (SHM or Treatment-as-Usual) which form the main part of the study, you will be asked to complete one assessment session which involves interviews and questionnaires, and one assessment session with computer tasks and the optional imaging session. You would then repeat these two assessment sessions after completing the treatment, followed by one final assessment session 3 months following the completion of treatment. Each assessment session will take approximately one and a half hours, which includes a break if needed. With your consent, some sessions will be audio-recorded to allow checks on the quality of the treatment you are receiving.

Why have I been invited to take part?
All individuals currently receiving NHS services for depression or anxiety in the Cambridge area are being invited to participate in this study.

Do you have to take part?
No, it is up to you to decide. We will describe the study and go through this information sheet, which we will then give to you. If you do want to join in we'll ask you to sign a consent form, a copy of which you can keep along with this information sheet. You are free to withdraw from the study at any point *without giving us a reason*. You will not be treated any differently by any NHS service if you choose not to participate in this study or if you decide to withdraw.

Will I be reimbursed?
You will be reimbursed for the assessment sessions at a rate of £6 per hour for your time. It is anticipated that the one screening session and four assessment sessions will not exceed a total of ten hours, for which you would receive a minimum of £45, plus travel costs. If you choose to do the neuroimaging sessions as well, you will receive £20 per imaging session, so you would receive a total of £85. You will not be reimbursed for the therapy sessions as you will be receiving therapy free of charge by qualified clinical psychologists.

Are there any risks or benefits associated with taking part?
All of the tasks, interviews, and questionnaires we will ask you to complete have been used safely in previous research. As with any research involving emotional material, there is a chance that you will experience some upset when you discuss personal memories and difficulties. In our experience this is usually very mild and short-lived with no lasting ill effects. After participation you will receive a complete and thorough explanation of the study and you will be encouraged to express your feelings about your experience, if you wish. Additionally, you will be able to contact a member of the research team (a qualified clinical psychologist) after any session should you feel you are experiencing distress as a result of taking part in the study.

What are the possible benefits of taking part?
We hope that the findings from this research will lead to improvement in treatment options for people experiencing multiple mental health disorders. We also hope that completing the SHM, if you are randomly allocated to that treatment, will have a beneficial impact on your psychological difficulties. We cannot guarantee that this will be the case for everybody who takes part, but you will have an opportunity to experience a range of therapeutic strategies and techniques.

What if there is a problem?
For any complaint about the way you have been dealt with during the study or any possible harm you may have suffered you can contact the Patient Advice and Liaison Service on 01223 726 774, http://www.cpft.nhs.uk/about-us/pals.htm

Further Information & Contact Details:
If you would like any further information about this project please contact the research coordinator **Dr Melissa Black** at the MRC Cognition and Brain Sciences Unit (Tel: **01223 273 739**; Email: **melissa.black@mrc-cbu.cam.ac.uk**)

Thank you for reading this information sheet

For peer review only - http://bmjopen.bmj.com/site/about/guidelines.xhtml

Participant ID Number:

Participant Consent Form (Version 2.2, April 2018)

Title of the project: Shaping Healthy Minds

Name of project coordinator: Dr Melissa Black

Please initial box:

1. I confirm that I have read and understood the Information Sheet for the above study. I have had the opportunity to consider the information, ask questions and have had these answered satisfactorily.

2. I understand that my participation is voluntary and that I am free to withdraw at any time without giving any reason and without my medical care or legal rights being affected.

3. I understand that data (including audio recordings) collected from me during the study may be looked at by individuals from the research team where it is relevant to my taking part in this research. I give permission for these individuals to have access to these data.

4. I understand that my GP will be informed of my participation in this study if s/he is not already aware of it.

5. I agree to take part in the above study.

6. I agree to a researcher contacting me after the end of this study about possible future research (optional).

……………………………………………… …………..   …………………………..

Name of Participant   Date   Signature

……………………………………………… …………..   ………………… ………

Name of Person taking consent   Date   Signature

(When the form is completed, 1 copy is for the participant and 1 is for the study files)

For peer review only - http://bmjopen.bmj.com/site/about/guidelines.xhtml

HARMONIC Trial (Healthy and Resilient Mind Programme: Building Blocks for Mental Wellbeing)

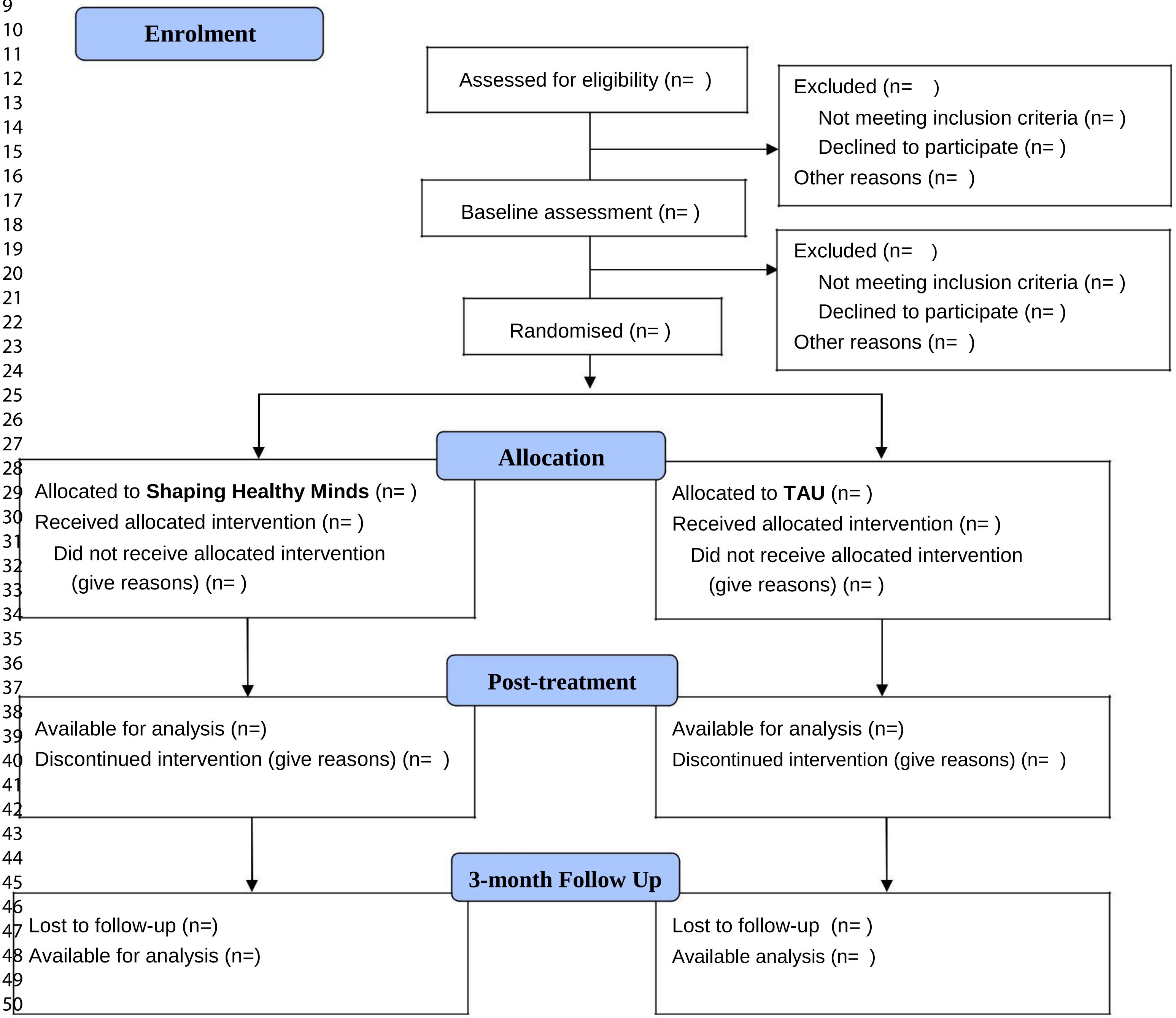

Figure 1. CONsolidated Standards of Reporting Trials (CONSORT) diagram

Supplementary Table 1.

Key treatment components of Shaping Healthy Minds Core and Optional Modules.

#	Module Title	Sections of Module	Duration (sessions)
1	Getting Acquainted with *Shaping Healthy Minds*<br>*Core Module*	• Education about depression and anxiety, and about emotions (Including 5-part model of emotion episodes)<br>• Orientation to treatment<br>• Defining Top 3 Problems and Top 3 Strengths<br>• Setting treatment goals and making plans<br>• Enhancing motivation	1-3
2	Understanding emotions<br>*Core Module*	• Education about emotions<br>• Orientation to the emotion model<br>• Self-monitoring of emotion episodes<br>• Introduction to Mindful Awareness of Emotions	1-2
3	Managing and Tolerating Emotions<br>*Optional Module*	• Education about emotion management toolbox<br>• Relaxation<br>• Exercise<br>• Social Support<br>• Distraction<br>• Self-soothing<br>• Accepting and Tolerating Feelings	1-3
4	Behavioural Activation<br>*Optional Module*	• Education about low activity cycle<br>• Activity Monitoring<br>• Activity Scheduling and establishing positive routines<br>• Savouring the good things	1-2
5	Tackling Avoidance<br>*Optional Module*	• Education about avoidance<br>• In vivo exposure<br>• Interoceptive exposure<br>• Emotion exposures	1-3
6	Tackling unhelpful thoughts<br>*Optional Module*	• Education about thoughts<br>• Thought monitoring<br>• Thought questioning and cognitive flexibility<br>• Behavioural experiments<br>• Accepting and tolerating thoughts	1-3

For peer review only

7	Tackling unhelpful habits *Optional Module*	• Education about habits<br>• Self-monitoring of habits<br>• Step-by-step habit change process	1-3
8	Overcoming Repetitive Thinking *Optional Module*	• Education about rumination and worry<br>• Self-monitoring of rumination and worry<br>• Getting unstuck toolbox<br>• Shifting thinking styles and perspective, directing attention	1-4
9	Managing upsetting memories and images *Optional Module*	• Education about memories and intrusive images<br>• Monitoring of memories<br>• Imaginal exposure<br>• Rescripting memories<br>• Reliving + rescripting memories	2-4
10	Preventing relapse and building a positive future *Core Module*	• Education about lapses and relapses<br>• Evaluating treatment progress and consolidating learning<br>• Creating a therapy blueprint (relapse prevention plan)<br>• Setting goals and making future plans	1

For peer review only

SPIRIT
STANDARD PROTOCOL ITEMS: RECOMMENDATIONS FOR INTERVENTIONAL TRIALS

SPIRIT 2013 Checklist: Recommended items to address in a clinical trial protocol and related documents*

Section/item	Item No	Description	Page/Line Reference
**Administrative information**			
Title	1	Descriptive title identifying the study design, population, interventions, and, if applicable, trial acronym	p. 1, line 1
Trial registration	2a	Trial identifier and registry name. If not yet registered, name of intended registry	p. 1, line 28
	2b	All items from the World Health Organization Trial Registration Data Set	See attached
Protocol version	3	Date and version identifier	p. 1, line 29
Funding	4	Sources and types of financial, material, and other support	p. 1, line 30<br>p. 22, line 1
Roles and responsibilities	5a	Names, affiliations, and roles of protocol contributors	p. 1, line 4
	5b	Name and contact information for the trial sponsor	p. 22, line 20
	5c	Role of study sponsor and funders, if any, in study design; collection, management, analysis, and interpretation of data; writing of the report; and the decision to submit the report for publication, including whether they will have ultimate authority over any of these activities	p. 22, line 1
	5d	Composition, roles, and responsibilities of the coordinating centre, steering committee, endpoint adjudication committee, data management team, and other individuals or groups overseeing the trial, if applicable (see Item 21a for data monitoring committee)	p. 16, line 16
**Introduction**			

For peer review only

Background and rationale	6a	Description of research question and justification for undertaking the trial, including summary of relevant studies (published and unpublished) examining benefits and harms for each intervention	p. 4, line 4
	6b	Explanation for choice of comparators	p. 8, line 6
Objectives	7	Specific objectives or hypotheses	p. 8, line 9
Trial design	8	Description of trial design including type of trial (eg, parallel group, crossover, factorial, single group), allocation ratio, and framework (eg, superiority, equivalence, noninferiority, exploratory)	p. 8, line 23

Methods: Participants, interventions, and outcomes

Study setting	9	Description of study settings (eg, community clinic, academic hospital) and list of countries where data will be collected. Reference to where list of study sites can be obtained	p. 9, line 23
Eligibility criteria	10	Inclusion and exclusion criteria for participants. If applicable, eligibility criteria for study centres and individuals who will perform the interventions (eg, surgeons, psychotherapists)	p. 9, line 4<br>p. 9, line 12<br>p. 12, line 15
Intervention s	11a	Interventions for each group with sufficient detail to allow replication, including how and when they will be administered	p. 10, line 21
	11b	Criteria for discontinuing or modifying allocated interventions for a given trial participant (eg, drug dose change in response to harms, participant request, or improving/worsening disease)	p. 10, line 17
	11c	Strategies to improve adherence to intervention protocols, and any procedures for monitoring adherence (eg, drug tablet return, laboratory tests)	p. 12, line 15<br>p. 17, line 5
	11d	Relevant concomitant care and interventions that are permitted or prohibited during the trial	p. 9, line 20
Outcomes	12	Primary, secondary, and other outcomes, including the specific measurement variable (eg, systolic blood pressure), analysis metric (eg, change from baseline, final value, time to event), method of aggregation (eg, median, proportion), and time point for each outcome. Explanation of the clinical relevance of chosen efficacy and harm outcomes is strongly recommended	p. 13, line 9

Participant timeline	13	Time schedule of enrolment, interventions (including any run-ins and washouts), assessments, and visits for participants. A schematic diagram is highly recommended (see Figure)	p. 9, line 23 Figure 1
Sample size	14	Estimated number of participants needed to achieve study objectives and how it was determined, including clinical and statistical assumptions supporting any sample size calculations	p. 15, line 2
Recruitment	15	Strategies for achieving adequate participant enrolment to reach target sample size	p. 9, line 23
**Methods: Assignment of interventions (for controlled trials)**			
Allocation:			
Sequence generation	16a	Method of generating the allocation sequence (eg, computer-generated random numbers), and list of any factors for stratification. To reduce predictability of a random sequence, details of any planned restriction (eg, blocking) should be provided in a separate document that is unavailable to those who enrol participants or assign interventions	p. 10, line 10
Allocation concealment mechanism	16b	Mechanism of implementing the allocation sequence (eg, central telephone; sequentially numbered, opaque, sealed envelopes), describing any steps to conceal the sequence until interventions are assigned	p. 10, line 10
Implementation	16c	Who will generate the allocation sequence, who will enrol participants, and who will assign participants to interventions	p. 10, line 14
Blinding (masking)	17a	Who will be blinded after assignment to interventions (eg, trial participants, care providers, outcome assessors, data analysts), and how	p. 15, line 20
	17b	If blinded, circumstances under which unblinding is permissible, and procedure for revealing a participant's allocated intervention during the trial	p. 15, line 20
**Methods: Data collection, management, and analysis**			

Data collection methods	18a	Plans for assessment and collection of outcome, baseline, and other trial data, including any related processes to promote data quality (eg, duplicate measurements, training of assessors) and a description of study instruments (eg, questionnaires, laboratory tests) along with their reliability and validity, if known. Reference to where data collection forms can be found, if not in the protocol	p. 13, line 8<br>p. 15, line 13
	18b	Plans to promote participant retention and complete follow-up, including list of any outcome data to be collected for participants who discontinue or deviate from intervention protocols	p. 16, line 16
Data manageme nt	19	Plans for data entry, coding, security, and storage, including any related processes to promote data quality (eg, double data entry; range checks for data values). Reference to where details of data management procedures can be found, if not in the protocol	p. 15, line 17<br>p. 16, line 16
Statistical methods	20a	Statistical methods for analysing primary and secondary outcomes. Reference to where other details of the statistical analysis plan can be found, if not in the protocol	p. 16, line 1
	20b	Methods for any additional analyses (eg, subgroup and adjusted analyses)	p. 16, line 10
	20c	Definition of analysis population relating to protocol non-adherence (eg, as randomised analysis), and any statistical methods to handle missing data (eg, multiple imputation)	p. 16, line 9
**Methods: Monitoring**			
Data monitoring	21a	Composition of data monitoring committee (DMC); summary of its role and reporting structure; statement of whether it is independent from the sponsor and competing interests; and reference to where further details about its charter can be found, if not in the protocol. Alternatively, an explanation of why a DMC is not needed	p. 16, line 16
	21b	Description of any interim analyses and stopping guidelines, including who will have access to these interim results and make the final decision to terminate the trial	p. 16, line 3

Harms	22	Plans for collecting, assessing, reporting, and managing solicited and spontaneously reported adverse events and other unintended effects of trial interventions or trial conduct	p. 17, line 5
Auditing	23	Frequency and procedures for auditing trial conduct, if any, and whether the process will be independent from investigators and the sponsor	p. 16, line 17
**Ethics and dissemination**			
Research ethics approval	24	Plans for seeking research ethics committee/institutional review board (REC/IRB) approval	p. 17, line 17
Protocol amendments	25	Plans for communicating important protocol modifications (eg, changes to eligibility criteria, outcomes, analyses) to relevant parties (eg, investigators, REC/IRBs, trial participants, trial registries, journals, regulators)	p. 16, line 16<br>p. 17, line 21
Consent or assent	26a	Who will obtain informed consent or assent from potential trial participants or authorised surrogates, and how (see Item 32)	p. 10, line 7
	26b	Additional consent provisions for collection and use of participant data and biological specimens in ancillary studies, if applicable	N/A
Confidentiality	27	How personal information about potential and enrolled participants will be collected, shared, and maintained in order to protect confidentiality before, during, and after the trial	p. 15, line 13
Declaration of interests	28	Financial and other competing interests for principal investigators for the overall trial and each study site	p. 22, line 11
Access to data	29	Statement of who will have access to the final trial dataset, and disclosure of contractual agreements that limit such access for investigators	p. 17, line 2
Ancillary and post-trial care	30	Provisions, if any, for ancillary and post-trial care, and for compensation to those who suffer harm from trial participation	p. 17, line 14
Dissemination policy	31a	Plans for investigators and sponsor to communicate trial results to participants, healthcare professionals, the public, and other relevant groups (eg, via publication, reporting in results databases, or other data sharing arrangements), including any publication restrictions	p. 17, line 24

For peer review only

	31b	Authorship eligibility guidelines and any intended use of professional writers	p. 21, line 1
	31c	Plans, if any, for granting public access to the full protocol, participant-level dataset, and statistical code	p. 17, line 24
**Appendices**			
Informed consent materials	32	Model consent form and other related documentation given to participants and authorised surrogates	Supplementary materials
Biological specimens	33	Plans for collection, laboratory evaluation, and storage of biological specimens for genetic or molecular analysis in the current trial and for future use in ancillary studies, if applicable	N/A

*It is strongly recommended that this checklist be read in conjunction with the SPIRIT 2013 Explanation & Elaboration for important clarification on the items. Amendments to the protocol should be tracked and dated. The SPIRIT checklist is copyrighted by the SPIRIT Group under the Creative Commons "Attribution-NonCommercial-NoDerivs 3.0 Unported" license.

For peer review only
