# Supplementary material for: The HARMONIC trial: study protocol for a randomised controlled feasibility trial of Shaping Healthy Minds—a modular transdiagnostic intervention for mood, stressor-related and anxiety disorders in adults
Source: BMJ Open. 2018 Aug 5;8(8):e024546. doi: 10.1136/bmjopen-2018-024546 (PMC6078277; doi:10.1136/bmjopen-2018-024546)
Supplement: Reviewer comments [file bmjopen-2018-024546.reviewer_comments.pdf]

PEER REVIEW HISTORY

BMJ Open publishes all reviews undertaken for accepted manuscripts. Reviewers are asked to complete a checklist review form (**http://bmjopen.bmj.com/site/about/resources/checklist.pdf**) and are provided with free text boxes to elaborate on their assessment. These free text comments are reproduced below.

ARTICLE DETAILS

**TITLE (PROVISIONAL)**	The HARMONIC trial: Study protocol for a randomised controlled feasibility trial of Shaping Healthy Minds – a modular transdiagnostic intervention for mood, stressor-related and anxiety disorders in adults
**AUTHORS**	Black, Melissa; Hitchcock, Caitlin; Bevan, Anna; O Leary, Cliodhna; Clarke, James; Elliott, Rachel; Watson, Peter; LaFortune, Louise; Rae, Sarah; Gilbody, Simon; Kuyken, Willem; Johnston, David; Newby, Jill M.; Dalgleish, Tim

This article was not externally reviewed at BMJ Open. Protocols that have been independently assessed prior to submission to BMJ Open are usually fast-tracked to publication on the grounds that further substantial changes will not be possible. This independent assessment will usually be external, independent review for both a substantial grant award from a non-commercial or government funder and ethics approval.
